# Supplementary material for: Exponential concentration in quantum kernel methods
Source: Nat Commun. 2024 Jun 18;15:5200. doi: 10.1038/s41467-024-49287-w (PMC11189509; doi:10.1038/s41467-024-49287-w)
Supplement: Supplementary file 1 — Supplementary Information [file 41467_2024_49287_MOESM1_ESM.pdf]

Supplementary Information for *Exponential concentration in quantum kernel methods*

I. SUPPLEMENTARY NOTE - RELATED WORK

A. Exponential concentration in fidelity quantum kernels

The observation that using fidelity quantum kernel could lead to poor generalization was first made in Ref. [1]. In particular, this paper provided a rigorous generalization bound that could be used to compare the predictive power of quantum and classical kernel-based models. Based on this bound, the authors argued that for high dimensional problems the embedded states are likely to be 'far from each other' and so have either comparable or inferior performance compared with its classical counterparts. This work also provided numerical evidence of this observation for up to 30 qubits. Similar numerical evidence was provided more recently in Ref. [2].

Subsequently, Ref. [3] argued that not only are quantum models unable to outperform classical models but, more generally, embeddings that lack an inductive bias lead to models that generalize poorly. To demonstrate this, the authors analyzed spectral properties of the quantum fidelity kernel integral operator. Specifically, they lower bounded a model's generalization error in terms of the largest eigenvalue of the kernel integral operator (Theorem 3 in Appendix D). This result is then used to show that fidelity kernels with an unstructured product embedding will lead to large risks and so poor generalization (Theorem 1). Since this result holds for a product embedding, in our language this could be viewed as globality induced concentration. While not shown explicitly in Ref. [3] it is plausible that Theorem 3 could also be used as an alternative approach to proving that highly expressive or entangling embeddings lead to poor generalization.

We note that while both these important works highlight problems with the fidelity kernel related to exponential concentration, the exact causes of exponential concentration were not analysed in details. Furthermore, in both cases, the detrimental effect of exponential concentration was studied assuming direct access to quantum states without shot noise.

B. Exponential concentration in projected quantum kernels

As a potential solution to the problems with the fidelity quantum kernel, the authors in Ref. [1] introduced the projected quantum kernel where the data encoded quantum states are projected back onto local subspaces with the similarity between quantum states being collectively compared at the local level as defined in Eq. (4) in the main text. The projected quantum kernel can be challenging to evaluate (having gone through the exponentially large Hilbert space before projection) and yet remain reasonably expressive. The authors showed that for a synthesized dataset a model based on the projected quantum kernel can outperform a wide range of classical machine learning models and numerically verified this up to 30 qubits.

Nevertheless, there remain some open questions including how exactly expressivity of the data embedding can affect the performance of the projected quantum kernel and whether too much expressivity can lead to exponential concentration or not. In Ref. [3] the authors considered a simplified version of the original projected quantum kernel defined as the overlap between two reduced data encoded states onto the first qubit. The authors then argued that the projected quantum kernel with an embedding consisting of a layer of data-dependent single qubit rotations followed by a fixed data-independent Haar random unitary could have an inductive bias that is hard to simulate classically. However, the authors then prove that the embedded quantum states exponentially concentrate towards the maximally mixed state. Thus they suggest (but do not explicitly prove) that exponentially many measurement shots will be needed for such embeddings (similarly to the barren plateaus phenomena in QNNs). Crucially, exponential concentration here does not directly come from the randomness in the input data distribution but rather from a data-independent part of the embedding. In contrast to this, our work concerns the expressivity induced by the interplay between the input data distribution and the embedding. Additionally, we consider an arbitrary data-dependent embedding and the original form of the projected quantum kernel (but our results can be easily extended to other forms of the projected kernel including the one in Ref. [3]). More generally, we identify noise and entanglement as additional sources of exponential concentration for projected kernels and analyse the consequences of exponential concentration in the presence of shot noise for model predictions.

C. Attempts to mitigate the exponential concentration

One proposal to mitigate exponential concentration for the fidelity quantum kernel is to re-scale the input data with some hyperparameter [4, 5]. Consequently, the data encoded quantum states become clustered closer together and hence result in a lower expressivity. This idea was first numerically demonstrated in Ref. [4] and analytical treatment on how this hyperparameter affects the kernel spectrum was done in the follow-up work in Ref. [5]. While this could ensure that the fidelity kernel does not suffer from expressivity induced concentration, it was shown later in Ref. [2] with numerical simulations of up to 20 qubits that this approach is unlikely to provide any quantum advantage over classical models.

Ref. [6] proposed a new type of quantum kernel known as the quantum Fisher kernel. The kernel encodes geometric information of the input data. The author considered the alternating layered ansatz where the embedding consists of layers of unitary blocks that act on local qubits and analytically showed the absence of exponential concentration with log depth layers. We note that extending the layers to linear depth still leads to a highly expressive embedding and exponential concentration.

Another line of work aims to optimize a parameterized embedding for a quantum kernel when there is no prior knowledge of the data structure. Ref. [7] used kernel target alignment (as defined in Eq. (36) in the main text) to align a parameterized embedding with an ideal embedding approximated with the given training data. In Ref. [8], the authors rely on a more heuristic approach to slowly build the embedding from a set of unitary blocks, which is similar to a layer-wise training strategy in variational quantum algorithms. We note that, as shown in the main text, that training the embedding can potentially lead to barren plateaus when the trainable part of the embedding is not constructed properly.

D. Effect of shot noise in the absence of exponential concentration

Ref. [9] proved a quantum advantage for a quantum support vector machine using a fidelity quantum kernel for solving a particular classification task where the dataset is engineered based on the discrete log problem. As the discrete log problem is strongly believed to be inefficient for classical computers and efficient for quantum computers, the authors proved that this classical hardness carries over to the learning task. Thus using a carefully constructed embedding with a strong inductive bias aligned to the problem structure allows for a quantum advantage. Importantly, the advantage remains in the presence of shot noise. In particular, the kernel values and model predictions can be efficiently evaluated with a polynomial number of measurement shots.

In Ref. [10], the effect of shot noise in quantum support vector machines was studied for an arbitrary classification task. It was analytically shown that in the absence of exponential concentration and under the assumption that a separation between two classes is polynomially large the performance of a kernel model is robust against shot noise.

II. SUPPLEMENTARY NOTE - PRELIMINARIES FOR STATISTICAL INDISTINGUISHABILITY

Here we quote some key technical tools on the distinguishability of probability distributions from binary hypothesis testing, which we will use to establish how the exponential concentration affects the kernel methods in Supplementary Notes III A 2 and III B. For a more extensive exposition we refer the reader to Ref. [11].

A. One sample

Supplemental Lemma 1. *Consider two probability distributions $\mathcal{P}$ and $\mathcal{Q}$ over some finite set of outcomes $\mathcal{I}$. Suppose we are given a single sample S drawn from either $\mathcal{P}$ or $\mathcal{Q}$ with equal probability, and we have the following two hypotheses:*

- *Null hypothesis $\mathcal{H}_0$: S is drawn from $\mathcal{P}$,*
- *Alternative hypothesis $\mathcal{H}_1$: S is drawn from $\mathcal{Q}$.*

The probability of correctly deciding the true hypothesis is upper bounded as

$$\Pr[\text{“right decision between}\, \mathcal{H}_0 \,\text{and}\, \mathcal{H}_1\text{”}] \leqslant \frac{1}{2} + \frac{\|\mathcal{P}-\mathcal{Q}\|_1}{4} \,, \tag{1}$$

where we denote $\|\mathcal{P}-\mathcal{Q}\|_1 = \sum_{s\in\mathcal{I}} |p(s)-q(s)|$ *as the 1-norm between the probability vectors (2 × the total variation distance).*

Proof. There exists a region $\mathcal{A}$ such that $p(s) > q(s)$ for all $s \in \mathcal{A}$. The optimal decision making strategy is to choose that the given sample S is drawn from $\mathcal{P}$ if it falls in the region i.e., $S \in \mathcal{A}$ and choose $\mathcal{Q}$, otherwise. The probability of making the right decision can be expressed as

$$\begin{aligned}\Pr[\text{“right decision between}\, \mathcal{H}_0 \,\text{and}\, \mathcal{H}_1\text{”}] =& \Pr(S\in\mathcal{A}|S\sim\mathcal{P})\Pr(S\in\mathcal{P}) + \Pr(S\notin\mathcal{A}|S\sim\mathcal{Q})\Pr(S\in\mathcal{Q}) &(2)\\ =&\frac{1}{2}\left[\Pr(S\in\mathcal{A}|S\sim\mathcal{P}) + \Pr(S\notin\mathcal{A}|S\sim\mathcal{Q})\right] &(3)\\ =&\frac{1}{2}\left[\sum_{s\in\mathcal{A}} p(s) + \sum_{s\notin\mathcal{A}} q(s)\right] \,, &(4)\end{aligned}$$

where the second equality is due to the sample being equally likely to be drawn from either $\mathcal{P}$ or $\mathcal{Q}$. In the last equality, we use the fact that given that the sample is from $\mathcal{P}$, the probability that this sample takes any value within the region $\mathcal{A}$ is simply $\sum_{s\in\mathcal{A}} p(s)$, and similarly for $s \notin \mathcal{A}$.

The 1-norm between probability vectors can be written as

$$\begin{aligned}\|\mathcal{P}-\mathcal{Q}\|_1 =& \sum_{s\in\mathcal{I}} |p(s)-q(s)| &(5)\\ =& \sum_{s\in\mathcal{A}} (p(s)-q(s)) + \sum_{s\notin\mathcal{A}} (q(s)-p(s)) \,, &(6)\end{aligned}$$

where we have separated terms in the sum based on the region $\mathcal{A}$. Lastly, we notice that

$$\begin{aligned}\frac{2+\|\mathcal{P}-\mathcal{Q}\|_1}{2} =& \frac{1}{2}\left(\sum_{s\in\mathcal{I}} p(s) + \sum_{s\in\mathcal{I}} q(s) + \|\mathcal{P}-\mathcal{Q}\|_1\right) &(7)\\ =& \sum_{s\in\mathcal{A}} p(s) + \sum_{s\notin\mathcal{A}} q(s) \,, &(8)\end{aligned}$$

where in the second line we have used Eq. (6). Substituting this back to Eq. (4), we obtain the desired result. □

B. Many samples

We now consider the scenario where instead of a single sample we are given N samples from either $\mathcal{P}$ and $\mathcal{Q}$ is given to us and we have to guess which of the two distributions these samples are drawn from. At first glance, it may seem that Lemma 1 is not applicable to this scenario, since we now have a set of outcomes rather than just one sample. However, we can consider the product distributions $\mathcal{P}^{\otimes N}$ and $\mathcal{Q}^{\otimes N}$, where a single sample corresponds to N samples from $\mathcal{P}$ and $\mathcal{Q}$ respectively.

We first state a generic inequality on product distributions.

Supplemental Lemma 2. *The 1-norm between discrete product distributions* $\mathcal{P}^{\otimes N}$ *and* $\mathcal{Q}^{\otimes N}$ *can be upper bounded as*

$$\|\mathcal{P}^{\otimes N} - \mathcal{Q}^{\otimes N}\|_1 \leqslant N\|\mathcal{P}-\mathcal{Q}\|_1 \,. \tag{9}$$

Proof. We have

$$\|\mathcal{P}^{\otimes N} - \mathcal{Q}^{\otimes N}\|_1 = \|\mathcal{P}^{\otimes N} - \mathcal{Q}\otimes\mathcal{P}^{\otimes N-1} + \mathcal{Q}\otimes\mathcal{P}^{\otimes N-1} - \mathcal{Q}^{\otimes 2}\otimes\mathcal{P}^{\otimes N-2} + \ldots + \mathcal{Q}^{\otimes N-1}\otimes\mathcal{P} - \mathcal{Q}^{\otimes N}\|_1 \tag{10}$$

$$\leqslant \|\mathcal{P}^{\otimes N} - \mathcal{Q}\otimes\mathcal{P}^{\otimes N-1}\|_1 + \|\mathcal{Q}\otimes\mathcal{P}^{\otimes N-1} - \mathcal{Q}^{\otimes 2}\otimes\mathcal{P}^{\otimes N-2}\|_1 + \ldots + \|\mathcal{Q}^{\otimes N-1}\otimes\mathcal{P} - \mathcal{Q}^{\otimes N}\|_1 \tag{11}$$

$$= \|\mathcal{P}-\mathcal{Q}\|_1\,\|\mathcal{P}^{\otimes N-1}\|_1 + \|\mathcal{Q}\|_1\,\|\mathcal{P}-\mathcal{Q}\|_1\,\|\mathcal{P}^{\otimes N-2}\|_1 + \ldots + \|\mathcal{Q}^{\otimes N-1}\|_1\,\|\mathcal{P}-\mathcal{Q}\|_1 \tag{12}$$

$$= N\|\mathcal{P}-\mathcal{Q}\|_1 \ , \tag{13}$$

where in the first line we have added and subtracted terms, the inequality is due to the triangle inequality, and the third line is due to the fact that the 1-norm factorizes. □

Supplemental Lemma 2 along with Supplemental Lemma 1 immediately implies an upper bound on a hypothesis testing experiment using N samples. In the following proposition we specify this for binary distributions.

Supplemental Proposition 1. *Consider two binary probability distributions $\mathcal{P}_0 = (p_0, 1-p_0)$ and $\mathcal{P}_\varepsilon = (p_\varepsilon, 1-p_\varepsilon)$ where $p_\epsilon = p_0 + \varepsilon$. Suppose we are given a N samples (denoted $\mathcal{M}$) drawn from either $\mathcal{P}_0$ or $\mathcal{P}_\varepsilon$ with equal probability, and we have the following two hypotheses:*

- *Null hypothesis $\mathcal{H}_0$: $\mathcal{M}$ is drawn from $\mathcal{P}_0$,*
- *Alternative hypothesis $\mathcal{H}_1 : \mathcal{M}$ is drawn from $\mathcal{P}_\varepsilon$.*

The probability of correctly deciding the true hypothesis is upper bounded as

$$\Pr[\text{“right decision between } \mathcal{H}_0 \text{ and } \mathcal{H}_1\text{”}] \leqslant \frac{1}{2} + \frac{N|\varepsilon|}{2} \ . \tag{14}$$

Proof. We remark that the combination of Supplemental Lemma 2 along with Supplemental Lemma 1 gives success probability

$$\Pr[\text{“right decision between } \mathcal{H}_0 \text{ and } \mathcal{H}_1\text{”}] \leqslant \frac{1}{2} + \frac{N\|\mathcal{P}_0 - \mathcal{P}_\varepsilon\|_1}{4} \tag{15}$$

and explicit evaluation shows that $\|\mathcal{P}_0 - \mathcal{P}_\epsilon\|_1 = 2|\varepsilon|$. □

In the rest of this work, we mainly focus on a perturbation that is exponentially small in the system size n, i.e., $\epsilon \in \mathcal{O}(1/b^n)$ for some $b > 1$.

III. SUPPLEMENTARY NOTE - PRACTICAL IMPLICATIONS OF EXPONENTIAL CONCENTRATION ON KERNEL METHODS

In this section we analyse the consequences of exponential concentration on kernel methods. Specifically, we show that when using a polynomial number of measurements the statistical estimate of the Gram matrix is with high probability independent of input data. Consequently, training with the estimated Gram matrix results in a data-independent model. It then follows that the final (trained) output model is independent of the training data and cannot generalize. To make this argument more concrete we consider the example of kernel ridge regression; however the fundamental problem of the data-independence of the output prediction caries over similarly to other learning tasks.

We present this argument for both the fidelity quantum kernel and projected quantum kernel, for different corresponding strategies to prepare them. The rest of this section is structured as follows.

In Supplementary Note III A we discuss the fidelity quantum kernel where two measurement strategies to estimate kernel values are considered.

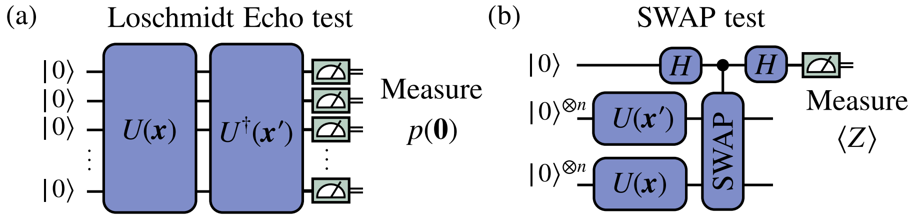

Supplementary Figure 1. **Schematic diagram of tests.** We illustrate two different strategies to estimate kernel values. In panel (a) we show the Loschmidt Echo test where the estimated kernel value is equivalent to the empirical probability of measuring the all-zero bitstring. In panel (b) we show the SWAP test where the kernel value is estimated with the expectation value of Pauli Z operator on an ancilla qubit.

- Supplementary Note III A 1 concerns with the Loschmidt Echo test to estimate kernel values. In the presence of exponential concentration, we rigorously show that the statistical estimates of the kernel values concentrate at zero with high probability (see Supplemental Proposition 2). As a consequence, the estimated Gram matrix is likely to simply be the identity matrix and the estimated model predictions also concentrate to zero with high probability (see Supplemental Corollary 1).

- Supplementary Note III A 2 is concerned with the SWAP test to estimate kernel values. Here, we rely on a reduction to hypothesis testing (see Supplementary Note II for preliminaries), and we define notions of statistical indistinguishability (see Definition 2 for distributions and Definition 3 for outputs). When kernel values exponentially concentrate, their estimates become statistically indistinguishable with high probability (see Supplemental Lemma 3). Consequently, outputs of the model trained on these kernel estimates are also indistinguishable and insensitive to unseen input data (see Supplemental Corollary 2).

- In Supplementary Note III A 3 we present numerical simulations of the fidelity kernel to support the theoretical results in the previous sub-sections.

We then discuss the projected quantum kernel in Supplementary Note III B and investigate two measurement strategies to obtain kernel estimates.

- Supplementary Note III B 1 is concerned with the practical consequence of exponential concentration. For both measurement strategies, the effect appears in an identical manner as in the SWAP test for the fidelity kernel. That is, we have statistical indistinguishability of kernel estimates (see Supplemental Proposition 3) and their model predictions (see Corollary 3) . Since the setting in the projected kernel is more complicated than the fidelity kernel with the SWAP test, we encourage interested readers to first review Supplementary Note III A 2 as the ideas are similar (or Supplementary Note II for preliminaries on hypothesis testing).

- In Supplementary Note III B 2, we illustrate numerical results to back up our theoretical findings.

- In order to not interrupt the flow when going through the first two sub-sections, we group all the proofs together in Supplementary Note III B 3

In Supplementary Note III C, we discuss the extension of kernel concentration to state concentration. This leads to a stronger concentration result which cannot be resolved even with quantum access to polynomial state copies. Lastly, in Supplementary Note III D, we provide discussion on a sufficient condition to resolve the exponential concentration issue. We found that the number of measurement shots has to scale exponentially in the number of qubits in order to acquire enough resolution in the kernel estimates. This exponential scaling is impractical for large problem sizes.

A. Fidelity quantum kernel

Let us start by recalling that an input $\boldsymbol{x}$ is encoded into a data-encoding quantum state $\rho(\boldsymbol{x})$ through an embedding unitary $U(\boldsymbol{x})$ and the fidelity quantum kernel is of the form

$$\kappa^{\mathrm{FQ}}(\boldsymbol{x}, \boldsymbol{x'}) = \mathrm{Tr}[\rho(\boldsymbol{x})\rho(\boldsymbol{x'})] \ . \tag{16}$$

The exact value of the kernel is inaccessible and instead we obtain a statistical estimate using measurement outcomes/shots from quantum computers. There are two common measurement strategies to estimate the fidelity quantum kernel: (i) the Loschmidt Echo test or (ii) the SWAP test, as shown in Fig. 1. In either case, the fidelity quantum kernel is equivalent to the expectation value of an observable O with some quantum state ρ with the exact expression for O and ρ depending on the strategy used. If we write the eigendecomposition of the observable as $O = \sum_i o_i |o_i\rangle\langle o_i|$ where o_i and $|o_i\rangle$ are eigenvalues and eigenvectors of O, then the statistical estimate after N measurements is of the form

$$\widehat{\kappa}^{\mathrm{FQ}}(\boldsymbol{x}, \boldsymbol{x'}) = \frac{1}{N}\sum_{m=1}^{N} \lambda_m \ , \tag{17}$$

where λ_m is the outcome of the mth measurement and can be treated as a random variable which takes the value o_i with probability $p_i = \mathrm{Tr}[|o_i\rangle\langle o_i|\rho]$.

We now restate the definition of exponential concentration discussed in the main text.

Definition 1 (Exponential concentration)**.** *Consider a quantity $X(\boldsymbol{\alpha})$ that depends on a set of variables $\boldsymbol{\alpha}$ and can be measured from a quantum computer as the expectation of some observable. $X(\boldsymbol{\alpha})$ is said to be deterministically exponentially concentrated in the number of qubits n towards a certain $\boldsymbol{\alpha}$-independent value μ if*

$$|X(\boldsymbol{\alpha}) - \mu| \leqslant \beta \in O(1/b^n) \ , \tag{18}$$

for some $b > 1$ and all $\boldsymbol{\alpha}$. Analogously, $X(\boldsymbol{\alpha})$ is probabilistically exponentially concentrated if

$$\mathrm{Pr}_{\boldsymbol{\alpha}}[|X(\boldsymbol{\alpha}) - \mu| \geqslant \delta] \leqslant \frac{\beta}{\delta^2} \ , \ \beta \in O(1/b^n) \ , \tag{19}$$

for $b > 1$. That is, the probability that $X(\boldsymbol{\alpha})$ deviates from μ by a small amount δ is exponentially small for all $\boldsymbol{\alpha}$.

In addition, if μ exponentially vanishes in the number of qubits i.e., $\mu \in \mathcal{O}(1/b'^n)$ for some $b' > 1$, we say that $X(\boldsymbol{\alpha})$ exponentially concentrates towards an exponentially small value.

In the context of quantum kernels, $X(\boldsymbol{\alpha}) = \kappa^{\mathrm{FQ}}(\boldsymbol{x}, \boldsymbol{x'})$ with the set of variables corresponding to an input data pair $\boldsymbol{\alpha} = \{\boldsymbol{x}, \boldsymbol{x'}\}$. When μ vanishes exponentially, we remark that the probability of deviating from zero by an arbitrary constant amount is exponentially small.

We note that Supplemental Proposition 2 (and the first part of Supplemental Corollary 2) is a full version of Proposition 1 (and Proposition 2) in the main text, which concern about the practical implications on estimated kernel values and the Gram matrix. Additionally, a full statement of Corollary 1 which considers the impact of kernel concentration on model predictions is presented in Supplemental Corollary 1 and the latter half of Supplemental Corollary 2.

1. *Loschmidt Echo test*

For the Loschmidt Echo test, the quantum fidelity kernel is the probability of measuring the all-zero bitstring. That is, the observable is the global projector for the all-zero state $O = |\mathbf{0}\rangle\langle\mathbf{0}|$ and $\rho = U^\dagger(\boldsymbol{x'})U(\boldsymbol{x})|\mathbf{0}\rangle\langle\mathbf{0}|U^\dagger(\boldsymbol{x})U(\boldsymbol{x'})$. The measurement outcome is $+1$ when the all-zero bitstring is observed and is 0 for any other bitstrings. Thus, the statistical estimate is simply the ratio of the number of observed all-zero bitstrings to the total number of measurements. When the kernel value exponentially concentrates an exponentially small quantity, the statistical estimate of the kernel is 0 with a probability exponentially close to 1. This is shown in the following proposition.

Supplemental Proposition 2 (A full version of Proposition 1)**.** *Consider the fidelity quantum kernel as defined in Eq.* (16)*. Assume that the kernel values* $\kappa^{\rm FQ}(\boldsymbol{x},\boldsymbol{x'})$ *exponentially concentrate towards some exponentially small value* μ*. Given that the Loschmidt Echo test is used to estimate the kernel value between an input data pair* $\boldsymbol{x}$ *and* $\boldsymbol{x'}$ *with a polynomial number of measurement shots* N*, the probability that the statistical estimate of the kernel value* $\widehat{\kappa}^{\rm FQ}(\boldsymbol{x},\boldsymbol{x'})$ *is zero is exponentially close to* 1*. That is,*

$$\Pr\left[\widehat{\kappa}^{\rm FQ}(\boldsymbol{x},\boldsymbol{x'})=0\right]\geqslant 1-\delta \ \ , \ \delta\in\mathcal{O}(c^{-n}) \tag{20}$$

for some constant $c>1$*. In addition, for any training dataset* $\mathcal{S}=\{\boldsymbol{x}_i,y_i\}$ *of size* $N_s\in\mathcal{O}({\rm poly}(n))$*, with a probability exponentially close to* 1*, the statistical estimate of the Gram matrix* $\widehat{K}$ *is equal to the identity matrix. That is,*

$$\Pr[\widehat{K}=\mathbb{1}]\geqslant 1-\delta' \ \ , \ \delta'\in\mathcal{O}(c'^{-n}) \tag{21}$$

for some constant $c'>1$*.*

Proof. First, we recall that if the fidelity kernel concentrates to some exponentially small value over possible input data pairs as per Definition 1, we have

$$\Pr_{\boldsymbol{x},\boldsymbol{x'}}\left[\left|\kappa^{\rm FQ}(\boldsymbol{x},\boldsymbol{x'})-\mu\right|\geqslant\delta_c\right]\leqslant\frac{\beta}{\delta_c^2}\ , \tag{22}$$

such that

$$\mu\in\mathcal{O}(1/b'^n)\ , \tag{23}$$

for some $b'>1$, and

$$\beta\in\mathcal{O}(1/b^n)\ , \tag{24}$$

for some $b>1$. By specifying $\delta_c=\beta^{1/4}$ and inverting the inequality, we have

$$\Pr_{\boldsymbol{x},\boldsymbol{x'}}\left[\left|\kappa^{\rm FQ}(\boldsymbol{x},\boldsymbol{x'})-\mu\right|\leqslant\beta^{1/4}\right]\geqslant 1-\sqrt{\beta}\ . \tag{25}$$

This implies that the probability of $\kappa^{\rm FQ}(\boldsymbol{x},\boldsymbol{x'})$ to be between $\mu-\beta^{1/4}$ and $\mu+\beta^{1/4}$ is at least $1-\sqrt{\beta}$.

We now show that for any given pair of $\boldsymbol{x}$ and $\boldsymbol{x'}$, it is exponentially likely that the statistical estimate of the kernel is zero. This is equivalent to proving that none of obtained bitstrings is all-zero bitstring. After N measurements, the probability of this event happening can be expressed as

$$\begin{aligned}
\Pr[\widehat{\kappa}^{\rm FQ}(\boldsymbol{x},\boldsymbol{x'})=0] &= \int_0^1 \Pr\left[\widehat{\kappa}^{\rm FQ}(\boldsymbol{x},\boldsymbol{x'})=0\middle|\kappa^{\rm FQ}(\boldsymbol{x},\boldsymbol{x'})=s\right]\Pr\left[\kappa^{\rm FQ}(\boldsymbol{x},\boldsymbol{x'})=s\right]ds && (26)\\
&= \int_0^1 (1-s)^N \Pr\left[\kappa^{\rm FQ}(\boldsymbol{x},\boldsymbol{x'})=s\right]ds && (27)\\
&\geqslant \int_{\mu-\beta^{1/4}}^{\mu+\beta^{1/4}} (1-s)^N \Pr\left[\kappa^{\rm FQ}(\boldsymbol{x},\boldsymbol{x'})=s\right]ds && (28)\\
&\geqslant (1-(\mu+\beta^{1/4}))^N \int_{\mu-\beta^{1/4}}^{\mu+\beta^{1/4}} \Pr\left[\kappa^{\rm FQ}(\boldsymbol{x},\boldsymbol{x'})=s\right]ds && (29)\\
&\geqslant (1-(\mu+\beta^{1/4}))^N(1-\sqrt{\beta}) && (30)\\
&\geqslant (1-N(\mu+\beta^{1/4}))(1-\sqrt{\beta})\ , && (31)
\end{aligned}$$

where in the first equality Bayes' theorem is used to introduce the conditional probability of measuring none all-zero bitstring for given $s=\kappa^{\rm FQ}(\boldsymbol{x},\boldsymbol{x'})$ and the marginal probability is acquired by integrating all possible values of $\kappa^{\rm FQ}(\boldsymbol{x},\boldsymbol{x'})$.

The second equality is due to the fact that measurement outcomes are independent. In the first inequality, we limit the range of integration to $\mu \pm \beta^{1/4}$. The second inequality is from taking the minimum value of $(1-s)$ within the integration range. The next inequality is due to Eq. (25). To reach the last line in Eq. (31), we apply Bernoulli's inequality.

Now, it remains to show that the lower bound in Eq. (31) is exponentially close to 1. We recall that if the kernel values exponentially concentrate towards some exponentially small value, we have that μ and β follow Eq. (23) and Eq. (24). Lastly, for a polynomial number of measurement shots, i.e., $N \in \mathcal{O}(\text{poly}(n))$, we have that $N(\mu + \beta^{1/4})$ vanishes exponentially, leading to

$$\Pr\left[\widehat{\kappa}^{\text{FQ}}(\boldsymbol{x}, \boldsymbol{x'}) = 0\right] \geqslant 1 - \delta \ \ , \ \delta \in \mathcal{O}(c^{-n}) \ , \tag{32}$$

with $\delta = N(\mu + \beta^{1/4}) + \sqrt{\beta} - N(\mu + \beta^{1/4})\sqrt{\beta} \in \mathcal{O}(c^{-n})$ for some $c > 1$. For large n, δ becomes exponentially smaller than 1. This completes the first half of the proof.

We now proceed to the second half of the proof. Consider a training dataset $\mathcal{S} = \{\boldsymbol{x}_i, y_i\}$ with $N_s \in \mathcal{O}(\text{poly}(n))$. The event that the statistical estimate of the Gram matrix $\widehat{K}$ is equal to identity is equivalent to the event that all statistical estimates of kernel values for all pairs $\boldsymbol{x}_i$ and $\boldsymbol{x}_j$ (such that $i \neq j$) are zeros. Since each data point in the training dataset is drawn independently, estimating kernel values from different input data pairs in $\mathcal{S}$ are independent events

$$\begin{aligned}
\Pr\left[\widehat{K} = \mathbb{1}\right] &= \Pr\left[\widehat{\kappa}^{\text{FQ}}(\boldsymbol{x}_i, \boldsymbol{x}_j) = 0 \ ; \forall i, j \ , \ i \neq j\right] && (33)\\
&= \prod_{i<j} \Pr\left[\widehat{\kappa}^{\text{FQ}}(\boldsymbol{x}_i, \boldsymbol{x}_j) = 0\right] && (34)\\
&\geqslant (1-\delta)^{N_s(N_s-1)/2} && (35)\\
&\geqslant 1 - N_s(N_s-1)\delta/2 \ , && (36)
\end{aligned}$$

where the second equality uses the fact that the individual kernel values correspond to independent events (note that since the Gram matrix is symmetric we only have to estimate $N_s(N_s-1)/2$ kernel values). The first inequality is from applying the result in Eq. (32) (as the kernel values are concentrated) and the last inequality is from Bernoulli's inequality. Since $N_s \in \mathcal{O}(\text{poly}(n))$, we have that $\delta' = N_s(N_s-1)\delta/2 \in \mathcal{O}(c^{-n})$ for some $c > 1$. □

Supplemental Proposition 2 rigorously shows that the estimated Gram matrix is, *for any choice in input data*, is likely to be an identity matrix. It follows that the trained model will also, with high probability, be independent of the training data, and thus in all likelihood not very useful. To demonstrate this, we consider the example of kernel ridge regression and show that the predictions of the output model concentrate at zero with high probability.

Supplemental Corollary 1 (A full version of the Loschmidt Echo part of Corollary 1 in the main text)**.** *Consider implementing kernel ridge regression with the fidelity quantum kernel, a squared loss function and a training dataset $\mathcal{S} = \{\boldsymbol{x}, y_i\}_i$ with $N_s \in \mathcal{O}(\text{poly}(n))$. Denote an input data independent fixed point $\boldsymbol{a}_0(\boldsymbol{y}, \lambda) = \boldsymbol{y}/(1-\lambda)$ where $\boldsymbol{y}$ is a vector of output data points with its ith element equal to y_i and λ is regularization in the loss function. Under the same assumptions as in Supplemental Proposition 2, if the model is trained with a statistical estimate of the Gram matrix $\widehat{K}$ where each element is evaluated with a polynomial number of measurement shots $N \in \mathcal{O}(\text{poly}(n))$ then, with probability exponentially close to 1, the optimal parameters $\boldsymbol{a}_{\text{opt}}$ are identical to the fixed point $\boldsymbol{a}_0(\boldsymbol{y}, \lambda)$. That is, we have*

$$\Pr\left[\boldsymbol{a}_{\text{opt}} = \boldsymbol{a}_0(\boldsymbol{y}, \lambda)\right] \geqslant 1 - \delta \ \ , \ \delta \in \mathcal{O}(c^{-n}) \ , \tag{37}$$

for some constant $c > 1$. In addition, the estimated model prediction on an unseen input $\widehat{f}(\boldsymbol{x}) = \sum_i a_{\text{opt}}^{(i)} \widehat{\kappa}^{\text{FQ}}(\boldsymbol{x}_i, \boldsymbol{x})$ will, with a probability exponentially close to 1, be 0,

$$\Pr\left[\widehat{f}(\boldsymbol{x}) = 0 | \boldsymbol{x} \notin \mathcal{S}\right] \geqslant 1 - \delta' \ \ , \ \delta' \in \mathcal{O}(c'^{-n}) \ , \tag{38}$$

for some constant $c' > 1$.

Proof. According to Supplemental Proposition 2, we obtain the statistical estimate of the Gram matrix to be an identity $\widehat{K} = \mathbb{1}$ with probability exponentially close to 1. The optimal parameters for a kernel ridge regression with a squared loss function are given by

$$\boldsymbol{a}_{\text{opt}} = (\widehat{K} - \lambda\mathbb{1})^{-1}\boldsymbol{y} \tag{39}$$

$$= \frac{\boldsymbol{y}}{1-\lambda} \; . \tag{40}$$

For Supplemental Proposition 2, this is obtained with probability at least $1-\delta$ with $\delta \in \mathcal{O}(\text{poly}(n))$. This proves the first part of the corollary.

Secondly, it follows from the Representer Theorem that the model prediction is of the form

$$f(\boldsymbol{x}) = \sum_{i=1}^{N_s} a_{\text{opt}}^{(i)} \kappa^{\text{FQ}}(\boldsymbol{x}_i, \boldsymbol{x}) \; . \tag{41}$$

Computing the model prediction for an unseen input data $\boldsymbol{x} \notin \mathcal{S}$ requires computing a statistical estimate of the kernel values between the new data point and the training data points. When computed with a polynomial number of shots these estimates $\widehat{\kappa}^{\text{FQ}}(\boldsymbol{x_i}, \boldsymbol{x})$ will be 0 with high probability. Specifically, we can bound this probability as

$$\Pr\left[\widehat{f}(\boldsymbol{x}) = 0 | \boldsymbol{x} \notin \mathcal{S}\right] = \Pr\left[\widehat{\kappa}^{\text{FQ}}(\boldsymbol{x}, \boldsymbol{x}_i) = 0; \forall i\right] \tag{42}$$

$$= \prod_{i=1}^{N_s} \Pr\left[\widehat{\kappa}^{\text{FQ}}(\boldsymbol{x}, \boldsymbol{x}_i) = 0\right] \tag{43}$$

$$\geqslant (1-\delta)^{N_s} \tag{44}$$

$$\geqslant 1 - N_s\delta \; , \tag{45}$$

where the second equality is due to the statistical independence of the kernel value estimates, the first inequality is from applying Supplemental Proposition 2 and the final inequality is from Bernoulli's inequality. Since $N_s \in \mathcal{O}(\text{poly}(n))$, we have that $\delta' = N_s\delta \in \mathcal{O}(c^{-n})$ for some $c > 1$. □

Importantly, since all information concerning the training output data is in effect hard-coded in the formula for the optimal parameters, Eq. (39), a low training error can be obtained. On the other hand, the model prediction is entirely insensitive to the input data and hence the model generalizes poorly. As supported by the numerics in the main text (see Fig. 3 there), this poor generalization has a different flavor to the type of generalization usually quantified by generalization bounds in that it cannot be resolved simply by training on more data points. Instead, one must supply at least an exponential number of shots for hope of good generalization.

2. *SWAP test*

For the SWAP test, the kernel value is the expectation value of the Pauli Z operator on an ancilla qubit. For each measurement, the outcome is drawn from a certain distribution $\mathcal{P}_{\kappa^{\text{FQ}}(\boldsymbol{x},\boldsymbol{x'})}$ that encodes the kernel information. More precisely, the outcome is either $+1$ with probability $p_+ = 1/2 + \kappa^{\text{FQ}}(\boldsymbol{x}, \boldsymbol{x'})/2$ or -1 with probability $p_- = 1 - p_+$ i.e.,

$$\mathcal{P}_{\kappa^{\text{FQ}}(\boldsymbol{x},\boldsymbol{x'})} := \left\{ \frac{1 + \kappa^{\text{FQ}}(\boldsymbol{x}, \boldsymbol{x'})}{2}, \frac{1 - \kappa^{\text{FQ}}(\boldsymbol{x}, \boldsymbol{x'})}{2} \right\} \; . \tag{46}$$

In other words, the kernel value can be thought of as encoding a perturbation to the uniform distribution

$$\mathcal{P}_0 := \left\{ \frac{1}{2}, \frac{1}{2} \right\} \; . \tag{47}$$

We will consider the following notion of statistical indistinguishability (also illustrated in Fig. 2).

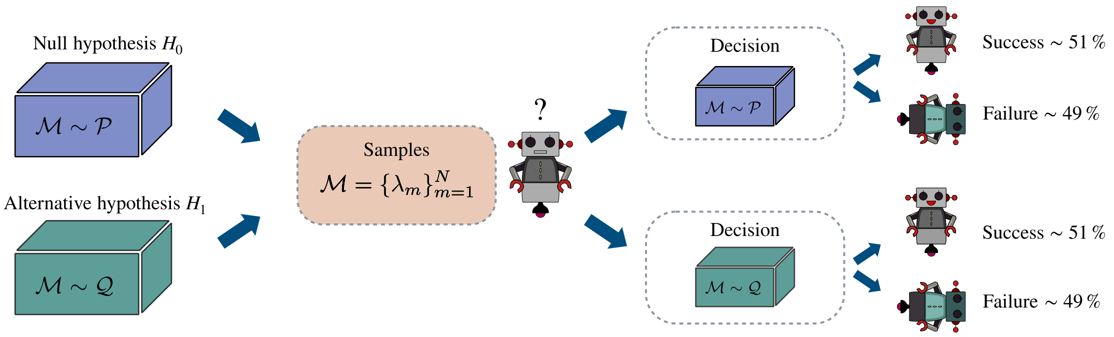

Supplementary Figure 2. **Statistical indistinguishability.** Suppose we are given a set of N samples $\mathcal{M}$ that are either drawn from $\mathcal{P}$ (Null hypothesis) or from $\mathcal{Q}$ (Alternative hypothesis), with the two possibilities equally probable. Two distributions $\mathcal{P}$ and $\mathcal{Q}$ are said to be statistically indistinguishable with N samples if there exists no algorithm to reliably pass this binary hypothesis test.

Definition 2. *[Statistical indistinguishability (of distributions)] Two probability distributions $\mathcal{P}$ and $\mathcal{Q}$ are statistically indistinguishable with N samples if a binary hypothesis test cannot be passed with probability at least 0.51. That is, given a set of N samples $\mathcal{M}$ drawn from either $\mathcal{P}$ or $\mathcal{Q}$ (with an equal probability), consider the following hypotheses*

- *Null hypothesis $\mathcal{H}_0$: $\mathcal{M}$ is drawn from $\mathcal{P}$,*
- *Alternative hypothesis $\mathcal{H}_1$: $\mathcal{M}$ is drawn from $\mathcal{Q}$,*

where $\mathcal{P}$ and $\mathcal{Q}$ are statistically indistinguishable (with N samples) if for any algorithm the probability of correctly identifying the correct hypothesis, $\Pr[\text{“right decision between } \mathcal{H}_0 \text{ and } \mathcal{H}_1\text{”}]$, *satisfies:*

$$\Pr[\text{“right decision between } \mathcal{H}_0 \text{ and } \mathcal{H}_1\text{”}] \leqslant 0.51 \,. \tag{48}$$

Note that the threshold 0.51 in the definition is arbitrary chosen to be close to that of random guessing. We refer the reader to Supplementary Note II for a recap of basic results on hypothesis testing that we will use in the following.

We now recall that the exact kernel value between two given input vectors $\boldsymbol{x}$ and $\boldsymbol{x'}$ is fixed and not random. However, when $\kappa^{\mathrm{FQ}}(\boldsymbol{x}, \boldsymbol{x'})$ concentrates towards an exponentially small value μ, over the uniform distribution of input data pairs this kernel value is exponentially likely to be close to μ. That is, the exact kernel value is exponentially likely to be exponentially small.

In practice (for moderate to large-scale problems, i.e., for the problems we are ultimately interested in using quantum kernels for) we are limited to N samples where N scales polynomially with problem size. In this case, the distribution associated with the true kernel value, $\mathcal{P}_{\kappa^{\mathrm{FQ}}(\boldsymbol{x},\boldsymbol{x'})}$, and the uniform binary distribution, $\mathcal{P}_0$, are statistically indistinguishable. This argument is illustrated in Fig. 3 and formalized in the following Supplemental Lemma.

Supplemental Lemma 3. *Suppose that the fidelity quantum kernel $\kappa^{\mathrm{FQ}}(\boldsymbol{x}, \boldsymbol{x'})$ is exponentially concentrated over input data $\boldsymbol{x}$ and $\boldsymbol{x'}$ to some exponentially small value μ according to Definition 1. For any given $\boldsymbol{x}$ and $\boldsymbol{x'}$, we consider measuring $\kappa^{\mathrm{FQ}}(\boldsymbol{x}, \boldsymbol{x'})$ using a SWAP test, that is, samples are drawn from the distribution $\mathcal{P}_{\kappa^{\mathrm{FQ}}(\boldsymbol{x},\boldsymbol{x'})}$ in Eq. (46). Let $\mathcal{M}_s$ denote a set of $N \in poly(n)$ samples drawn either from $\mathcal{P}_{\kappa^{\mathrm{FQ}}(\boldsymbol{x},\boldsymbol{x'})}$ or $\mathcal{P}_0$ (with equal probability). We then perform a hypothesis test with:*

- *Null hypothesis $\mathcal{H}_0$: $\mathcal{M}_s$ is drawn from the uniform distribution $\mathcal{P}_0$,*
- *Alternative hypothesis $\mathcal{H}_s$: $\mathcal{M}_s$ is drawn from $\mathcal{P}_{\kappa^{\mathrm{FQ}}(\boldsymbol{x},\boldsymbol{x'})}$.*

With probability at least $1 - \delta_\kappa$ over the pairs of input data $\boldsymbol{x}$ and $\boldsymbol{x'}$, we have that

$$\Pr\left(\text{“right decision between } \mathcal{H}_s \text{ and } \mathcal{H}_0\text{”}\right) \leqslant \frac{1}{2} + \epsilon \,, \tag{49}$$

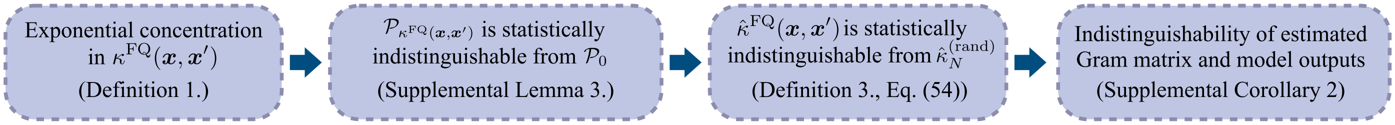

Supplementary Figure 3. **Summary of the impact of kernel concentration on the model outputs.** In the presence of kernel concentration, for any given input pair, its kernel value is highly likely to be exponentially close to a exponentially small value. This leads to the indistinguishability (with polynomial samples) between a distribution associated with a kernel $\mathcal{P}_{\kappa^{\mathrm{FQ}}(\boldsymbol{x},\boldsymbol{x'})}$ and a data-independent uniform distribution $\mathcal{P}_0$. Since the distributions themselves are indistinguishable, an estimate of kernel value (which is an empirical mean over samples) is also indistinguishable from an empirical mean over samples drawn from $\mathcal{P}_0$. Ultimately, a model trained on these kernel estimates behaves indistinguishably from an data-independent model.

with $\delta_\kappa \in \mathcal{O}(c^{-n})$ for some $c > 1$ and $\epsilon \in \mathcal{O}(c'^{-n})$ for some $c' > 1$. That is, with exponentially high probability over input data pairs $(\boldsymbol{x}, \boldsymbol{x'})$, the distributions $\mathcal{P}_0$ and $\mathcal{P}_{\kappa^{\mathrm{FQ}}(\boldsymbol{x},\boldsymbol{x'})}$ are statistically indistinguishable for large problem sizes with a polynomial number of samples N (as per Definition 2).

Proof. Our proof strategy is to show that, due to exponential concentration, the exact kernel value is very likely (i.e., with probability exponentially close to 1) to be exponentially small for any given input data pair $\boldsymbol{x}$ and $\boldsymbol{x'}$. This exponentially small kernel value corresponds to an exponentially small perturbation from the uniform distribution. Combining this observation with Supplemental Proposition 1 we can then establish that it is hard to decide the correct hypothesis with polynomial resources.

More explicitly, we first note that it follows from Supplemental Proposition 1 that

$$\Pr\left(\text{“right decision between } \mathcal{H}_s \text{ and } \mathcal{H}_0\text{”} \middle| \kappa^{\mathrm{FQ}}(\boldsymbol{x}, \boldsymbol{x'}) = s\right) \leqslant \left(\frac{1}{2} + \frac{Ns}{4}\right) . \tag{50}$$

For $s \in \mathcal{O}(c'^{-n})$ for some $c' > 1$ and $N \in \mathcal{O}(\mathrm{poly}(n))$ we have $\epsilon \in \mathcal{O}(c'^{-n})$ as claimed. It remains to determine with what probability we have $s \in \mathcal{O}(c'^{-n})$. By the assumption that the fidelity kernel concentrates to an exponentially small value over possible input data pairs (as per Definition 1), we have

$$\Pr_{\boldsymbol{x},\boldsymbol{x'}}\left[\left|\kappa^{\mathrm{FQ}}(\boldsymbol{x}, \boldsymbol{x'}) - \mu\right| \geqslant \delta_c\right] \leqslant \frac{\beta}{\delta_c^2} , \tag{51}$$

with

$$\beta \in \mathcal{O}(1/b^n) \;\;,\;\; \mu \in \mathcal{O}(1/b'^n) , \tag{52}$$

for some $b, b' > 1$. We then choose $\delta_c = \beta^{1/4}$ and invert the inequality of Eq. (51), leading to

$$\Pr_{\boldsymbol{x},\boldsymbol{x'}}\left[\left|\kappa^{\mathrm{FQ}}(\boldsymbol{x}, \boldsymbol{x'}) - \mu\right| \leqslant \beta^{1/4}\right] \geqslant 1 - \sqrt{\beta} . \tag{53}$$

It follows that $\kappa^{\mathrm{FQ}}(\boldsymbol{x}, \boldsymbol{x'})$ takes value between $\mu - \beta^{1/4}$ and $\mu + \beta^{1/4}$ (which are exponentially small) with probability at least $1 - \sqrt{\beta}$ (which is exponentially close to 1). Recalling the form $\mathcal{P}_{\kappa^{\mathrm{FQ}}(\boldsymbol{x},\boldsymbol{x'})}$ and $\mathcal{P}_0$ take in Eq. (46) and Eq. (47), we see that $\mathcal{P}_{\kappa^{\mathrm{FQ}}(\boldsymbol{x},\boldsymbol{x'})}$ is an exponentially small perturbation of $\mathcal{P}_0$ and the result follows by invoking Supplemental Proposition 1.

□

The central thesis of this section is that when fidelity kernels satisfy the conditions specified in Supplemental Lemma 3, they lead to useless models that do not generalize well. The argument is structured as follows. Due to the Representer Theorem, model outputs on unseen data are the output of some linear map on the statistical estimates obtained from experimental samples (which can be thought of as some post-processing). Thus, if we were able to take the model outputs and distinguish them from the outputs constructed from an (essentially useless) model based on the uniform distribution,

then we would succeed in the hypothesis test specified in Supplemental Lemma 3. Hence, by contradiction, it must not be possible to distinguish the model outputs constructed from such fidelity kernel values from those outputted from a model based on the uniform distribution. These models constructed from such fidelity kernels is then clearly useless. The last part of the argument is to observe that in an experimental setting, one has a strictly weaker setting than in the hypothesis test inr Supplemental Lemma 3, as one does not have access to the exact kernel values. Thus, the conclusion follows by reduction.

The above paragraph intuitively summarizes the consequences of exponential concentration on kernel-based quantum models. In what follows we present this argument in more detail. We start by defining a notion of indistinguishability for empirical outcomes sampled from distributions.

Definition 3 (Statistical indistinguishability (of outputs))**.** *Consider a map $\Phi : \mathbb{R}^N \to \mathbb{R}^M$ (with M being the dimension of the output) and two distributions $\mathcal{P}$ and $\mathcal{Q}$ which are statistically indistinguishable under N samples according to Definition 2. Draw N respective samples from $\mathcal{P}$ and $\mathcal{Q}$, which we respectively denote as $\mathcal{M}_{\mathcal{P}}$ and $\mathcal{M}_{\mathcal{Q}}$. We say that $\Phi(\mathcal{M}_{\mathcal{P}})$ and $\Phi(\mathcal{M}_{\mathcal{Q}})$ are statistically indistinguishable outputs.*

We introduce Definition 3 to describe the outputs and subsequent processing of samples drawn from indistinguishable distributions. Specifically, any distribution which satisfies Definition 2 automatically has outputs which satisfy Definition 3. In addition, as $\Phi(\mathcal{M}_{\mathcal{P}})$ and $\Phi(\mathcal{M}_{\mathcal{Q}})$ are constructed from samples of distributions, they themselves are random variables.

As an example, we would say that an experimentally obtained kernel value (an empirical mean) between any given input data $\boldsymbol{x}$ and $\boldsymbol{x'}$ estimated with a polynomial number of measurement outcomes/samples is statistically indistinguishable (with probability exponentially close to 1) from the empirical mean of the samples from the uniform distribution

$$\widehat{\kappa}_N^{(\mathrm{rand})} = \frac{1}{N}\sum_{m=1}^{N} \tilde{\lambda}_m \ , \tag{54}$$

where each $\tilde{\lambda}_m$ equally likely takes value $+1$ or -1.

Given a training dataset $\mathcal{S}$ of polynomial size N_s, consider the set of kernel values over possible pairs in $\mathcal{S}$ (excluding the trivial ones where $\kappa^{\mathrm{FQ}}(\boldsymbol{x},\boldsymbol{x}) = 1$)

$$\mathcal{K} = \left\{ \kappa^{\mathrm{FQ}}(\boldsymbol{x},\boldsymbol{x'}) \mid \forall \{\boldsymbol{x},\boldsymbol{x'}\} \subseteq \mathcal{S} \ ; \ \boldsymbol{x} \neq \boldsymbol{x'} \right\} \ . \tag{55}$$

Due to exponential concentration, each kernel value in this set is highly likely to be exponentially small and so Supplementary Lemma 3 will apply to each of these kernel values.

It then follows that any model computed by post-processing these samples is also, with high probability, statistically indistinguishable (as per Definition 3) from the model produced from the uniform binary distribution for each kernel entry. That is, the model predictions are independent of the input data and for all intents and purposes useless. This holds for any kernel method including both supervised and unsupervised learning tasks. For concreteness, let us again consider kernel ridge regression.

Supplemental Corollary 2. *[Full version of Proposition 2 and the SWAP part of Corollary 1 in the main text] Consider a kernel ridge regression task with the fidelity quantum kernel, a squared loss function and a training dataset $\mathcal{S} = \{\boldsymbol{x}_i, y_i\}_i$ of size $N_s \in \mathcal{O}(\mathrm{poly}(n))$. Given that the kernel value is estimated using the SWAP test and under the same assumptions as in Supplemental Lemma 3, the following statements hold with probability exponentially close to 1 (i.e., with the probability $1-\delta_a$ with $\delta_a \in \mathcal{O}(\tilde{c}^{-n})$ for $\tilde{c} > 1$)*

- *The estimated Gram matrix $\hat{K}$ is statistically indistinguishable (Def. 3) from an input-data-independent random matrix $\widehat{K}_N^{(\mathrm{rand})}$ whose diagonal elements are 1 and the off-diagonal elements are instances of $\widehat{\kappa}_N^{(\mathrm{rand})}$ in Eq. (54).*
- *The estimated optimal parameters are statistically indistinguishable (Def. 3) from the input-data-independent random variables*

$$\boldsymbol{a}_{\mathrm{rand}}(\boldsymbol{y},\lambda) = \left(\widehat{K}_N^{(\mathrm{rand})} - \lambda \mathbb{1}\right)^{-1} \boldsymbol{y} \, , \tag{56}$$

where $\widehat{K}_N^{(\mathrm{rand})}$ is a random matrix whose diagonal elements are 1 and off-diagonal elements are instances of $\widehat{\kappa}_N^{(\mathrm{rand})}$ in Eq. (54), $\boldsymbol{y}$ is a vector of output data points with its ith element equal to y_i and λ is the regularization parameter.

- *The model prediction on an unseen input data $\boldsymbol{x}$ is statistically indistinguishable (Def. 3) from the input-data-independent random variable*

$$h_{\mathrm{rand}} = \boldsymbol{a}_{\mathrm{rand}}(\boldsymbol{y},\lambda)^T \boldsymbol{k}_N^{(\mathrm{rand})} \ , \tag{57}$$

where $\boldsymbol{k}_N^{(\mathrm{rand})}$ is a random vector where each of its element is an instance of $\widehat{\kappa}_N^{(\mathrm{rand})}$.

Proof. We use Supplementary Lemma 3 with a union bound over the individual kernel values. Since $N_s \in \mathcal{O}(\mathrm{poly}(n))$, there are a polynomial number of kernel values to be estimated and each of the estimated kernel value is, with exponentially high probability, statistically indistinguishable to an instance of $\widehat{\kappa}_N^{(\mathrm{rand})}$.

More concretely, for the Gram matrix, we are required to estimate each kernel value in the kernel set $\mathcal{K}$ in Eq. (55) i.e., the off-diagonal elements. This amounts to $N_s(N_s-1)/2$ unique kernel values. Denote κ_i as an ith element in $\mathcal{K}$ with i running from 1 to $N_s(N_s-1)/2$. Let E_i be the event that the estimate of κ_i is statistically indistinguishable from $\widehat{\kappa}_N^{\mathrm{rand}}$. From Supplementary Lemma 3, we have

$$\Pr[E_i] \geqslant 1-\delta_\kappa \ \ , \forall \kappa_i \in \mathcal{K} \ , \tag{58}$$

with $\delta_\kappa \in \mathcal{O}(c^{-n})$ for $c>1$. Now, the probability that all E_i occur can be bounded as

$$\Pr\left[\bigcap_i E_i\right] = 1 - \Pr\left[\bigcup_i \bar{E}_i\right] \tag{59}$$

$$\geqslant 1 - \sum_{i=1}^{|\mathcal{K}|} \Pr\left[\bar{E}_i\right] \tag{60}$$

$$\geqslant 1 - \frac{N_s(N_s-1)\delta_k}{2} \ , \tag{61}$$

where $\bar{E}_i$ is a conjugate event of E_i, we use the union bound in the second line and use $\Pr[\bar{E}_i] \leqslant \delta_k$ by reversing the final inequality in Eq. (58). Since $N_s \in \mathcal{O}(\mathrm{poly}(n))$, we have that the probability that each of the kernel values are statistically indistinguishable (and so the Gram matrix is statistically indistinguishable) is $1-\delta_K$ with $\delta_K := N_s(N_s-1)\delta_k/2 \in \mathcal{O}(\tilde{c}^{-n})$ for some $\tilde{c}>1$.

The statistical indistinguishability of the optimal parameters directly follows from the above result. This is because estimating the optimal parameters is simply a post processing of the Gram matrix.

Lastly, to show indistinguishability of the model predictions, we have to take into account the kernel values for the test input. This requires estimating an additional N_s kernel values. On repeating the same argument using the union bound, it follows that the probability that all estimated kernel values (from the Gram matrix and new ones) are indistinguishable from $\widehat{\kappa}_N^{(\mathrm{rand})}$ is exponentially close to 1. □

In general a model that generalizes well must produce outputs that are data-dependent. Thus, these outputs must at minimum be distinguishable from a data-independent distribution. Hence, the models trained from these estimated Gram matrix have poor generalization. Lastly, similar to the Loschmidt Echo test, the training error can remain low as the correct output labels are effectively cooked into the model for the input data. Thus the model can "train" well. However, the trained model is insensitive to input data and thus poorly generalizes.

3. *Numerical simulation*

Fig. 4 demonstrates how kernel concentration affects the statistical estimates of kernel values via the Loschmidt Echo test (in panel (a)) and the SWAP test (in panel (b)) via a numerical example. We consider a training set where each

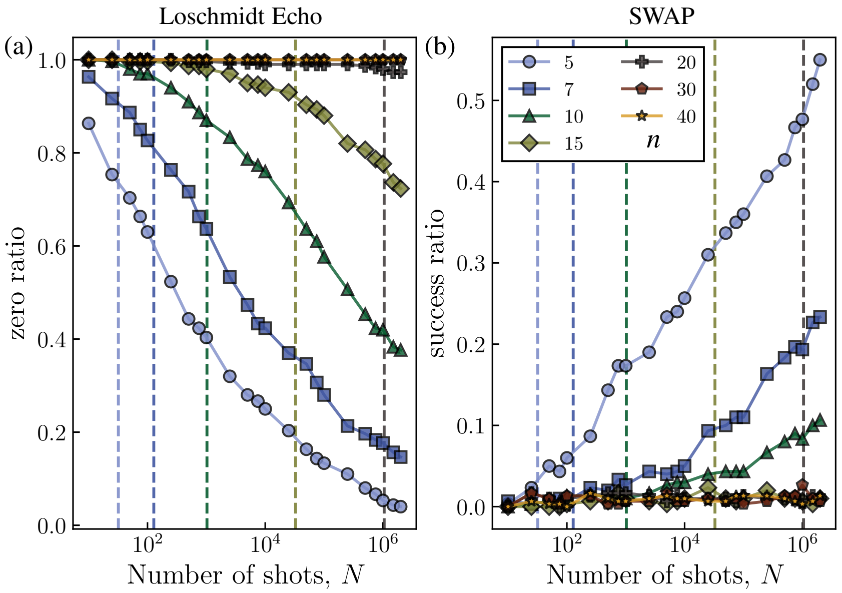

Supplementary Figure 4. **Effect of exponential concentration on estimated Gram matrix.** In panel (a), where the kernel values are estimated with the Loschmidt Echo test, we plot the zero ratio (i.e. the number of estimates that are zero compared to the total number of kernel values) with respect to the number of measurement shots used N, for different total number of qubits n. In panel (b), where the SWAP test is employed, we plot the success ratio (which is the number of estimates that pass the binomial test from the uniform distribution with p-value= 0.01 compared to the total number of kernel values). The x-axis indicates the number of shots used per kernel value and vertical lines indicate the dimension of the (exponentially increasing) Hilbert space 2^n. Here the training data size is $N_s = 25$.

input data point is a $n-$dimensional vector with each element uniformly drawn from $[0, 2\pi]$, and is encoded via a tensor product embedding which consists of a layer of single-qubit R_y rotation gates. In this setting, kernel values exponentially concentrate to an exponentially small value μ (see the sources of concentration section in the main text). Each unique off-diagonal element in the Gram matrix is evaluated with an increasing number of measurement shots (as indicated in the x-axis of both panels). Note that in our analysis we fix diagonal elements to be 1 without evaluation as one may do in realistic setting.

In panel (a) where the Loschmidt Echo test is employed, we plot the ratio of the number of statistical estimates that are zero to the total number of kernel values as a function of qubits and measurement shots. When the ratio is 1, this indicates that all kernel estimates are zero. In general, this fraction becomes smaller with increasing measurement shots. We also observe that in order to achieve a fixed ratio of non-zero values (e.g. ~ 0.75), exponentially many measurement shots are required i.e., $N \in \Omega(2^n)$. Particularly, at 30 and 40 qubits, all of the estimates are zero even with 2×10^6 shots per kernel value.

In panel (b) where the kernel values are estimated with SWAP tests, for each individual kernel value, we perform a binomial hypothesis test on the measurement outcomes to see whether or not there is sufficient statistical significance to distinguish the shots from those obtained from the uniform distribution. We plot the ratio of the estimates that pass the binomial test (with p-value below 0.01) as a function of qubits and measurement shots. A low ratio indicates that most of the estimates are statistically indistinguishable from the ones estimated with the data-independent uniform distribution. It can be seen from the panel that to maintain a constant success ratio the number of measurement shots needs to scale at least exponentially with the number of qubits.

B. Projected quantum kernel

As discussed in our main text, the projected quantum kernel is an alternative approach to comparing data-encoded quantum states. It takes the form

$$\kappa^{PQ}(\boldsymbol{x}, \boldsymbol{x'}) = \exp\left(-\gamma \sum_{k=1}^{n} \|\rho_k(\boldsymbol{x}) - \rho_k(\boldsymbol{x'})\|_2^2\right) , \tag{62}$$

where $\rho_k(\boldsymbol{x})$ is the reduced state of $\rho(\boldsymbol{x})$ on the k-th qubit, $\|\cdot\|_2$ is the Schatten 2-norm and γ is a positive hyperparameter.

Estimating the projected quantum kernel in practice requires us to first obtain statistical estimates of the 2-norms on all individual qubits and then classically post-process them to estimate the kernel value. Here we consider two common strategies to estimate the 2-norms.

Tomography strategy: First, we perform full state tomography on the reduced density matrices. As these are single-qubit states the number of required measurements is constant with respect to the number of qubits. In particular, the reduced state to the kth qubit can be expressed in the Pauli basis as

$$\rho_k(\boldsymbol{x}) = \frac{1}{2}\left(\mathbb{1}_k + c_{x_k}(\boldsymbol{x})X_k + c_{y_k}(\boldsymbol{x})Y_k + c_{z_k}(\boldsymbol{x})Z_k\right) , \tag{63}$$

where $\{X_k, Y_k, Z_k\}$ are single X, Y and Z Pauli matrices on the qubit k with corresponding coefficients $\{c_{x_k}(\boldsymbol{x}), c_{y_k}(\boldsymbol{x}), c_{z_k}(\boldsymbol{x})\}$. Each coefficient is simply the expectation value with the respective Pauli observable

$$c_{\sigma_k}(\boldsymbol{x}) = \text{Tr}[\rho_k(\boldsymbol{x})\sigma_k] , \tag{64}$$

with $\sigma_k \in \{X_k, Y_k, Z_k\}$. To estimate each of the $3n$ coefficients, we can make local measurements in each respective basis. The measurement outcome is either $+1$ with probability $p_+ = 1/2 + c_{\sigma_k}(\boldsymbol{x})/2$ and -1 with probability $1 - p_+$. That is, we have the distribution

$$\mathcal{P}_{\sigma_k, \boldsymbol{x}} = \left\{\frac{1 + c_{\sigma_k}(\boldsymbol{x})}{2}, \frac{1 - c_{\sigma_k}(\boldsymbol{x})}{2}\right\} . \tag{65}$$

After some specified N measurement samples, the statistical estimate of the expectation value is obtained via taking their empirical mean in the usual way. After an estimate of each reduced density matrix is obtained for all qubits and data values, an estimate of the kernel values in Eq. (62) can be evaluated via matrix algebra.

Local SWAP strategy: Alternatively, we can employ local SWAP tests to evaluate the 2-norms. In particular, by explicitly expanding the 2-norm, we have

$$\|\rho_k(\boldsymbol{x}) - \rho_k(\boldsymbol{x'})\|_2^2 = \text{Tr}\left[\rho_k^2(\boldsymbol{x})\right] + \text{Tr}\left[\rho_k^2(\boldsymbol{x'})\right] - 2\,\text{Tr}[\rho_k(\boldsymbol{x})\rho_k(\boldsymbol{x'})] . \tag{66}$$

That is, the 2-norm distance contains the overlap between two reduced states $\text{Tr}[\rho_k(\boldsymbol{x})\rho_k(\boldsymbol{x'})]$ and the purity of each individual reduced state $\text{Tr}\left[\rho_k^2(\boldsymbol{x})\right]$, $\text{Tr}\left[\rho_k^2(\boldsymbol{x'})\right]$. Each term in Eq. (66) can be estimated using the local SWAP test. Similar to the fidelity case previously, each term in the 2-norm is equal to the expectation value of the Pauli-Z operator on an ancilla qubit where each measurement gives either $+1$ or -1. More precisely, denote $m_k(\boldsymbol{x}, \boldsymbol{x'}) = \text{Tr}[\rho_k(\boldsymbol{x})\rho_k(\boldsymbol{x'})]$. When making measurements, an individual outcome takes $+1$ with probability $p_+ = 1/2 + m_k(\boldsymbol{x}, \boldsymbol{x'})/2$ and -1 with $p_+ = 1 - p_-$ i.e. we sample from the distribution

$$\mathcal{P}_{m_k(\boldsymbol{x}, \boldsymbol{x'})} = \left\{\frac{1 + m_k(\boldsymbol{x}, \boldsymbol{x'})}{2}, \frac{1 - m_k(\boldsymbol{x}, \boldsymbol{x'})}{2}\right\} . \tag{67}$$

Then, the statistical estimate of $m_k(\boldsymbol{x}, \boldsymbol{x'})$ can be obtained as an empirical mean of these outcomes.

1. Consequence of exponential concentration

To see how concentration affects the projected quantum kernel in practice, we take as our starting point the assumption that

$$\mathbb{E}_{\boldsymbol{x}\in\mathcal{X}} \left\| \rho_k(\boldsymbol{x}) - \frac{\mathbb{1}_k}{2} \right\|_2 \leqslant \beta \in \mathcal{O}\left(\frac{1}{b^n}\right) \ , \tag{68}$$

for all $k \in \{1, ..., n\}$, where the expectation value is taken over some chosen distribution over $\mathcal{X}$. In the later sections, we show that all sources of the exponential concentration in the projected quantum kernel also lead to this exponential vanishing of the 2-norm distance between the reduced quantum states. The following lemma shows the connection between this reduced state concentration and kernel concentration.

Supplemental Lemma 4. *Given that the Eq. (68) is satisfied, it follows that the projected quantum kernel exponentially concentrates*

$$\Pr_{\boldsymbol{x},\boldsymbol{x'}\in\mathcal{X}} \left[\left| \kappa^{PQ}(\boldsymbol{x},\boldsymbol{x'}) - \mu \right| \geqslant \delta \right] \leqslant \frac{\beta}{\delta^2} \ , \tag{69}$$

where $\beta \in \mathcal{O}(1/b^n)$ for some $b > 1$.

We defer the proof to Supplementary Note III B 3.

We now show that if the distance of the reduced state from the maximally mixed state is exponentially small, i.e. Eq. (68) holds, then for both tomography and local SWAP strategies, the (data-dependent) distribution associated with each quantity of interest is statistically indistinguishable from a fixed distribution, as per Definition 2.

Supplemental Lemma 5. *Assume the 2-norm distance between the reduced data encoding states exponentially vanishes as in Eq. (68). Consider the following two scenarios*

1. *For the tomography strategy, suppose we measure any coefficient $c_{\sigma_k(\boldsymbol{x})}$ of a reduced state on the qubit k in Eq. (64) for a given input data $\boldsymbol{x}$ with a polynomial number of measurement shots. The associated distribution $\mathcal{P}_{\sigma_k,\boldsymbol{x}}$ defined in Eq. (65) is statistically indistinguishable (as per Definition 2) from the data independent uniform distribution $\mathcal{P}_0 = \{1/2, 1/2\}$ in Eq. (47) (with the probability exponentially close to 1).*

2. *For the local SWAP strategy, suppose we measure any one of the terms $m_k(\boldsymbol{x},\boldsymbol{x'})$ in Eq. (66) for a given input data pair $\boldsymbol{x}$ and $\boldsymbol{x'}$ with a polynomial number of measurement shots. The associated distribution $\mathcal{P}_{m_k(\boldsymbol{x},\boldsymbol{x'})}$ is statistically indistinguishable from a data-independent fixed distribution $\tilde{\mathcal{P}}_0 = \{3/4, 1/4\}$ (with the probability exponentially close to 1).*

Again, we provide the proof in Supplementary Note III B 3.

Supplemental Lemma 5 plays the same pivotal role for the projected kernel as Supplemental Lemma 3 for the fidelity kernel. They both capture the statistical indistinguishability of the distributions obtained with quantum computers when performing an experiment. Therefore, an identical reasoning can be applied here, that is, we argue that any trained model built frompolynomial samples is insensitive to input data and thus poorly generalizes. Similar to before, we note that estimating projected kernel values (via Eq. (62)) and model predictions (via the Representer Theorem) can be seen as forms of post processing measurement outcomes. If these model predictions can be distinguished from the model predictions constructed based on the fixed distribution, then the hypothesis task described in Supplemental Lemma 5 would be succeeded and hence contradict the conclusion of the lemma. Finally, we note that the setting in Supplemental Lemma 5 is strictly stronger than what we have in practice where there is no access to exact quantities of interest (i.e., coefficients in the tomography strategy and purity/overlap terms in the local SWAP strategy) and in turn exact kernel values.

In the following, we formalize the above. We will again use our notion of statistical indistinguishability out outputs from Definition 3. Following Supplemental Lemma 5, we will observe the following quantities to be indistinguishable:

- For the tomography strategy, the coefficient $c_{\sigma_k}(\boldsymbol{x})$ in Eq. (64) estimated with polynomial samples is statistically indistinguishable from $\widehat{\kappa}_N^{(\mathrm{rand})}$ in Eq. (54).
- For the local SWAP strategy, the statistical estimate of the purity/overlap term is indistinguishable from another data-independent random variable i.e.,

$$\widehat{\kappa}_N^{(\text{biased rand})} = \frac{1}{N}\sum_{m=1}^{N}\tilde{\lambda}_m \ , \tag{70}$$

where $\tilde{\lambda}_m$ takes $+1$ value with probability $3/4$ and -1 value with probability $1/4$.

To obtain an estimate of a 2-norm distance on qubit k, one has to estimate either all coefficients for the reduced states in Eq. (63) for the tomography strategy, or all purity/overlap terms Eq. (65) for the local SWAP strategy. Each term follows Supplemental Lemma 5. This leads to the statistical indistinguishability between the estimated 2-norm on the qubit k and some data-independent random variable (with high probability).

To construct an estimate for a kernel value one requires statistical estimates of the 2-norms associated with all single qubit subsystems and then one sums them as in Eq. (62). In the following Supplemental Proposition 3 we argue these kernel values are indistinguishable.

Supplemental Proposition 3. *Consider the same assumption as in Supplemental Lemma 5 and a polynomial number of measurement shots used to estimate the 2-norms. For any input data pair $\boldsymbol{x}$ and $\boldsymbol{x'}$, we have the following, with the probability exponentially close to 1 (i.e., with the probability $1-\delta_a$ with $\delta_a \in \mathcal{O}(\tilde{c}^{-n})$ for $\tilde{c} > 1$)*

- *For the tomography strategy, the statistical estimate of the 2-norm between two reduced quantum states is statistically indistinguishable (as per Defintion 3) from an input-data-independent random variable*

$$\ell_N^{(\mathrm{rand,T})} = \frac{1}{2}\left[\left(\widehat{\kappa}_{N,1}^{(\mathrm{rand})} - \widehat{\kappa}_{N,2}^{(\mathrm{rand})}\right)^2 + \left(\widehat{\kappa}_{N,3}^{(\mathrm{rand})} - \widehat{\kappa}_{N,4}^{(\mathrm{rand})}\right)^2 + \left(\widehat{\kappa}_{N,5}^{(\mathrm{rand})} - \widehat{\kappa}_{N,6}^{(\mathrm{rand})}\right)^2\right] \ , \tag{71}$$

where $\left\{\widehat{\kappa}_{N,i}^{(\mathrm{rand})}\right\}_{i=1}^{6}$ are different instances of $\widehat{\kappa}_N^{(\mathrm{rand})}$ defined in Eq. (54).

- *For the SWAP strategy, the statistical estimate of the 2-norm between two reduced quantum states is statistically indistinguishable from an input-data-independent random variable*

$$\ell_N^{(\mathrm{rand,S})} = \widehat{\kappa}_{N,1}^{(\text{biased rand})} + \widehat{\kappa}_{N,2}^{(\text{biased rand})} - 2\widehat{\kappa}_{N,3}^{(\text{biased rand})} \ , \tag{72}$$

where $\left\{\widehat{\kappa}_{N,i}^{(\text{biased rand})}\right\}_{i=1}^{3}$ are different instances of $\widehat{\kappa}_N^{(\text{biased rand})}$ as defined in Eq. (70).

In addition, the estimate of the projected quantum kernel is statistically indistinguishable from an input-data-independent random variable

$$\widehat{\kappa}_N^{(\mathrm{rand,PQ})} = \exp\left[\gamma\sum_{k=1}^{n}\ell_{N,k}^{(\mathrm{rand})}\right] \ , \tag{73}$$

where $\left\{\ell_{N,k}^{(\mathrm{rand})}\right\}_{k=1}^{n}$ are different instances of either $\ell_N^{(\mathrm{rand,T})}$ for the tomography strategy, or $\ell_N^{(\mathrm{rand,S})}$ for the SWAP strategy.

The proof is detailed in Supplementary Note III B 3.

Identical to the fidelity kernel setting, we now argue that the statistical indistinguishability between estimated projected kernel value and some data-independent random variable leads to estimated model predictions that are insensitive to unseen input data (with high probability). To proceed, consider a training dataset $\mathcal{S}$ of polynomial size N_s which

corresponds to a set that contains projected kernel values over possible training data pairs (excluding the trivial ones $\kappa^{\rm PQ}(\boldsymbol{x},\boldsymbol{x})=1$).

$$\mathcal{K}_{\rm PQ} = \left\{ \kappa^{\rm PQ}(\boldsymbol{x},\boldsymbol{x'}) \mid \forall \boldsymbol{x},\boldsymbol{x'} \in \mathcal{S} \; ; \; \boldsymbol{x} \neq \boldsymbol{x'} \right\} \; . \tag{74}$$

Supplemental Proposition 3 applies for each kernel value in $\mathcal{K}_{\rm PQ}$, leading to its estimated kernel being indistinguishable with probability exponentially close to 1. Since the cardinality of $\mathcal{K}_{\rm PQ}$ is at most polynomial in the number of qubits, it follows that the probability of all estimated kernel values being indistinguishable remains exponentially close to 1. This leads to the indistinguishability of the estimated Gram matrix from some data-independent random matrix. This precludes the usefulness of the rest of the kernel methods pipeline. Again we demonstrate this for the task of kernel ridge regression and provide the form of an input-data-independent random variable that the model prediction approximately takes.

Supplemental Corollary 3. *Consider a kernel ridge regression task with the projected quantum kernel, a squared loss function and a training dataset $\mathcal{S}=\{\boldsymbol{x},y_i\}_i$ with $N_s \in \mathcal{O}(\mathrm{poly}(n))$. Given that the kernel value is estimated using either the SWAP test or tomography strategies and under the same assumption as in Supplemental Lemma 5, the following statements hold with probability exponentially close to 1 (i.e., with the probability $1-\delta_a$ with $\delta_a \in \mathcal{O}(\tilde{c}^{-n})$ for $\tilde{c}>1$)*

- *The estimated Gram matrix $\widehat{K}$ is statistically indistinguishable (as per Definition 3) from an input-data-independent matrix $\widehat{K}_N^{(\rm rand,PQ)}$ whose diagonal elements are 1 and off-diagonal elements are instances of $\widehat{\kappa}_N^{(\rm rand,PQ)}$ determined by Supplemental Proposition 3.*

- *The estimated optimal parameters are statistically indistinguishable from the input-data-independent random variables*

$$\boldsymbol{a}_{\rm rand}(\boldsymbol{y},\lambda) = \left(\widehat{K}_N^{(\rm rand,PQ)} - \lambda \mathbb{1}\right)^{-1} \boldsymbol{y} \; . \tag{75}$$

  where $\boldsymbol{y}$ is a vector of output data points with its $i^{\rm th}$ element equal to y_i and λ is the regularization parameter.

- *The model prediction on an unseen input data $\boldsymbol{x}$ is statistically indistinguishable from the input data independent random variable*

$$h_{\rm rand} = \boldsymbol{a}_{\rm rand}(\boldsymbol{y},\lambda)^T \boldsymbol{k}_N^{(\rm rand,PQ)} \; , \tag{76}$$

  where $\boldsymbol{k}_N^{(\rm rand,PQ)}$ is a random vector where each of its element is an instance of $\widehat{\kappa}_N^{(\rm rand,PQ)}$ in Eq. (73).

We refer the reader to Supplementary Note III B 3 for the proof of the corollary.

Again, as in the case of the quantum fidelity kernel, the training process hard encodes the training label which makes it possible to have a small training error. However, crucially the model does not obtain any information about the input data both during the training and the prediction phases which results in very poor generalization.

2. *Numerical simulation*

In Fig. 5, we numerically study the consequences of exponential concentration on the indistinguishability of statistical estimates for projected quantum kernels. Here, we consider a training dataset of size $N_s=25$ and the data embedding is chosen such that it maps a classical input to a maximally expressive state, which leads to the exponential concentration of quantum states and in turn the projected quantum kernel (see the sources of concentration section in the main text). We perform binomial hypothesis tests on statistical estimates of the data-dependent quantities as though they were obtained from a quantum experiment to see whether or not they are statistically significant enough to be distinguishable from the associated fixed distributions. More specifically, in panel (a), local SWAP tests are employed to measure purities and overlaps of reduced states, we plot the success ratio of the estimates that pass the binomial test (with p-value below 0.01) to the total number of estimates. Similarly, panel (b) illustrates this success ratio when the tomography is used

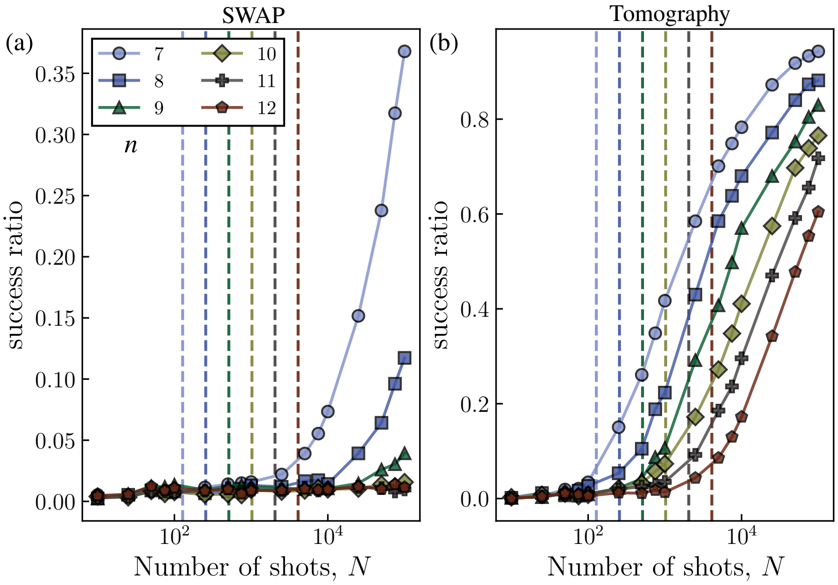

Supplementary Figure 5. **Effect of exponential concentration on the estimated Gram matrix for the projected kernel.** We plot the success ratio, i.e. the number of estimates of relevant quantities that pass a binomial test from their respective fixed distribution for a p-value= 0.01 to the total number of estimates as a function of measurement shots N and qubits n. Two strategies for preparing the projected quantum kernel are considered, as discussed at the start of this section. In panel (a) we consider the SWAP test, where the relevant experimental quantities are the overlaps and purities of reduced data encoded states. In panel (b), where we consider the tomography strategy, the relevant quantities are the Pauli coefficients of reduced data encoded states. The x-axis indicates the number of shots used per kernel value, and vertical lines indicate the (exponentially increasing) dimension of the Hilbert space 2^n. Here the training data set is of size $N_s = 25$.

to estimate coefficients of reduced states. In either case, we can see that to obtain a fixed success ratio an exponential number of measurement shots are required to have a significant increase in the ratio. Consequently, for larger system sizes, where only polynomial number of shots is feasible, statistical estimates of these relevant quantities are indistinguishable from the data-independent random variables.

More practical matters of trainability and generalizability are numerically studied in Fig. 6 for a 12-qubit simulation. Here, we consider a training dataset of size $N_s = 200$ where each individual input data is mapped to a maximally expressive state leading to exponential concentration. A true label is constructed in a similar manner as in Fig. 3 in the main text which gives perfect generalization when training on the whole dataset with exact kernel values. In the main plot, we study the generalization performance when a fraction of the dataset is used to train the model. With direct access to exact kernel values, the model generalizes better with increasing data, as expected. On the other hand, when kernel values are estimated with either the SWAP or tomography strategy and limited measurement samples ($N = 1000$), generalization does not get better with increasing training data. In addition, the behavior of the *relative* error also closely follows a similar trend to when the model is trained on a random matrix as its Gram matrix. Lastly, we observe perfect training errors in all cases. We posit this is again because the optimization process directly encodes the information about training labels.

3. *Proof of analytical results*

Here we provide the proofs of the main analytical results regarding the practical consequence of exponential concentration on the projected quantum kernel as stated in Subsection III B 1. For the readers' convenience, we restate formal statements before detailing proofs.

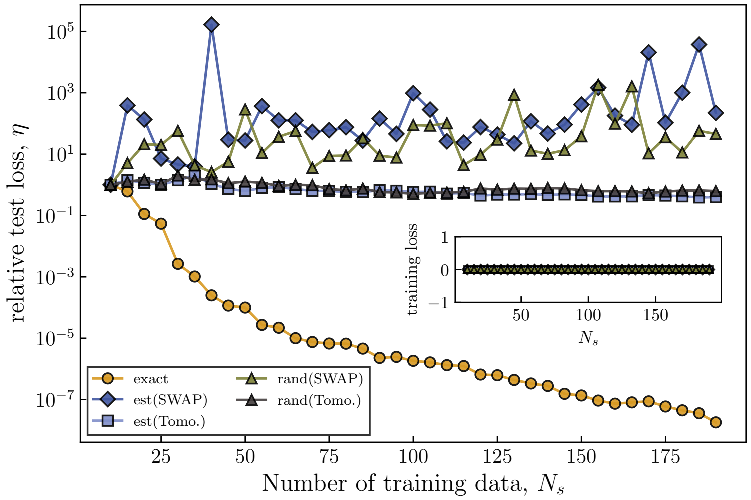

Supplementary Figure 6. **Effect of exponential concentration on training and generalization performance for projected quantum kernels.** In the main plot, a *relative* loss on a test dataset $\mathcal{S}_{\text{test}}$ with respect to its initial value is plotted as a function of increasing training data and how the kernel values are obtained. In the inset, an *absolute* training error is plotted as a function of increasing data. We note that each kernel value is estimated with $N = 1000$ by either SWAP or tomography strategies, and the number of testing data points is 20. The training is carried out with no regularization $\lambda = 0$.

Supplemental Lemma 4. *Given that the Eq. (68) is satisfied, it follows that the projected quantum kernel exponentially concentrates*

$$\Pr_{\boldsymbol{x},\boldsymbol{x'}\in\mathcal{X}}\left[\left|\kappa^{PQ}(\boldsymbol{x},\boldsymbol{x'})-\mu\right|\geqslant\delta\right]\leqslant\frac{\beta}{\delta^2}\,, \tag{77}$$

where $\beta \in \mathcal{O}(1/b^n)$ for some $b > 1$.

Proof. We first show that the variance of the projected kernel is exponentially small due to the state concentration in

Eq. (68). The variance of the projected kernel can be bounded

$$\begin{aligned}
\mathrm{Var}_{\boldsymbol{x},\boldsymbol{x'}}[k^{PQ}(\boldsymbol{x},\boldsymbol{x'})] &= \mathrm{Var}_{\boldsymbol{x},\boldsymbol{x'}}[1-k^{PQ}(\boldsymbol{x},\boldsymbol{x'})] &(78)\\
&\leqslant \mathbb{E}_{\boldsymbol{x},\boldsymbol{x'}}[(1-k^{PQ}(\boldsymbol{x},\boldsymbol{x'}))^2] &(79)\\
&\leqslant \mathbb{E}_{\boldsymbol{x},\boldsymbol{x'}}[1-k^{PQ}(\boldsymbol{x},\boldsymbol{x'})] &(80)\\
&= \mathbb{E}_{\boldsymbol{x},\boldsymbol{x'}}\left[1-e^{-\gamma\sum_{k=1}^n \|\rho_k(\boldsymbol{x})-\rho_k(\boldsymbol{x'})\|_2^2}\right] &(81)\\
&\leqslant \mathbb{E}_{\boldsymbol{x},\boldsymbol{x'}}\left[\gamma\sum_{k=1}^n \|\rho_k(\boldsymbol{x})-\rho_k(\boldsymbol{x'})\|_2^2\right] &(82)\\
&= \gamma\sum_{k=1}^n \mathbb{E}_{\boldsymbol{x},\boldsymbol{x'}}\|\rho_k(\boldsymbol{x})-\rho_k(\boldsymbol{x'})\|_2^2\,, &(83)\\
&\leqslant \gamma\sum_{k=1}^n \mathbb{E}_{\boldsymbol{x},\boldsymbol{x'}}\left(\left\|\rho_k(\boldsymbol{x})-\frac{\mathbb{1}_k}{2}\right\|_2+\left\|\rho_k(\boldsymbol{x'})-\frac{\mathbb{1}_k}{2}\right\|_2\right)^2 &(84)\\
&\leqslant 2\gamma\sum_{k=1}^n \left(\mathbb{E}_{\boldsymbol{x}}\left\|\rho_k(\boldsymbol{x})-\frac{\mathbb{1}_k}{2}\right\|_2+\mathbb{E}_{\boldsymbol{x'}}\left\|\rho_k(\boldsymbol{x'})-\frac{\mathbb{1}_k}{2}\right\|_2\right) &(85)\\
&\in \mathcal{O}\left(\frac{n}{b^n}\right)\,, &(86)
\end{aligned}$$

where the first equality is due to the fact that $\mathrm{Var}_{\boldsymbol{\alpha}}[c_1A(\boldsymbol{\alpha})+c_2] = c_1^2\mathrm{Var}_{\boldsymbol{\alpha}}[A(\boldsymbol{\alpha})]$ for constants c_1 and c_2, in the second inequality we use the bound $(1-k^{PQ}(\boldsymbol{x},\boldsymbol{x'}))\leqslant 1$ and take the upper bound, the second equality follows from substituting in the kernel definition in Eq. (62) and in the third inequality we use $1-e^{-t}\leqslant t$. Then, in the fourth inequality, we denote $\mathbb{1}_k$ as the identity matrix on qubit k and use the triangle inequality. The fifth inequality is from $(t+s)^2\leqslant 2t^2+2s^2$ and in the last line the state concentration in Eq. (68) is used. Finally, the kernel concentration, Eq. (69), follows directly from Chebyshev's inequality. □

Supplemental Lemma 5. *Assume the 2-norm distance between the reduced data encoding states exponentially vanishes as in Eq. (68). Consider the following two scenarios*

1. *For the tomography strategy, suppose we measure any coefficient $c_{\sigma_k(\boldsymbol{x})}$ of a reduced state on the qubit k in Eq. (64) for a given input data $\boldsymbol{x}$ with a polynomial number of measurement shots. The associated distribution $\mathcal{P}_{\sigma_k,\boldsymbol{x}}$ defined in Eq. (65) is statistically indistinguishable (as per Definition 2) from the data independent uniform distribution $\mathcal{P}_0=\{1/2,1/2\}$ in Eq. (47) (with the probability exponentially close to 1).*

2. *For the local SWAP strategy, suppose we measure any one of the terms $m_k(\boldsymbol{x},\boldsymbol{x'})$ in Eq. (66) for a given input data pair $\boldsymbol{x}$ and $\boldsymbol{x'}$ with a polynomial number of measurement shots. The associated distribution $\mathcal{P}_{m_k(\boldsymbol{x},\boldsymbol{x'})}$ is statistically indistinguishable from a data-independent fixed distribution $\tilde{\mathcal{P}}_0=\{3/4,1/4\}$ (with probability exponentially close to 1).*

Proof. To prove our result, we first consider a fixed distribution $\mathcal{P}=\{p,1-p\}$ and some perturbed distribution $\mathcal{P}_\varepsilon=\{p+\varepsilon,1-(p+\varepsilon)\}$. Recall from Supplemental Proposition 1 that the perturbation ε plays a crucial role in the success probability of the hypothesis test (with N samples)

$$\Pr\left(\text{“right decision between } \mathcal{H}_0 \text{ and } \mathcal{H}_1\text{”}\right)\leqslant\left(\frac{1}{2}+\frac{N\varepsilon}{4}\right)\,. \tag{87}$$

Then, if the perturbation becomes exponentially small, $\mathcal{P}$ and $\mathcal{P}_\epsilon$ are statistically indistinguishable with the polynomial samples $N\in\mathcal{O}(\mathrm{poly}(n))$ for large n by Definition 2.

Our proof for each of the scenarios mainly consists of using the exponential concentration of the 2-norm in Eq. (68) (i) to show that for a given input the quantity that we are interested in measuring is exponentially close to some fixed data-independent value (with high probability) and (ii) to identify the relevant fixed distribution (in case of the tomography strategy, this fixed distribution is $\mathcal{P}_0 = \{1/2, 1/2\}$, while in the case of the local SWAP test we have $\tilde{\mathcal{P}_0} = \{3/4, 1/4\}$). Together, this establishes statistical indistinguishability between the distribution associated with the quantity and the corresponding fixed distribution.

Tomography strategy: The quantity of interest is the expectation value of a Pauli observable on the qubit k i.e., $c_{\sigma_k,\boldsymbol{x}}$. We now show that $c_{\sigma_k,\boldsymbol{x}}$ is exponentially concentrated by looking at its variance.

$$\begin{aligned}
\mathrm{Var}_{\boldsymbol{x}}\left[c_{\sigma_k,\boldsymbol{x}}\right] &= \mathrm{Var}_{\boldsymbol{x}}\left[\mathrm{Tr}[\rho_k(\boldsymbol{x})\sigma_k]\right] && (88)\\
&= \mathrm{Var}_{\boldsymbol{x}}\left[\mathrm{Tr}\left[\left(\rho_k(\boldsymbol{x}) - \frac{\mathbb{1}_k}{2}\right)\sigma_k\right]\right] && (89)\\
&\leqslant \mathbb{E}_{\boldsymbol{x}}\left[\mathrm{Tr}\left[\left(\rho_k(\boldsymbol{x}) - \frac{\mathbb{1}_k}{2}\right)\sigma_k\right]\right]^2 && (90)\\
&\leqslant \mathbb{E}_{\boldsymbol{x}}\left[\left\|\rho_k(\boldsymbol{x}) - \frac{\mathbb{1}_k}{2}\right\|_2^2 \|\sigma_k\|_2^2\right] && (91)\\
&\leqslant \sqrt{2}\,\mathbb{E}_{\boldsymbol{x}}\left\|\rho_k(\boldsymbol{x}) - \frac{\mathbb{1}_k}{2}\right\|_2 && (92)\\
&\in \mathcal{O}\left(\frac{1}{b^n}\right), && (93)
\end{aligned}$$

where the second equality is due to the Pauli operator being traceless, the second inequality is from using Hölder's inequality and in the third inequality we use the fact that $\|\sigma_k\|_2^2 = 2$ as well as $\|\rho_k(\boldsymbol{x}) - \mathbb{1}_k/2\|_1 \leqslant 1/\sqrt{2}$. The final line follows from the assumption that the 2-norm distance between the single qubit reduced data-encoded states and the maximally mixed state vanishes as per Eq. (68). This shows that the exponential concentration of the variance towards its mean. The mean can be further shown to exponentially vanish as follows

$$\begin{aligned}
\mathbb{E}_{\boldsymbol{x}}\left[c_{\sigma_k,\boldsymbol{x}}\right] &= \mathbb{E}_{\boldsymbol{x}}\left[\mathrm{Tr}\left[\left(\rho_k(\boldsymbol{x}) - \frac{\mathbb{1}_k}{2}\right)\sigma_k\right]\right] && (94)\\
&\leqslant \mathbb{E}_{\boldsymbol{x}}\left[\left\|\rho_k(\boldsymbol{x}) - \frac{\mathbb{1}_k}{2}\right\|_2 \|\sigma_k\|_2\right] && (95)\\
&\in \mathcal{O}\left(\frac{1}{b^n}\right), && (96)
\end{aligned}$$

where the equality is due to the Pauli operator being traceless and the inequality is from using Hölder's inequality. Together, we have that the coefficient exponentially concentrates towards some exponentially small value.

By applying the Chebyshev's inequality, it can be shown that for any given $\boldsymbol{x}$ the coefficient is exponentially small with high probability. That is, by denoting $\mu = \mathbb{E}_{\boldsymbol{x}}\left[c_{\sigma_k,\boldsymbol{x}}\right]$ and $\sigma^2 = \mathrm{Var}_{\boldsymbol{x}}\left[c_{\sigma_k,\boldsymbol{x}}\right]$, we have

$$\mathrm{Pr}_{\boldsymbol{x}}\left[|c_{\sigma_k,\boldsymbol{x}} - \mu| \geqslant k\sigma\right] \leqslant \frac{1}{k^2}\,. \tag{97}$$

Then by choosing $k = 1/\sqrt{\sigma}$ and inverting the inequality of Eq. (51), this gives us

$$\mathrm{Pr}_{\boldsymbol{x}}\left[|c_{\sigma_k,\boldsymbol{x}} - \mu| \leqslant \sqrt{\sigma}\right] \geqslant 1 - \sigma\,. \tag{98}$$

Therefore, $c_{\sigma_k,\boldsymbol{x}}$ takes a value between $\mu - \sqrt{\sigma}$ and $\mu + \sqrt{\sigma}$ (which are exponentially small) with the probability at least $1 - \sigma$ (which is exponentially close to 1). Lastly, the fixed distribution can be identified by replacing $c_{\sigma_k,\boldsymbol{x}}$ in $\mathcal{P}_{\sigma_k,\boldsymbol{x}}$ with 0 (i.e., no perturbation) leading to $\mathcal{P}_0 = \{1/2, 1/2\}$. This finishes the first part of the proof.

SWAP strategy: Here we are interested in estimating $m_k(\boldsymbol{x}, \boldsymbol{x'}) = \mathrm{Tr}[\rho_k(\boldsymbol{x})\rho_k(\boldsymbol{x'})]$ which corresponds to the purity if $\boldsymbol{x} = \boldsymbol{x'}$ and to the overlap if $\boldsymbol{x} \neq \boldsymbol{x'}$. To see the concentration of $m_k(\boldsymbol{x}, \boldsymbol{x'})$, we consider the variance of $m_k(\boldsymbol{x}, \boldsymbol{x'})$.

$$
\begin{aligned}
\mathrm{Var}_{\boldsymbol{x},\boldsymbol{x'}}\left[m_k(\boldsymbol{x}, \boldsymbol{x'})\right] &= \mathrm{Var}_{\boldsymbol{x},\boldsymbol{x'}}\left[m_k(\boldsymbol{x}, \boldsymbol{x'}) - \frac{1}{2}\right] && (99)\\
&\leqslant \mathbb{E}_{\boldsymbol{x},\boldsymbol{x'}}\left[\left(\mathrm{Tr}[\rho_k(\boldsymbol{x})\rho_k(\boldsymbol{x'})] - \frac{1}{2}\right)^2\right] && (100)\\
&= \mathbb{E}_{\boldsymbol{x},\boldsymbol{x'}}\left[\left(\mathrm{Tr}\left[\left(\rho_k(\boldsymbol{x}) - \frac{\mathbb{1}_k}{2}\right)\rho_k(\boldsymbol{x'})\right]\right)^2\right] && (101)\\
&\leqslant \mathbb{E}_{\boldsymbol{x},\boldsymbol{x'}}\left\|\rho(\boldsymbol{x}) - \frac{\mathbb{1}_k}{2}\right\|_2^2 \|\rho(\boldsymbol{x})\|_2^2 && (102)\\
&\leqslant \mathbb{E}_{\boldsymbol{x}}\left\|\rho(\boldsymbol{x}) - \frac{\mathbb{1}_k}{2}\right\|_2 && (103)\\
&\in \mathcal{O}\left(\frac{1}{b^n}\right), && (104)
\end{aligned}
$$

where the second inequality is by Hölder's inequality and the third inequality is due to $\|\rho_k(\boldsymbol{x})\|_2^2 \leqslant 1$. In addition, we can show the mean itself concentrates towards 1/2.

$$
\begin{aligned}
\left|\mathbb{E}_{\boldsymbol{x},\boldsymbol{x'}}\left[m_k(\boldsymbol{x}, \boldsymbol{x'})\right] - \frac{1}{2}\right| &\leqslant \mathbb{E}_{\boldsymbol{x},\boldsymbol{x'}}\left|m_k(\boldsymbol{x}, \boldsymbol{x'}) - \frac{1}{2}\right| && (105)\\
&= \mathbb{E}_{\boldsymbol{x},\boldsymbol{x'}}\left|\mathrm{Tr}[\rho_k(\boldsymbol{x})\rho_k(\boldsymbol{x'})] - \frac{1}{2}\right| && (106)\\
&= \mathbb{E}_{\boldsymbol{x},\boldsymbol{x'}}\left|\mathrm{Tr}\left[\left(\rho_k(\boldsymbol{x}) - \frac{\mathbb{1}_k}{2} + \frac{\mathbb{1}_k}{2}\right)\rho_k(\boldsymbol{x'})\right] - \frac{1}{2}\right| && (107)\\
&= \mathbb{E}_{\boldsymbol{x},\boldsymbol{x'}}\left|\mathrm{Tr}\left[\left(\rho_k(\boldsymbol{x}) - \frac{\mathbb{1}_k}{2}\right)\rho_k(\boldsymbol{x'})\right]\right| && (108)\\
&\leqslant \mathbb{E}_{\boldsymbol{x},\boldsymbol{x'}}\left\|\rho_k(\boldsymbol{x}) - \frac{\mathbb{1}_k}{2}\right\|_2 \|\rho_k(\boldsymbol{x'})\|_2 && (109)\\
&\leqslant \mathbb{E}_{\boldsymbol{x}}\left\|\rho_k(\boldsymbol{x}) - \frac{\mathbb{1}_k}{2}\right\|_2 && (110)\\
&\in \mathcal{O}\left(\frac{1}{b^n}\right), && (111)
\end{aligned}
$$

where the first inequality is due to Jensen's inequality, in the second inequality we apply Hölder's inequality and the third inequality is due to the fact that $\|\rho_k(\boldsymbol{x})\|_2 \leqslant 1$ for any quantum state. Together, we have found that $m_k(\boldsymbol{x}, \boldsymbol{x'})$ exponentially concentrates towards 1/2. Note that for the purity case, one can repeat the above steps with $\boldsymbol{x} = \boldsymbol{x'}$, which gives the same result.

We now show that, for any given input pair, $m_k(\boldsymbol{x}, \boldsymbol{x'})$ takes a value exponentially close to 1/2 with probability exponentially close to 1 using Chebyshev's inequality together with Eq. (104). Following the same steps as for the local SWAP strategy we obtain

$$
\mathrm{Pr}_{\boldsymbol{x},\boldsymbol{x'}}\left[|m_k(\boldsymbol{x}, \boldsymbol{x'}) - \mu| \leqslant \sqrt{\sigma}\right] \geqslant 1 - \sigma, \tag{112}
$$

with $\mu = \mathbb{E}_{\boldsymbol{x},\boldsymbol{x'}}\left[m_k(\boldsymbol{x}, \boldsymbol{x'})\right]$ and $\sigma^2 = \mathrm{Var}_{\boldsymbol{x},\boldsymbol{x'}}\left[m_k(\boldsymbol{x}, \boldsymbol{x'})\right]$. This implies that, with probability at least $1 - \sigma$ such that $\sigma \in \mathcal{O}(b^{-n/2})$, the quantity $m_k(\boldsymbol{x}, \boldsymbol{x'})$ takes the value within the range between $\mu - \sqrt{\sigma}$ and $\mu + \sqrt{\sigma}$. Furthermore, by using Eq. (111), we conclude that $1/2 - \sqrt{\sigma} - \beta \leqslant m_k(\boldsymbol{x}, \boldsymbol{x'}) \leqslant 1/2 + \sqrt{\sigma} + \beta$ with probability at least $1 - \sigma$.

All that remains is to identify the appropriate fixed distribution. This can be found by replacing $m_k(\boldsymbol{x}, \boldsymbol{x'})$ in $\mathcal{P}_{m_k(\boldsymbol{x},\boldsymbol{x'})}$ with 1/2 (which is the concentration point of the mean) leading to $\widehat{\mathcal{P}}_0 = \{3/4, 1/4\}$. This completes the proof.

□

Supplemental Proposition 3. *Consider the same assumptions as in Supplemental Lemma 5 and suppose a polynomial number of measurement shots is used to estimate the 2-norms. For any input data pair $\boldsymbol{x}$ and $\boldsymbol{x'}$, the following statements hold, with probability exponentially close to 1 (i.e., with probability $1 - \delta_a$ with $\delta_a \in \mathcal{O}(\tilde{c}^{-n})$ for $\tilde{c} > 1$)*

- *For the tomography strategy, the statistical estimate of the 2-norm between two reduced quantum states is statistically indistinguishable (as per Defintion 3) from an input-data-independent random variable*

$$\ell_N^{(\text{rand,T})} = \frac{1}{2}\left[\left(\widehat{\kappa}_{N,1}^{(\text{rand})} - \widehat{\kappa}_{N,2}^{(\text{rand})}\right)^2 + \left(\widehat{\kappa}_{N,3}^{(\text{rand})} - \widehat{\kappa}_{N,4}^{(\text{rand})}\right)^2 + \left(\widehat{\kappa}_{N,5}^{(\text{rand})} - \widehat{\kappa}_{N,6}^{(\text{rand})}\right)^2\right] , \tag{113}$$

  where $\left\{\widehat{\kappa}_{N,i}^{(\text{rand})}\right\}_{i=1}^{6}$ are different instances of $\widehat{\kappa}_N^{(\text{rand})}$ defined in Eq. (54).

- *For the SWAP strategy, the statistical estimate of the 2-norm between two reduced quantum states is statistically indistinguishable from an input-data-independent random variable*

$$\ell_N^{(\text{rand,S})} = \widehat{\kappa}_{N,1}^{(\text{biased rand})} + \widehat{\kappa}_{N,2}^{(\text{biased rand})} - 2\widehat{\kappa}_{N,3}^{(\text{biased rand})} , \tag{114}$$

  where $\left\{\widehat{\kappa}_{N,i}^{(\text{biased rand})}\right\}_{i=1}^{3}$ are different instances of $\widehat{\kappa}_N^{(\text{biased rand})}$ as defined in Eq. (70).

In addition, the estimate of the projected quantum kernel is statistically indistinguishable from an input-data-independent random variable

$$\widehat{\kappa}_N^{(\text{rand,PQ})} = \exp\left[\gamma \sum_{k=1}^{n} \ell_{N,k}^{(\text{rand})}\right] , \tag{115}$$

where $\left\{\ell_{N,k}^{(\text{rand})}\right\}_{k=1}^{n}$ are different instances of either $\ell_N^{(\text{rand,T})}$ for the tomography strategy, or $\ell_N^{(\text{rand,S})}$ for the SWAP strategy.

Proof. To prove this result, we incorporate Supplemental Lemma 5 with a union bound over different terms we need to measure. Since the total number of terms required to be measured on a quantum computer are at most polynomial in the number of qubits, we have that the total probability that all of them are simultaneously indistinguishable remains exponentially close to 1. We note that this proof follows the same steps as Supplemental Corollary 2 (though, we still clearly unfold it for completeness).

First, consider estimating the 2-norm distance between two reduced data-encoded states on the qubit k i.e. $\|\rho_k(\boldsymbol{x}) - \rho_k(\boldsymbol{x'})\|_2$. In either strategy, we construct an estimation of this by measuring a number of different quantities as detailed at the start of Section III B. For the tomography strategy, the 2-norm can be expressed as

$$\|\rho_k(\boldsymbol{x}) - \rho_k(\boldsymbol{x'})\|_2^2 = \frac{1}{2}\left((c_{x_k}(\boldsymbol{x}) - c_{x_k}(\boldsymbol{x'}))^2 + (c_{y_k}(\boldsymbol{x}) - c_{y_k}(\boldsymbol{x'}))^2 + (c_{z_k}(\boldsymbol{x}) - c_{z_k}(\boldsymbol{x'}))^2\right) , \tag{116}$$

which means we are required to measure 6 different expectation values (3 expectation values to reconstruct each reduced state). On the other hand, for the local SWAP strategy, the 2-norm can be expressed as

$$\|\rho_k(\boldsymbol{x}) - \rho_k(\boldsymbol{x'})\|_2^2 = \mathrm{Tr}\left[\rho_k^2(\boldsymbol{x})\right] + \mathrm{Tr}\left[\rho_k^2(\boldsymbol{x'})\right] - 2\,\mathrm{Tr}[\rho_k(\boldsymbol{x})\rho_k(\boldsymbol{x'})] , \tag{117}$$

which implies there are total of 3 different terms to be measured (2 purities and 1 state overlap). When measuring each term (with polynomial measurement shots $N \in \mathcal{O}(\text{poly}(n))$, it follows from Supplemental Lemma 5 that

$$\Pr[E_i^{(k)}] \geqslant 1 - \delta \ , \quad i \in \{1, 2, ..., m\} \ , \tag{118}$$

where $\delta \in \mathcal{O}(b^{-n})$ for some $b > 1$ and m is the total number of terms to be measured i.e., $m = 6$ for the tomography strategy and $m = 3$ for the local SWAP strategy. Here, we have denoted $E_i^{(k)}$ as the event that an estimate of a chosen term (among the m terms) on the qubit k is statistically indistinguishable from a data-independent random variable i.e., $\widehat{\kappa}^{(\text{rand})}$ in Eq. (54) for the tomography strategy, and $\widehat{\kappa}_N^{(\text{biased rand})}$ in Eq. (70)) for the local SWAP strategy. Thus, the probability that all $E_i^{(k)}$ simultaneously occur can be upper bounded as

$$\Pr\left[\bigcap_{i=1}^{m} E_i^{(k)}\right] = 1 - \Pr\left[\bigcup_{i=1}^{m} \bar{E}_i^{(k)}\right] \tag{119}$$

$$\geqslant 1 - \sum_{i=1}^{m} \Pr\left[\bar{E}_i^{(k)}\right] \tag{120}$$

$$\geqslant 1 - m\delta \ , \tag{121}$$

where we denote $\bar{E}_i^{(k)}$ has the conjugate event of $E_i^{(k)}$, the union bound is applied in the second line, and to reach the final line we use the fact that $\Pr[\bar{E}_i^{(k)}] \leqslant \delta_k$ by reversing the inequality in Eq. (118). This shows that for the two considered strategies the estimated 2-norm is indistinguishable from some data-independent random variable with probability exponentially close to 1, in that part of the collected measurement statistics are distinguishable. Specifically, the statistical estimate of the 2-norm from the tomography strategy is indistinguishable from

$$\ell_N^{(\text{rand,T})} = \frac{1}{2}\left[\left(\widehat{\kappa}_{N,1}^{(\text{rand})} - \widehat{\kappa}_{N,2}^{(\text{rand})}\right)^2 + \left(\widehat{\kappa}_{N,3}^{(\text{rand})} - \widehat{\kappa}_{N,4}^{(\text{rand})}\right)^2 + \left(\widehat{\kappa}_{N,5}^{(\text{rand})} - \widehat{\kappa}_{N,6}^{(\text{rand})}\right)^2\right] \ . \tag{122}$$

This is obtained by replacing coefficients in Eq. (116) with $\left\{\widehat{\kappa}_{N,i}^{(\text{rand})}\right\}_{i=1}^{6}$ which are different instances of $\widehat{\kappa}_N^{(\text{rand})}$ defined in Eq. (54). Similarly, the statistical estimate of the 2-norm from the local SWAP strategy is indistinguishable from

$$\ell_N^{(\text{rand,S})} = \widehat{\kappa}_{N,1}^{(\text{biased rand})} + \widehat{\kappa}_{N,2}^{(\text{biased rand})} - 2\widehat{\kappa}_{N,3}^{(\text{biased rand})} \ . \tag{123}$$

where $\left\{\widehat{\kappa}_{N,i}^{(\text{biased rand})}\right\}_{i=1}^{3}$ are different instances of $\widehat{\kappa}_N^{(\text{biased rand})}$ in Eq. (70). This completes the first part of the proof.

For the second half of the proof, we show that the statistical estimate of the projected quantum kernel with polynomial measurement shots is also indistinguishable from some data-independent random variable (with high probability). The projected kernel in Eq. (62) is estimated by (classically) post-processing estimated 2-norms over all single qubit subsystems. It follows from the conclusion of the first half of the proof that these estimated 2-norms on any single qubit subsystem are statistically indistinguishable with probability at least $1 - m\delta$. We note that the estimate of the projected kernel is statistically indistinguishable when all estimated 2-norms on a single qubit subsystem are indistinguishable.

To show this, we again use the union bound. Let $F_k = \bigcap_{i=1}^{m} E_i^{(k)}$ as the event that the estimated 2-norm on the qubit k is indistinguishable. We have that the probability of all F_k simultaneously occur to be bounded as

$$\Pr\left[\bigcap_{k=1}^{n} F_k\right] = 1 - \Pr\left[\bigcup_{k=1}^{n} \bar{F}_k\right] \tag{124}$$

$$\geqslant 1 - \sum_{k=1}^{n} \Pr\left[\bar{F}_k\right] \tag{125}$$

$$\geqslant 1 - nm\delta \ , \tag{126}$$

where the steps follow identically as Eq. (119) to Eq. (121). We note that the total measurement shots spent here are nmN which remains polynomial in the number of qubits.

In other words, for any given input pair, the statistical estimate of the projected kernel is indistinguishable with the polynomial shots from the data-independent random variable $\widehat{\kappa}_N^{(\text{rand,PQ})}$ with the probability at least $1 - nm\delta$ such that

$$\widehat{\kappa}_N^{(\text{rand,PQ})} = \exp\left[\gamma \sum_{k=1}^{n} \ell_{N,k}^{(\text{rand})}\right] , \tag{127}$$

where $\left\{\ell_{N,k}^{(\text{rand})}\right\}_{k=1}^{n}$ are different instances of either $\ell_N^{(\text{rand,T})}$ for the tomography strategy, or $\ell_N^{(\text{rand,S})}$ for the SWAP strategy. □

Supplemental Corollary 3. *Consider a kernel ridge regression task with the projected quantum kernel, a squared loss function and a training dataset* $\mathcal{S} = \{\boldsymbol{x}, y_i\}_{i=1}^{N_s}$ *with* $N_s \in \mathcal{O}(\text{poly}(n))$*. Given that the kernel value is estimated using either the SWAP test or tomography strategies and under the same assumption as in Supplemental Lemma 5, the following statements hold with probability exponentially close to 1 (i.e., with the probability* $1 - \delta_a$ *with* $\delta_a \in \mathcal{O}(\tilde{c}^{-n})$ *for* $\tilde{c} > 1$*)*

- *The estimated Gram matrix* $\widehat{K}$ *is statistically indistinguishable (as per Definition 3) from an input-data-independent matrix* $\widehat{K}_N^{(\text{rand,PQ})}$ *whose diagonal elements are 1 and off-diagonal elements are instances of* $\widehat{\kappa}_N^{(\text{rand,PQ})}$ *determined by Supplemental Proposition 3.*
- *The estimated optimal parameters are statistically indistinguishable from the input-data-independent random variables*

$$\boldsymbol{a}_{\text{rand}}(\boldsymbol{y}, \lambda) = \left(\widehat{K}_N^{(\text{rand,PQ})} - \lambda \mathbb{1}\right)^{-1} \boldsymbol{y} . \tag{128}$$

  where $\boldsymbol{y}$ *is a vector of output data points with its* i^{th} *element equal to* y_i *and* λ *is the regularization parameter.*
- *The model prediction on an unseen input data* $\boldsymbol{x}$ *is statistically indistinguishable from the input data independent random variable*

$$h_{\text{rand}} = \boldsymbol{a}_{\text{rand}}(\boldsymbol{y}, \lambda)^T \boldsymbol{k}_N^{(\text{rand,PQ})} , \tag{129}$$

  where $\boldsymbol{k}_N^{(\text{rand,PQ})}$ *is a random vector where each of its element is an instance of* $\widehat{\kappa}_N^{(\text{rand,PQ})}$ *in Eq. (73).*

Proof. We remark that the proof steps are identical to the proofs of Supplemental Corollary 2 (with the fidelity kernel replaced by the projected kernel). Nevertheless, we fully provide the proof of this result for the completeness and convenience.

To prove our main result here, we combine Supplemental Proposition 3 with a union bound over the individual kernel values. More explicitly, Supplemental Proposition 3 concludes that for any given input pair the estimate of the projected kernel is indistinguishable (with the polynomial shots) from the data-independent random variable $\widehat{\kappa}_N^{(\text{rand,PQ})}$ in Eq. (73) with probability exponentially close to 1. Taking into account a polynomial number of training data $N_s \in \mathcal{O}(\text{poly}(n))$, we are required to measure a polynomial number of kernel values and each kernel follows Supplemental Proposition 3.

Now, consider the Gram matrix which can be constructed by measuring each kernel in the set $\mathcal{K}_{\text{PQ}}$ in Eq. (74). This results in $N_s(N_s - 1)/2$ unique kernel values. To proceed, we denote κ_i as an ith element in $\mathcal{K}_{\text{PQ}}$ with i running from 1 to $|\mathcal{K}_{\text{PQ}}| = N_s(N_s - 1)/2$. In addition, let E_i be the event that the estimate of κ_i is statistically indistinguishable from $\widehat{\kappa}_N^{(\text{rand,PQ})}$. By invoking Supplemental Proposition 3, we have

$$\Pr[E_i] \geqslant 1 - \delta_\kappa \quad , \forall \kappa_i \in \mathcal{K}_{\text{PQ}} , \tag{130}$$

where $\delta_\kappa \in \mathcal{O}(c^{-n})$ for $c > 1$. Then, by applying the union bound, the probability of all E_i occurring at the same time is lower bounded as

$$\Pr\left[\bigcap_i E_i\right] = 1 - \Pr\left[\bigcup_i \bar{E}_i\right] \tag{131}$$

$$\geqslant 1 - \sum_{i=1}^{|\mathcal{K}_{\rm PQ}|} \Pr\left[\bar{E}_i\right] \tag{132}$$

$$\geqslant 1 - \frac{N_s(N_s-1)\delta_k}{2}\ , \tag{133}$$

where we denote $\bar{E}_i$ is a conjugate event of E_i, we use the union bound in the second line and use $\Pr[\bar{E}_i] \leqslant \delta_k$ by reversing the final inequality in Eq. (130). Thanks to $N_s \in \mathcal{O}(\text{poly}(n))$, the probability that each of the kernel values are statistically indistinguishable (and so the Gram matrix is statistically indistinguishable) is $1-\delta_K$ with $\delta_K := N_s(N_s-1)\delta_k/2 \in \mathcal{O}(\tilde{c}^{-n})$ for some $\tilde{c} > 1$.

The statistical indistinguishability of the optimal parameters directly follows from the above result. This is since the optimal parameters are estimated by simply a post-processing of the Gram matrix.

Finally, the indistinguishability of the model prediction can be proven by further considering N_s additional kernel values for the test input data. Hence, the same procedure via the union bound is repeated which leads to the conclusion that all estimated kernel values (from the Gram matrix and new ones associated with a test input) are indistinguishable from $\widehat{\kappa}_N^{(\text{rand},\text{PQ})}$ with probability exponentially close to 1. □

C. Indistinguishability of concentrated quantum states

So far we have discussed how exponential concentration leads to the statistical indistinguishability of samples obtained from quantum computers. Here we extend this by showing that when quantum states concentrate they exhibit a stronger form of indistinguishability. In particular, we show that those quantum states remain indistinguishable even when given coherent access to many copies. Let us begin by stating the standard textbook result of the Helstrom measurement.

Supplemental Lemma 6. *Suppose that one of either ρ or ρ' is provided to us with equal probability. Then, the probability of making right decision which state is given to us using the optimal POVM measurement is*

$$\Pr[\text{“right decision between } \rho \text{ and } \sigma\text{”}] = \frac{1}{2} + \frac{\|\rho-\sigma\|_1}{4}\ . \tag{134}$$

Importantly, when the two states become exponentially close to each other in the one-norm distance $\|\rho-\sigma\|_1 \in \mathcal{O}(1/b^n)$ with $b > 1$, the probability of guessing correctly is exponentially tight to $1/2$. This strong concentration of quantum states can arise due to noise and entanglement.

One could imagine that having access to multiple copies of quantum states and processing them coherently could help improve their distinguishability. However, we formalize in the following that, when the number of copies is polynomial in the number of qubits, the indistinguishability of the states remains.

Supplemental Proposition 4. *Given that m copies of either ρ or σ are provided to us and we are allowed to coherently process all of them at the same time, the probability of making right decision which state is given to us is upper bounded by*

$$\Pr[\text{“right decision between } \rho \text{ and } \sigma\text{”}] \leqslant \frac{1}{2} + \frac{m\|\rho-\sigma\|_1}{4} \tag{135}$$

Proof. By invoking Lemma 6, the probability of guessing correctly given the m copies of the quantum state is

$$\Pr[\text{“right decision between } \rho \text{ and } \sigma\text{”}] = \frac{1}{2} + \frac{\|\rho^{\otimes m}-\sigma^{\otimes m}\|_1}{4}\ . \tag{136}$$

We now upper bound the one-norm as

$$\|\rho^{\otimes m}-\sigma^{\otimes m}\|_1 = \|\rho^{\otimes m}-\left(\rho^{\otimes m-1}\otimes\sigma\right)+\left(\rho^{\otimes m-1}\otimes\sigma\right)-\left(\rho^{\otimes m-2}\sigma^{\otimes 2}\right)+...+\left(\rho\sigma^{\otimes m-1}\right)-\sigma^{\otimes m}\|_1 \tag{137}$$

$$= \|\rho^{\otimes m-1}\otimes(\rho-\sigma)+\rho^{\otimes m-2}\otimes(\rho-\sigma)\otimes\sigma+...+(\rho-\sigma)\otimes\sigma^{\otimes m-1}\|_1 \tag{138}$$

$$\leqslant \|\rho^{\otimes m-1}\otimes(\rho-\sigma)\|_1+\|\rho^{\otimes m-2}\otimes(\rho-\sigma)\otimes\sigma\|_1+...+\|(\rho-\sigma)\otimes\sigma^{\otimes m-1}\|_1 \tag{139}$$

$$\leqslant \|\rho^{\otimes m-1}\|_1\|\rho-\sigma\|_1+\|\rho^{\otimes m-2}\|_1\|\rho-\sigma\|_1\|\sigma\|_1+...+\|\rho-\sigma\|_1\|\sigma^{\otimes m-1}\|_1 \tag{140}$$

$$\leqslant m\|\rho-\sigma\|_1 \ , \tag{141}$$

where the first inequality is the triangle inequality, the second inequality is from $\|A\otimes B\|_p \leqslant \|A\|_p\|B\|_p$ and in the last inequality we use the fact that one-norm of the quantum state is upper bounded by 1. By substituting this upper bound back in the Eq. (136), the proof is completed. □

Supplemental Corollary 4. *Assume quantum states ρ and σ are exponentially close in the one-norm i.e., $\|\rho-\sigma\|_1 \in \mathcal{O}(1/b^n)$ with $b>1$. The quantum states ρ and σ are indistinguishable even if a polynomial number of copies can be processed coherently.*

Proof. This can be proved by plugging $\|\rho-\sigma\|_1 \in \mathcal{O}(1/b^n)$ and $m \in \mathcal{O}(\text{poly}(n))$ in the upper bound of Eq. (136) in Supplemental Proposition 4. □

Crucially, this implies that in the presence of noise any error mitigation techniques using multiple copies of the states to reduce the effect of noise cannot be used to avoid the data-independence of the solution which results from exponential concentration. More comprehensive treatment of the error mitigation is shown in Supplementary Note VIII.

D. Sufficient condition to resolve kernel values

So far we have argued that it is not possible to resolve kernel values with the polynomial number of measurement shots. In this section we formalize a sufficient condition to resolve the kernel values in the presence of exponential concentration. When the quantity $X(\boldsymbol{\alpha})$ is exponentially concentrated towards a fixed value μ for all $\boldsymbol{\alpha}$ (with high probability or deterministically), we can resolve the statistical estimate of $X(\boldsymbol{\alpha})$ from the estimates of some other $X(\boldsymbol{\alpha}')$ only if the *relative* precision is sufficiently large. More concretely, we can quantify this *relative* precision of the estimated $X(\boldsymbol{\alpha})$ by using a relative error

$$\tilde{\epsilon} = \frac{\epsilon}{\sqrt{\text{Var}_{\boldsymbol{\alpha}}[X(\boldsymbol{\alpha})]}} \tag{142}$$

where ϵ is the statistical uncertainty due to finite measurement shots and $\sqrt{\text{Var}_{\boldsymbol{\alpha}}[X(\boldsymbol{\alpha})]}$ characterizes the concentration of $X(\boldsymbol{\alpha})$ over different $\boldsymbol{\alpha}$. In particular, $\tilde{\epsilon} \lesssim 1$ is needed to resolve $X(\boldsymbol{\alpha})$ from some other $X(\boldsymbol{\alpha}')$. The following proposition shows that exponential scaling in measurement shots is indeed required to be in this regime of sufficient resolution.

Supplemental Proposition 5. *Assume that $X(\boldsymbol{\alpha})$ can be estimated as an expectation value of some observable O. In order to resolve $X(\boldsymbol{\alpha})$ from some other $X(\boldsymbol{\alpha}')$ to additive error ϵ and with probability at least $(1-p)$ via Hoeffding's inequality it is sufficient to provide a number of measurement shots N satisfying*

$$N \geqslant \frac{2\|O\|_\infty^2 \log(2/p)}{\tilde{\epsilon}^2 \text{Var}_{\boldsymbol{\alpha}}[X(\boldsymbol{\alpha})]} \ , \tag{143}$$

where $\tilde{\epsilon} \lesssim 1$ is the relative error as defined in Eq. (142). In addition, if $X(\boldsymbol{\alpha})$ is exponentially concentrated to a fixed point μ according to Definition 1 (either deterministically or probabilistically), the number of shots scales as

$$N \in \Omega\left(\frac{b^{2n}}{\tilde{\epsilon}^2}\right) \ ; \ \ b>1 \ , \tag{144}$$

for arbitrary constant success probability, under the assumption $\|O\|_\infty \in \mathcal{O}(1)$.

Proof. Denote $X(\boldsymbol{\alpha}) = \langle O \rangle$, i.e. $X(\boldsymbol{\alpha})$ is the expectation of some observable O. Estimating $X(\boldsymbol{\alpha})$ in practice is done by measuring the observable N times, with each outcome associated with one of the eigenvalues of O. Then, we can estimate the expectation value as

$$\hat{O}_N = \frac{1}{N}\sum_{i=1}^{N} O_i \ , \tag{145}$$

where $O_i \in [\lambda_{min}, \lambda_{max}]$ is the outcome of the ith measurement and can be treated as a random variable, with $\lambda_{\min}$ and $\lambda_{\max}$ being the smallest and the largest eigenvalues of O. Invoking Hoeffding's inequality, we have

$$\Pr\left[|\hat{O}_N - \langle O \rangle| \geqslant \epsilon\right] \leqslant 2\exp\left(-\frac{2N^2\epsilon^2}{\sum_i (\lambda_{\max} - \lambda_{\min})^2}\right) \tag{146}$$

$$\leqslant 2\exp\left(-\frac{N\epsilon^2}{2\|O\|_\infty^2}\right) \ , \tag{147}$$

where in the second line we have used the fact that $\lambda_{\max} - \lambda_{\min} \leqslant \|O\|_\infty$.

Let $p \geqslant 2\exp\left(-\frac{N\epsilon^2}{2\|O\|_\infty^2}\right)$ be an upper bound on this probability. Upon rearranging, we see that the number of shots scales as

$$N \geqslant \frac{2\|O\|_\infty^2 \log(2/p)}{\epsilon^2} = \frac{2\|O\|_\infty^2 \log(2/p)}{\tilde{\epsilon}^2 \mathrm{Var}_{\boldsymbol{\alpha}}[X(\boldsymbol{\alpha})]} \ . \tag{148}$$

to obtain $|\hat{O}_N - \langle O \rangle| \leqslant \epsilon$ with probability at least $(1-p)$. We recall that in order to resolve $X(\boldsymbol{\alpha})$ from $X(\boldsymbol{\alpha'})$, we need $\tilde{\epsilon} \lesssim 1$ in general.

For deterministic exponential concentration, i.e. $|X(\boldsymbol{\alpha}) - \mu| \leqslant \beta \in \mathcal{O}(1/b^n)$, we have $\mathrm{Var}_{\boldsymbol{\alpha}}[X(\boldsymbol{\alpha})] \leqslant \mathbb{E}_{\boldsymbol{\alpha}}[X^2(\boldsymbol{\alpha})] \leqslant \beta^2$. For probabilistic exponential concentration, i.e. $\Pr_{\boldsymbol{\alpha}}\left[|X(\boldsymbol{\alpha}) - \mu| \geqslant \delta\right] \leqslant \beta^2/\delta^2$, we have $\mathrm{Var}_{\boldsymbol{\alpha}}[X(\boldsymbol{\alpha})] = \beta^2 \in \mathcal{O}(1/b^{2n})$. Thus, in both cases, this leads to the number of measurement shots scaling as

$$N \geqslant \frac{2\|O\|_\infty^2 \log(2/p)}{\tilde{\epsilon}^2 \beta^2} \in \Omega\left(\frac{b^{2n}}{\tilde{\epsilon}^2}\right) \ , \tag{149}$$

with probability at least $(1-p)$ for a fixed p, where we have assumed that $\|O\|_\infty \in \mathcal{O}(1)$. □

Again, in the context of quantum kernel, exponential concentration negatively impacts the performance of kernel-based models in the sense that the Gram matrix K cannot be efficiently estimated in practice. Supplemental Corollary 5 shows that an exponential number of measurement shots is required to distinguish K from a fixed matrix K_0 for the fidelity quantum kernel.

Supplemental Corollary 5. *Assume that the fidelity quantum kernel $\kappa(\boldsymbol{x}, \boldsymbol{x'})$ is exponentially concentrated towards a fixed value μ as in Definition 1. Further, denote K_0 as the fixed matrix whose diagonal elements are all 1 and off-diagonal elements are all μ. Then, the number of measurement shots N required to resolve each off-diagonal matrix element of the Gram matrix K from K_0, up to additive error ϵ and for some arbitrary constant success probability, scales asymptotically as*

$$N \in \Omega\left(\frac{N_s^2 b^{2n}}{\tilde{\epsilon}^2}\right) \ , \tag{150}$$

where $\tilde{\epsilon} \lesssim 1$ is the relative error as defined in Definition 1, and we have assumed that statistical fluctuations associated with individual measurement outcomes stay constant.

Proof. For a given training dataset of size N_s, the number of unique off-diagonal elements in the Gram matrix K is $N_s(N_s-1)/2$. Hence, using Supplemental Proposition 5 for each matrix element, this leads to the total number of measurement shots scaling as claimed. □

We remark that one may be able to reduce the quadratic scaling in N_s in the measurement shot scaling in Supplemental Corollary 5 using classical shadow protocols [12, 13]. However, the exponential scaling in the number of qubits n cannot be removed as this already happens at the level of measuring one element of the Gram matrix. Note that we consider the fidelity quantum kernel as an example but the conclusion can be easily extended to the case of the projected quantum kernel.

Thus, the absence of exponential concentration is a necessary condition to enable the potential of quantum kernels. For example, in the case of quantum support vector machine a non-vanishing separation between the two classes obtained from the feature map is essential. In general such embeddings are hard to construct; however, one strategy is to encode the problem structure directly into the embedding. Ref. [9] shows that for a specific encryption-inspired learning task one can build a feature map, based on Shor's algorithm, leading to the absence of exponential concentration. In Ref. [14], this embedding is shown to be a part of a family of so-called "covariant quantum kernels" where the symmetry properties of the target problem are encoded into the embedding. Exploring the extent to which such approaches generalize to other problems is an important direction for future research.

IV. SUPPLEMENTARY NOTE - PROOF OF THEOREM 1: EXPRESSIVITY-INDUCED CONCENTRATION

Here we provide a detailed proof of Theorem 1 which formally relates the expressivity of data-encoded unitaries and kernel concentration. For convenience, we recall the theorem (initially stated in the main text).

Theorem 1 (Expressivity-induced concentration)**.** *Consider the fidelity quantum kernel as defined in Eq. (3) and the projected quantum kernel as defined in Eq. (4) in the main text. Assume that input data* $\boldsymbol{x}$ *and* $\boldsymbol{x'}$ *are drawn from the same distribution, leading to an ensemble of unitaries* $\mathbb{U}_{\boldsymbol{x}}$ *as defined in Eq. (17). We have*

$$\Pr_{\boldsymbol{x},\boldsymbol{x'}}[|\kappa(\boldsymbol{x},\boldsymbol{x'}) - \mathbb{E}_{\boldsymbol{x},\boldsymbol{x'}}[\kappa(\boldsymbol{x},\boldsymbol{x'})]| \geqslant \delta] \leqslant \frac{G_n(\varepsilon_{\mathbb{U}_{\boldsymbol{x}}})}{\delta^2} \ , \tag{151}$$

where $\varepsilon_{\mathbb{U}_{\boldsymbol{x}}} = \|\mathcal{A}_{\mathbb{U}_{\boldsymbol{x}}}(\rho_0)\|_1$ *is the data-dependent expressivity measure over* $\mathbb{U}_{\boldsymbol{x}}$ *defined in Eq. (19), and* $G_n(\varepsilon_{\mathbb{U}_{\boldsymbol{x}}})$ *is a function of* $\varepsilon_{\mathbb{U}_{\boldsymbol{x}}}$ *defined as below.*

1. *For the fidelity quantum kernel* $\kappa(\boldsymbol{x},\boldsymbol{x'}) = \kappa^{FQ}(\boldsymbol{x},\boldsymbol{x'})$*, we have*

$$G_n(\varepsilon_{\mathbb{U}_{\boldsymbol{x}}}) = \beta_{\text{Haar}} + \varepsilon_{\mathbb{U}_{\boldsymbol{x}}}(\varepsilon_{\mathbb{U}_{\boldsymbol{x}}} + 2\sqrt{\beta_{\text{Haar}}}) \ , \tag{152}$$

   where $\beta_{\text{Haar}} = \frac{1}{2^{n-1}(2^n+1)}$.

2. *For the projected quantum kernel* $\kappa(\boldsymbol{x},\boldsymbol{x'}) = \kappa^{PQ}(\boldsymbol{x},\boldsymbol{x'})$*, we have*

$$G_n(\varepsilon_{\mathbb{U}_{\boldsymbol{x}}}) = 4\gamma n(\tilde{\beta}_{\text{Haar}} + \varepsilon_{\mathbb{U}_{\boldsymbol{x}}}) \ , \tag{153}$$

   where $\tilde{\beta}_{\text{Haar}} = \frac{3}{2^{n+1}+2}$.

Proof. We separate the proof into two parts, corresponding to each type of quantum kernel.

Fidelity quantum kernel: our strategy here is to compute the upper bound of the variance of the fidelity quantum kernel

$\kappa^{FQ}(\boldsymbol{x},\boldsymbol{x'})$ and then use Chebyshev's inequality to show kernel concentration. Now consider the following

$$\begin{aligned}
\mathrm{Var}_{\boldsymbol{x},\boldsymbol{x'}}[\kappa^{FQ}(\boldsymbol{x},\boldsymbol{x'})] &\leqslant \mathbb{E}_{\boldsymbol{x},\boldsymbol{x'}}[(\kappa^{FQ}(\boldsymbol{x},\boldsymbol{x'}))^2] && (154)\\
&= \int dU(\boldsymbol{x}) \int dU(\boldsymbol{x'}) \,\mathrm{Tr}\big[U(\boldsymbol{x})\rho_0 U^\dagger(\boldsymbol{x}) U(\boldsymbol{x'})\rho_0 U^\dagger(\boldsymbol{x'})\big]\,\mathrm{Tr}\big[U(\boldsymbol{x})\rho_0 U^\dagger(\boldsymbol{x}) U(\boldsymbol{x'})\rho_0 U^\dagger(\boldsymbol{x'})\big] && (155)\\
&= \int dU(\boldsymbol{x}) \int dU(\boldsymbol{x'}) \,\mathrm{Tr}\big[(U(\boldsymbol{x}))^{\otimes 2}\rho_0^{\otimes 2}(U^\dagger(\boldsymbol{x}))^{\otimes 2}(U(\boldsymbol{x'}))^{\otimes 2}\rho_0^{\otimes 2}(U^\dagger(\boldsymbol{x'}))^{\otimes 2}\big] && (156)\\
&= \mathrm{Tr}\left[\int dU(\boldsymbol{x})(U(\boldsymbol{x}))^{\otimes 2}\rho_0^{\otimes 2}(U^\dagger(\boldsymbol{x}))^{\otimes 2} \int dU(\boldsymbol{x'})(U(\boldsymbol{x'}))^{\otimes 2}\rho_0^{\otimes 2}(U^\dagger(\boldsymbol{x'}))^{\otimes 2}\right] && (157)\\
&= \mathrm{Tr}\big[(\mathcal{V}_{\mathrm{Haar}}(\rho_0) - \mathcal{A}_{\mathbb{U}_{\boldsymbol{x}}}(\rho_0))^2\big]\,, && (158)\\
&= \beta_{\mathrm{Haar}} + \mathrm{Tr}[\mathcal{A}_{\mathbb{U}_{\boldsymbol{x}}}(\rho_0)(\mathcal{A}_{\mathbb{U}_{\boldsymbol{x}}}(\rho_0) - 2\mathcal{V}_{\mathrm{Haar}}(\rho_0))] && (159)
\end{aligned}$$

where the second equality comes from the fact that $\mathrm{Tr}[X]\,\mathrm{Tr}[Y] = \mathrm{Tr}[X\otimes Y]$ and $(AC)\otimes(BD) = (A\otimes B)(C\otimes D)$, in the fourth equality we use the fact that the two integrals are identical due to our starting assumptions and substitute in $\mathcal{A}_{\mathbb{U}_{\boldsymbol{x}}}(\rho_0)$ as defined in Eq. (18) (in the main text), and in the last line we introduce $\beta_{\mathrm{Haar}} = \mathrm{Tr}\big[(\mathcal{V}_{\mathrm{Haar}}(\rho_0))^2\big]$. Additionally, $\beta_{\mathrm{Haar}} = \frac{1}{2^{n-1}(2^n+1)}$ which is the result of explicitly performing Haar integration and assuming that the input states ρ_0 are pure. We then rearrange the expression to get

$$\begin{aligned}
|\mathrm{Var}_{\boldsymbol{x},\boldsymbol{x'}}[\kappa^{FQ}(\boldsymbol{x},\boldsymbol{x'})] - \beta_{\mathrm{Haar}}| &\leqslant |\mathrm{Tr}[\mathcal{A}_{\mathbb{U}_{\boldsymbol{x}}}(\rho_0)(\mathcal{A}_{\mathbb{U}_{\boldsymbol{x}}}(\rho_0) - 2\mathcal{V}_{\mathrm{Haar}}(\rho_0))]| && (160)\\
&\leqslant \mathrm{Tr}[|\mathcal{A}_{\mathbb{U}_{\boldsymbol{x}}}(\rho_0)(\mathcal{A}_{\mathbb{U}_{\boldsymbol{x}}}(\rho_0) - 2\mathcal{V}_{\mathrm{Haar}}(\rho_0))|] && (161)\\
&\leqslant \|\mathcal{A}_{\mathbb{U}_{\boldsymbol{x}}}(\rho_0)\|_2 \|\mathcal{A}_{\mathbb{U}_{\boldsymbol{x}}}(\rho_0) - 2\mathcal{V}_{\mathrm{Haar}}\|_2 && (162)\\
&\leqslant \|\mathcal{A}_{\mathbb{U}_{\boldsymbol{x}}}(\rho_0)\|_2\,(\|\mathcal{A}_{\mathbb{U}_{\boldsymbol{x}}}(\rho_0)\|_2 + 2\|\mathcal{V}_{\mathrm{Haar}}(\rho_0)\|_2) && (163)\\
&\leqslant \varepsilon_{\mathbb{U}_{\boldsymbol{x}}}(\varepsilon_{\mathbb{U}_{\boldsymbol{x}}} + 2\sqrt{\beta_{\mathrm{Harr}}})\,, && (164)
\end{aligned}$$

where the second inequality is due to the triangle inequality (here $|A| = \sqrt{A^\dagger A}$), the third equality follows from the matrix Hölder's inequality, the fourth inequality is again due to another use of the triangle inequality. Finally, in the last inequality we use the monotonicity of the Schatten p-norms, along with the definitions of $\varepsilon_{\mathbb{U}_{\boldsymbol{x}}} = \|\mathcal{A}_{\mathbb{U}_{\boldsymbol{x}}}(\rho_0)\|_1$ and β_{Harr}. Having upper bounded the variance, we can now invoke Chebyshev's inequality to complete the first part of the proof.

Projected quantum kernel: we first note that as $1 - k^{PQ}(\boldsymbol{x},\boldsymbol{x'})$ is always non-negative and bounded by 1. Then, we have

$$\begin{aligned}
\mathrm{Var}_{\boldsymbol{x},\boldsymbol{x'}}[k^{PQ}(\boldsymbol{x},\boldsymbol{x'})] &= \mathrm{Var}_{\boldsymbol{x},\boldsymbol{x'}}[1 - k^{PQ}(\boldsymbol{x},\boldsymbol{x'})] && (165)\\
&\leqslant \mathbb{E}_{\boldsymbol{x},\boldsymbol{x'}}[(1 - k^{PQ}(\boldsymbol{x},\boldsymbol{x'}))^2] && (166)\\
&\leqslant \mathbb{E}_{\boldsymbol{x},\boldsymbol{x'}}[1 - k^{PQ}(\boldsymbol{x},\boldsymbol{x'})] && (167)\\
&= \mathbb{E}_{\boldsymbol{x},\boldsymbol{x'}}\left[1 - e^{-\gamma\sum_{k=1}^n \|\rho_k(\boldsymbol{x}) - \rho_k(\boldsymbol{x'})\|_2^2}\right] && (168)\\
&\leqslant \mathbb{E}_{\boldsymbol{x},\boldsymbol{x'}}\left[\gamma\sum_{k=1}^n \|\rho_k(\boldsymbol{x}) - \rho_k(\boldsymbol{x'})\|_2^2\right] && (169)\\
&= \gamma\sum_{k=1}^n \mathbb{E}_{\boldsymbol{x},\boldsymbol{x'}}\|\rho_k(\boldsymbol{x}) - \rho_k(\boldsymbol{x'})\|_2^2\,, && (170)
\end{aligned}$$

where the second inequality uses $0 \leqslant (1 - k^{PQ}(\boldsymbol{x},\boldsymbol{x'})) \leqslant 1$, the second equality is from substituting in the definition of the projected quantum kernel in Eq. (4) in the main text, and finally the last inequality is due to the fact that $1 - e^{-t} \leqslant t$.

Let us focus on one of the expectation values in the sum in Eq. (170).

$$\mathbb{E}_{\boldsymbol{x},\boldsymbol{x'}}\|\rho_k(\boldsymbol{x})-\rho_k(\boldsymbol{x'})\|_2^2 \leqslant \mathbb{E}_{\boldsymbol{x},\boldsymbol{x'}}\left(\left\|\rho_k(\boldsymbol{x})-\frac{\mathbb{1}_k}{2}\right\|_2+\left\|\rho_k(\boldsymbol{x'})-\frac{\mathbb{1}_k}{2}\right\|_2\right)^2 \tag{171}$$

$$\leqslant \mathbb{E}_{\boldsymbol{x},\boldsymbol{x'}}\left(2\left\|\rho_k(\boldsymbol{x})-\frac{\mathbb{1}_k}{2}\right\|_2^2+2\left\|\rho_k(\boldsymbol{x'})-\frac{\mathbb{1}_k}{2}\right\|_2^2\right) \tag{172}$$

$$=2\mathbb{E}_{\boldsymbol{x}}\left\|\rho_k(\boldsymbol{x})-\frac{\mathbb{1}_k}{2}\right\|_2^2+2\mathbb{E}_{\boldsymbol{x'}}\left\|\rho_k(\boldsymbol{x'})-\frac{\mathbb{1}_k}{2}\right\|_2^2 , \tag{173}$$

where $\mathbb{1}_k$ is the identity matrix on the qubit k. The first inequality is due to the triangle inequality and the second inequality comes from the fact that $(t+s)^2 \leqslant 2t^2+2s^2$. Now, consider

$$\mathbb{E}_{\boldsymbol{x}}\left\|\rho_k(\boldsymbol{x})-\frac{\mathbb{1}_k}{2}\right\|_2^2 = \mathbb{E}_{\boldsymbol{x}}\operatorname{Tr}_k\left[\operatorname{Tr}_{\bar{k}}\left(\rho(\boldsymbol{x})-\mathbb{1}/2^n\right)\operatorname{Tr}_{\bar{k}}\left(\rho(\boldsymbol{x})-\mathbb{1}/2^n\right)\right] \tag{174}$$

$$=\mathbb{E}_{\boldsymbol{x}}\operatorname{Tr}\big[(\rho(\boldsymbol{x})-\mathbb{1}/2^n)\otimes(\rho(\boldsymbol{x})-\mathbb{1}/2^n)(\mathrm{SWAP}_{\mathrm{k_1,k_2}}\otimes\mathbb{1}_{\bar{k}_1,\bar{k}_2})\big] \tag{175}$$

$$=\mathbb{E}_{\boldsymbol{x}}\operatorname{Tr}\big[(U(\boldsymbol{x})\otimes U(\boldsymbol{x}))(\sigma\otimes\sigma)(U^\dagger(\boldsymbol{x})\otimes U^\dagger(\boldsymbol{x}))(\mathrm{SWAP}_{\mathrm{k_1,k_2}}\otimes\mathbb{1}_{\bar{k}_1,\bar{k}_2})\big] \tag{176}$$

$$=\mathbb{E}_{V\sim\mathrm{Haar}}\operatorname{Tr}\big[(V\otimes V)(\sigma\otimes\sigma)(V^\dagger\otimes V^\dagger)(\mathrm{SWAP}_{\mathrm{k_1,k_2}}\otimes\mathbb{1}_{\bar{k}_1,\bar{k}_2})\big]-\operatorname{Tr}\big[\mathcal{A}_{\mathbb{U}_{\boldsymbol{x}}}(\rho_0)(\mathrm{SWAP}_{\mathrm{k_1,k_2}}\otimes\mathbb{1}_{\bar{k}_1,\bar{k}_2})\big] \tag{177}$$

$$\leqslant\big|\mathbb{E}_{V\sim\mathrm{Haar}}\operatorname{Tr}\big[(V\otimes V)(\sigma\otimes\sigma)(V^\dagger\otimes V^\dagger)(\mathrm{SWAP}_{\mathrm{k_1,k_2}}\otimes\mathbb{1}_{\bar{k}_1,\bar{k}_2})\big]\big|+\big|\operatorname{Tr}\big[\mathcal{A}_{\mathbb{U}_{\boldsymbol{x}}}(\rho_0)(\mathrm{SWAP}_{\mathrm{k_1,k_2}}\otimes\mathbb{1}_{\bar{k}_1,\bar{k}_2})\big]\big| \tag{178}$$

$$\leqslant\big|\mathbb{E}_{V\sim\mathrm{Haar}}\operatorname{Tr}\big[(V\otimes V)(\sigma\otimes\sigma)(V^\dagger\otimes V^\dagger)(\mathrm{SWAP}_{\mathrm{k_1,k_2}}\otimes\mathbb{1}_{\bar{k}_1,\bar{k}_2})\big]\big|+\|\mathcal{A}_{\mathbb{U}_{\boldsymbol{x}}}(\rho_0)\|_1\|\mathrm{SWAP}_{\mathrm{k_1,k_2}}\otimes\mathbb{1}_{\bar{k}_1,\bar{k}_2}\|_\infty \tag{179}$$

$$\leqslant\big|\mathbb{E}_{V\sim\mathrm{Haar}}\operatorname{Tr}\big[(V\otimes V)(\sigma\otimes\sigma)(V^\dagger\otimes V^\dagger)(\mathrm{SWAP}_{\mathrm{k_1,k_2}}\otimes\mathbb{1}_{\bar{k}_1,\bar{k}_2})\big]\big|+\|\mathcal{A}_{\mathbb{U}_{\boldsymbol{x}}}(\rho_0)\|_1 \tag{180}$$

$$=\mathbb{E}_{V\sim\mathrm{Haar}}\|\operatorname{Tr}_{\bar{k}}[V\sigma V^\dagger]\|_2^2+\varepsilon_{\mathbb{U}_{\boldsymbol{x}}} , \tag{181}$$

where the indices k and $\bar{k}$ represent the qubit k and the rest of the system excluding k respectively. Further, we introduce k_1, k_2 and $\bar{k}_1, \bar{k}_2$ as two copies of such subsystems. The second equality comes from using SWAP trick where we denote SWAP_{k_1,k_2} as the SWAP operator between k_1 and k_2, in the third equality we denote $\sigma=\rho_0-\mathbb{1}/2^n$, in the fourth equality we substitute in the expressivity measure $\mathcal{A}_{\mathbb{U}_{\boldsymbol{x}}}(\sigma)$ and we note that $\mathcal{A}_{\mathbb{U}_{\boldsymbol{x}}}(\sigma)=\mathcal{A}_{\mathbb{U}_{\boldsymbol{x}}}(\rho_0)$. In addition, the first inequality is due to $s-t\leqslant|s|+|t|$, the second inequality comes from applying the triangle inequality followed by Hölder's inequality to the second term. Finally, in the last inequality we upper bound the second term using the fact that $\mathrm{SWAP}_{\mathrm{k_1,k_2}}\otimes\mathbb{1}_{\bar{k}_1,\bar{k}_2}$ has eigenvalues ±1, we reverse the SWAP trick on the first term, and we recall that $\varepsilon_{\mathbb{U}_{\boldsymbol{x}}}=\|\mathcal{A}_{\mathbb{U}_{\boldsymbol{x}}}(\rho_0)\|_1$.

Next, we evaluate the Haar integration in the first term of (181).

$$\mathbb{E}_{V\sim\mathrm{Haar}}\|\operatorname{Tr}_{\bar{k}}[V\sigma V^\dagger]\|_2^2=\mathbb{E}_{V\sim\mathrm{Haar}}\left\|\operatorname{Tr}_{\bar{k}}[V\rho_0V^\dagger]-\frac{\mathbb{1}_k}{2}\right\|_2^2 \tag{182}$$

$$=\mathbb{E}_{V\sim\mathrm{Haar}}\operatorname{Tr}_k\left[\left(\operatorname{Tr}_{\bar{k}}[V\rho_0V^\dagger]\right)^2\right]-\frac{1}{2} \tag{183}$$

$$=\mathbb{E}_{V\sim\mathrm{Haar}}\operatorname{Tr}\left[\left(V\rho_0V^\dagger\otimes V\rho_0V^\dagger\right)\left(\mathrm{SWAP}_{k_1,k_2}\otimes\mathbb{1}_{\bar{k}_1,\bar{k}_2}\right)\right]-\frac{1}{2} , \tag{184}$$

where in the first equality we substitute back $\sigma=\rho_0-\mathbb{1}/2^n$, the second equality is from explicitly expanding the 2-norm and the third equality is due to the SWAP trick. Due to linearity, $\mathbb{E}_{V\sim\mathrm{Haar}}$ can be moved inside the trace and we further use the standard Haar integral $\mathbb{E}_{V\sim\mathrm{Haar}}[(V\rho_0V^\dagger)\otimes(V\rho_0V^\dagger)]=\frac{\mathbb{1}\otimes\mathbb{1}+\mathrm{SWAP}}{2^n(2^n+1)}$ and the fact that ρ_0 is pure (see

for example Eq. (2.26) in [15]), leading to

$$\mathbb{E}_{V\sim\text{Haar}}\operatorname{Tr}\left[\left(V\rho_0V^\dagger\otimes V\rho_0V^\dagger\right)\left(\text{SWAP}_{k_1,k_2}\otimes\mathbb{1}_{\bar{k}_1,\bar{k}_2}\right)\right]=\operatorname{Tr}\left[\left(\frac{\mathbb{1}\otimes\mathbb{1}+\text{SWAP}}{2^n(2^n+1)}\right)\text{SWAP}_{\text{k}_1,\text{k}_2}\otimes\mathbb{1}_{\bar{k}_1,\bar{k}_2}\right] \tag{185}$$

$$=\frac{2^{2(n-1)}\operatorname{Tr}[(\mathbb{1}_{k_1}\otimes\mathbb{1}_{k_2})\text{SWAP}_{k_1,k_2}]+2^{n-1}\operatorname{Tr}\left[\text{SWAP}^2_{k_1,k_2}\right]}{2^n(2^n+1)} \tag{186}$$

$$=\frac{2^{n-1}+2}{2^n+1}, \tag{187}$$

where in the last line we have used the fact that $\text{SWAP}^2=\mathbb{1}$. By substituting Eq. (187) back into Eq. (184), we have $\mathbb{E}_{V\sim\text{Haar}}\|\operatorname{Tr}_{\bar{k}}[V\sigma V^\dagger]\|_2^2=\tilde{\beta}_{\text{Haar}}=\frac{3}{2^{n+1}+2}$. Altogether, we can now upper bound the variance in (170) as

$$\text{Var}_{\boldsymbol{x},\boldsymbol{x'}}[k^{PQ}(\boldsymbol{x},\boldsymbol{x'})]\leqslant 4\gamma n(\tilde{\beta}_{\text{Haar}}+\varepsilon_{\mathbb{U}_{\boldsymbol{x}}})\,. \tag{188}$$

Upon using Chebyshev's inequality, we complete the proof.

□

A. Extensions of Theorem 1 to different input distributions

In Theorem 1, we assume that both $\boldsymbol{x}$ and $\boldsymbol{x'}$ are averaged over all possible input data, implying that they are drawn from the same distribution. In this section, we relax this assumption and consider a scenario where $\boldsymbol{x}$ and $\boldsymbol{x'}$ are drawn from different distributions leading to different data-embedded unitary ensembles $\mathbb{U}_{\boldsymbol{x}}$ and $\mathbb{U}_{\boldsymbol{x'}}$. We still observe kernel concentration in the same form as in (151) of Theorem 1 but with modified values of $G_n(\varepsilon_{\mathbb{U}_{\boldsymbol{x}}},\varepsilon_{\mathbb{U}_{\boldsymbol{x'}}})$ where $\varepsilon_{\mathbb{U}_{\boldsymbol{x}}}$ and $\varepsilon_{\mathbb{U}_{\boldsymbol{x'}}}$ are expressivity measures averaging over $\boldsymbol{x}$ and $\boldsymbol{x'}$.

1. For the fidelity quantum kernel, $G_n(\varepsilon_{\mathbb{U}_{\boldsymbol{x}}},\varepsilon_{\mathbb{U}_{\boldsymbol{x'}}})=\beta_{\text{Haar}}+\varepsilon_{\mathbb{U}_{\boldsymbol{x}}}\varepsilon_{\mathbb{U}_{\boldsymbol{x'}}}+\sqrt{\beta_{\text{Haar}}}(\varepsilon_{\mathbb{U}_{\boldsymbol{x}}}+\varepsilon_{\mathbb{U}_{\boldsymbol{x'}}})$.

2. For the projected quantum kernel, $G_n(\varepsilon_{\mathbb{U}_{\boldsymbol{x}}},\varepsilon_{\mathbb{U}_{\boldsymbol{x'}}})=2\gamma n(2\tilde{\beta}_{\text{Haar}}+\varepsilon_{\mathbb{U}_{\boldsymbol{x}}}+\varepsilon_{\mathbb{U}_{\boldsymbol{x'}}})$.

Proof. First, consider the fidelity quantum kernel. We revisit (157) in the proof of Theorem 1.

$$\text{Var}_{\boldsymbol{x},\boldsymbol{x'}}[\kappa^{FQ}(\boldsymbol{x},\boldsymbol{x'})]\leqslant\operatorname{Tr}\left[\int dU(\boldsymbol{x})(U(\boldsymbol{x}))^{\otimes 2}\rho_0^{\otimes 2}(U^\dagger(\boldsymbol{x}))^{\otimes 2}\int dU(\boldsymbol{x'})(U(\boldsymbol{x'}))^{\otimes 2}\rho_0^{\otimes 2}(U^\dagger(\boldsymbol{x'}))^{\otimes 2}\right] \tag{189}$$

$$=\operatorname{Tr}\left[(\mathcal{V}_{\text{Haar}}(\rho_0)-\mathcal{A}_{\mathbb{U}_{\boldsymbol{x}}}(\rho_0))(\mathcal{V}_{\text{Haar}}(\rho_0)-\mathcal{A}_{\mathbb{U}_{\boldsymbol{x'}}}(\rho_0))\right] \tag{190}$$

$$=\beta_{\text{Haar}}-\operatorname{Tr}[\mathcal{V}_{\text{Haar}}(\rho_0)\mathcal{A}_{\mathbb{U}_{\boldsymbol{x}}}(\rho_0)]-\operatorname{Tr}\left[\mathcal{V}_{\text{Haar}}(\rho_0)\mathcal{A}_{\mathbb{U}_{\boldsymbol{x'}}}(\rho_0)\right]+\operatorname{Tr}\left[\mathcal{A}_{\mathbb{U}_{\boldsymbol{x}}}(\rho_0)\mathcal{A}_{\mathbb{U}_{\boldsymbol{x'}}}(\rho_0)\right]\,. \tag{191}$$

Similar to before, we rearrange the terms leading to

$$|\text{Var}_{\boldsymbol{x},\boldsymbol{x'}}[\kappa^{FQ}(\boldsymbol{x},\boldsymbol{x'})]-\beta_{\text{Haar}}]|=\left|\operatorname{Tr}\left[\mathcal{A}_{\mathbb{U}_{\boldsymbol{x}}}(\rho_0)\mathcal{A}_{\mathbb{U}_{\boldsymbol{x'}}}(\rho_0)\right]-\operatorname{Tr}[\mathcal{V}_{\text{Haar}}\mathcal{A}_{\mathbb{U}_{\boldsymbol{x}}}(\rho_0)]-\operatorname{Tr}\left[\mathcal{V}_{\text{Haar}}\mathcal{A}_{\mathbb{U}_{\boldsymbol{x'}}}(\rho_0)\right]\right| \tag{192}$$

$$\leqslant\left|\operatorname{Tr}\left[\mathcal{A}_{\mathbb{U}_{\boldsymbol{x}}}(\rho_0)\mathcal{A}_{\mathbb{U}_{\boldsymbol{x'}}}(\rho_0)\right]\right|+|\operatorname{Tr}[\mathcal{V}_{\text{Haar}}\mathcal{A}_{\mathbb{U}_{\boldsymbol{x}}}(\rho_0)]|+\left|\operatorname{Tr}\left[\mathcal{V}_{\text{Haar}}\mathcal{A}_{\mathbb{U}_{\boldsymbol{x'}}}(\rho_0)\right]\right| \tag{193}$$

$$\leqslant\varepsilon_{\mathbb{U}_{\boldsymbol{x}}}\varepsilon_{\mathbb{U}_{\boldsymbol{x'}}}+\sqrt{\beta_{\text{Haar}}}(\varepsilon_{\mathbb{U}_{\boldsymbol{x}}}+\varepsilon_{\mathbb{U}_{\boldsymbol{x'}}})\,, \tag{194}$$

where the first inequality is from the triangle inequality and the second inequality due to Hölder's inequality and the monotonicity of the Schatten p-norms. Hence, we have a bound for the variance as

$$\text{Var}_{\boldsymbol{x},\boldsymbol{x'}}[\kappa^{FQ}(\boldsymbol{x},\boldsymbol{x'})]\leqslant\beta_{\text{Haar}}+\varepsilon_{\mathbb{U}_{\boldsymbol{x}}}\varepsilon_{\mathbb{U}_{\boldsymbol{x'}}}+\sqrt{\beta_{\text{Haar}}}(\varepsilon_{\mathbb{U}_{\boldsymbol{x}}}+\varepsilon_{\mathbb{U}_{\boldsymbol{x'}}})\,. \tag{195}$$

For the projected quantum kernel, the bound of $2\mathbb{E}_x\|\rho_k(\boldsymbol{x})-\mathbb{1}_k/2\|_2^2$ remains unchanged as in (181). However, when assembling terms together in the last step, we need to treat expressivity measures over $\boldsymbol{x}$ and $\boldsymbol{x'}$ to be different. The modification leads to

$$\text{Var}_{\boldsymbol{x},\boldsymbol{x'}}[k^{PQ}(\boldsymbol{x},\boldsymbol{x'})]\leqslant 2\gamma n(2\tilde{\beta}_{\text{Haar}}+\varepsilon_{\mathbb{U}_{\boldsymbol{x}}}+\varepsilon_{\mathbb{U}_{\boldsymbol{x'}}})\,, \tag{196}$$

which completes the proof.

□

V. SUPPLEMENTARY NOTE - PROOF OF THEOREM 2: ENTANGLEMENT-INDUCED CONCENTRATION

In this section, we provide a proof of Theorem 2, describing the concentration of the kernel in terms of concentration of reduced states. The theorem (presented in the main text) is restated below for convenience.

Theorem 2 (Entanglement-induced concentration)**.** *Consider the projected quantum kernel as defined in Eq. (4) in the main text. For a given pair of data-encoded states associated with* $\boldsymbol{x}$ *and* $\boldsymbol{x'}$*, we have*

$$\left|1-\kappa^{PQ}(\boldsymbol{x},\boldsymbol{x'})\right| \leqslant (2\ln 2)\gamma\Gamma_s(\boldsymbol{x},\boldsymbol{x'}) \,, \tag{197}$$

where

$$\Gamma_s(\boldsymbol{x},\boldsymbol{x'}) = \sum_{k=1}^{n}\left[\sqrt{S\left(\rho_k(\boldsymbol{x})\middle\|\frac{\mathbb{1}_k}{2}\right)}+\sqrt{S\left(\rho_k(\boldsymbol{x'})\middle\|\frac{\mathbb{1}_k}{2}\right)}\right]^2 \,, \tag{198}$$

where we denote $S\left(\cdot\|\cdot\right)$ *as the quantum relative entropy,* ρ_k *as a reduced state on qubit* k*, and* $\mathbb{1}_k$ *as the maximally mixed state on qubit* k*.*

Proof. We consider the reduced state on a sub-system of n_s qubits, which we denote as $\rho_s(\boldsymbol{x}) = \mathrm{Tr}_{\bar{s}}[\rho(\boldsymbol{x})]$ where $\mathrm{Tr}_{\bar{s}}[\cdot]$ is the partial trace over the rest of the system $\bar{s}$. We first remark that the trace distance and relative quantum entropy are related via Pinsker's inequality as

$$\left\|\rho_s(\boldsymbol{x})-\frac{\mathbb{1}_s}{2^{n_s}}\right\|_1^2 \leqslant 2\mathrm{ln}2\cdot S\left(\rho_s(\boldsymbol{x})\middle\|\frac{\mathbb{1}_s}{2^{n_s}}\right) \,, \tag{199}$$

where $S(\cdot\|\cdot)$ is the relative von Neumann entropy between two quantum states.

For a given pair of quantum data states $\rho(\boldsymbol{x}),\rho(\boldsymbol{x'})$, we now look at a quantity $\|\rho_s(\boldsymbol{x})-\rho_s(\boldsymbol{x'})\|_2^2$ which is a crucial ingredient to construct the projected quantum kernels (see Eq. (4) in the main text). Consider the following bound:

$$\begin{aligned}
\|\rho_s(\boldsymbol{x})-\rho_s(\boldsymbol{x'})\|_2 &\leqslant \|\rho_s(\boldsymbol{x})-\rho_s(\boldsymbol{x'})\|_1 && (200)\\
&= \left\|\left(\rho_s(\boldsymbol{x})-\frac{\mathbb{1}_s}{2^{n_s}}\right)-\left(\rho_s(\boldsymbol{x'})-\frac{\mathbb{1}_s}{2^{n_s}}\right)\right\|_1 && (201)\\
&\leqslant \left\|\rho_s(\boldsymbol{x})-\frac{\mathbb{1}_s}{2^{n_s}}\right\|_1+\left\|\rho_s(\boldsymbol{x'})-\frac{\mathbb{1}_s}{2^{n_s}}\right\|_1 && (202)\\
&\leqslant \sqrt{2\mathrm{ln}2}\left(\sqrt{S\left(\rho_s(\boldsymbol{x})\middle\|\frac{\mathbb{1}_s}{2^{n_s}}\right)}+\sqrt{S\left(\rho_s(\boldsymbol{x'})\middle\|\frac{\mathbb{1}_s}{2^{n_s}}\right)}\right) \,, && (203)
\end{aligned}$$

where the first inequality comes from the monotonicity of Schatten p norms, the second inequality is due to the triangle inequality and the last inequality is from the inequality in Eq. (199).

For $n_s = 1$ as in the projected quantum kernel, we can upper bound $\left|1-k^{PQ}(\boldsymbol{x},\boldsymbol{x'})\right|$ as

$$\begin{aligned}
\left|1-k^{PQ}(\boldsymbol{x},\boldsymbol{x'})\right| &= \left|1-e^{-\gamma\sum_{k=1}^{n}\|\rho_k(\boldsymbol{x})-\rho_k(\boldsymbol{x'})\|_2^2}\right| && (204)\\
&\leqslant \left|\gamma\sum_{k=1}^{n}\|\rho_k(\boldsymbol{x})-\rho_k(\boldsymbol{x'})\|_2^2\right| && (205)\\
&\leqslant (2\ln 2)\gamma\sum_{k=1}^{n}\left[\sqrt{S\left(\rho_k(\boldsymbol{x})\middle\|\frac{\mathbb{1}_k}{2}\right)}+\sqrt{S\left(\rho_k(\boldsymbol{x'})\middle\|\frac{\mathbb{1}_k}{2}\right)}\right]^2 && (206)
\end{aligned}$$

where we use $1-e^{-t}\leqslant t$ in the first inequality and the second inequality follows from using the inequality in Eq. (203). □

VI. SUPPLEMENTARY NOTE - PROOF OF PROPOSITION 3: GLOBAL-MEASUREMENT-INDUCED CONCENTRATION

We restate Proposition 3 for convenience here, which describes a model of concentration due to global measurement.

Proposition 3 (Global-measurement-induced concentration)**.** *Consider the fidelity quantum kernel as defined in Eq. (3) where the data embedding is of the form* $U(\boldsymbol{x}) = \bigotimes_{k=1}^{n} U_k(x_k)$ *with* x_k *being an input component encoded in the qubit* k*, and* U_k *being a single-qubit rotation about the* y*-axis on the* k*-th qubit. For an input data point* $\boldsymbol{x}$*, assume that all components of* $\boldsymbol{x}$ *are independent and uniformly sampled in* $[-\pi, \pi]$*. Given a product initial state* $\rho_0 = \bigotimes_{k=1}^{n} |0\rangle\langle 0|$*, we have,*

$$\mathrm{Pr}_{\boldsymbol{x},\boldsymbol{x'}}[|\kappa^{FQ}(\boldsymbol{x},\boldsymbol{x'}) - 1/2^n| \geqslant \delta] \leqslant \left(\frac{3}{8}\right)^n \cdot \frac{1}{\delta^2} . \tag{207}$$

Proof. Similar to the proof of Theorem 1, we upper bound the variance of the kernel over the input data and then use Chebyshev's inequality to obtain the concentration bound. The difference here is that we specify the form of the data-embedding as $U(\boldsymbol{x}) = \bigotimes_{k=1}^{n} U_k(x_k)$ and the initial state as $\rho_0 = \bigotimes_{k=1}^{n} \rho_0^{(k)}$. Now consider the following:

$$\begin{aligned}
\mathrm{Var}_{\boldsymbol{x},\boldsymbol{x'}}[\kappa^{FQ}(\boldsymbol{x},\boldsymbol{x'})] &\leqslant \mathbb{E}_{\boldsymbol{x},\boldsymbol{x'}}[(\kappa^{FQ}(\boldsymbol{x},\boldsymbol{x'}))^2] &(208)\\
&= \int dU(\boldsymbol{x}) \int dU(\boldsymbol{x'}) \,\mathrm{Tr}\big[U(\boldsymbol{x})\rho_0 U^\dagger(\boldsymbol{x}) U(\boldsymbol{x'})\rho_0 U^\dagger(\boldsymbol{x'})\big]\,\mathrm{Tr}\big[U(\boldsymbol{x})\rho_0 U^\dagger(\boldsymbol{x}) U(\boldsymbol{x'})\rho_0 U^\dagger(\boldsymbol{x'})\big] &(209)\\
&= \int dU(\boldsymbol{x}) \int dU(\boldsymbol{x'}) \prod_{k=1}^{n} \mathrm{Tr}\Big[U_k(x_k)\rho_0^{(k)} U_k^\dagger(x_k) U_k(x_k')\rho_0^{(k)} U_k^\dagger(x_k')\Big] \\
&\qquad\qquad \times \prod_{k=1}^{n} \mathrm{Tr}\Big[U_k(x_k)\rho_0^{(k)} U_k^\dagger(x_k) U_k(x_k')\rho_0^{(k)} U_k^\dagger(x_k')\Big] &(210)\\
&= \int dU(\boldsymbol{x}) \int dU(\boldsymbol{x'}) \prod_{k=1}^{n} \mathrm{Tr}\Big[(U_k(x_k))^{\otimes 2}(\rho_0^{(k)})^{\otimes 2}(U_k^\dagger(x_k))^{\otimes 2}(U_k(x_k'))^{\otimes 2}(\rho_0^{(k)})^{\otimes 2}(U_k^\dagger(x_k'))^{\otimes 2}\Big] &(211)\\
&= \prod_{k=1}^{n} \mathrm{Tr}\Big[\int dU_k(x_k)(U_k(x_k))^{\otimes 2}(\rho_0^{(k)})^{\otimes 2}(U_k^\dagger(x_k))^{\otimes 2} \int dU_k(x_k')(U_k(x_k'))^{\otimes 2}(\rho_0^{(k)})^{\otimes 2}(U_k^\dagger(x_k'))^{\otimes 2}\Big] &(212)\\
&= \left(\frac{3}{8}\right)^n . &(213)
\end{aligned}$$

In the third equality we have used the fact that since the components of $\boldsymbol{x}$ are independently sampled, then $\int dU(\boldsymbol{x}) = \prod \int dU_k(x_k)$. Then, in the fourth inequality we have used the following result, which can be verified to hold for $U_k(x_k) = e^{-ix_k Y}$, and for $\rho_0^{(k)} = |0\rangle\langle 0|$ via a direct computation:

$$\mathrm{Tr}\Big[\int dU_k(x_k)(U_k(x_k))^{\otimes 2}(\rho_0^{(k)})^{\otimes 2}(U_k^\dagger(x_k))^{\otimes 2} \int dU_k(x_k')(U_k(x_k'))^{\otimes 2}(\rho_0^{(k)})^{\otimes 2}(U_k^\dagger(x_k'))^{\otimes 2}\Big] = \frac{3}{8} . \tag{214}$$

In addition, we can show that the concentration point becomes exponentially small with the number of qubits.

$$\begin{aligned}
\mathbb{E}_{\boldsymbol{x},\boldsymbol{x'}}[\kappa^{\mathrm{FQ}}(\boldsymbol{x},\boldsymbol{x'})] &= \prod_{k=1}^{n} \int dU_k(x_k) \int dU_k(x_k') U_k(x_k') \,\mathrm{Tr}\Big[U_k(x_k)\rho_0^{(k)} U_k^\dagger(x_k)\rho_0^{(k)} U_k^\dagger(x_k')\Big] &(215)\\
&= \frac{1}{2^n} . &(216)
\end{aligned}$$

where each term in the product is evaluated to be 1/2 with $U_k(x_k) = e^{-ix_k Y}$ and $\rho_0^{(k)} = |0\rangle\langle 0|$. □

A. Extension to arbitrary local unitaries

We can further generalize the previous proposition to the case U_k is a general unitary. Now, the following result holds.

Supplemental Proposition 6 (Generalized global-measurement-induced concentration)**.** *Consider the fidelity quantum kernel as defined in Eq. (3) where the data embedding is of the form $U(\boldsymbol{x}) = \bigotimes_{k=1}^{n} U_k(x_k)$ with x_k is the kth element of $\boldsymbol{x}$ encoded in the qubit k. For an input data point $\boldsymbol{x}$, assume that all components of $\boldsymbol{x}$ are independent. Given that the initial state is a product state $\rho_0 = \bigotimes_{k=1}^{n} \rho_0^{(k)}$, we have*

$$\mathrm{Pr}_{\boldsymbol{x},\boldsymbol{x'}}[|\kappa^{FQ}(\boldsymbol{x},\boldsymbol{x'}) - \mu| \geqslant \delta] \leqslant \frac{\prod_{k=1}^{n} G_1^{(k)}(\varepsilon_{\mathbb{U}_{x_k}})}{\delta^2} \ , \tag{217}$$

where $\mu = \mathbb{E}_{\boldsymbol{x},\boldsymbol{x'}}[\kappa^{FQ}(\boldsymbol{x},\boldsymbol{x'})]$ and

$$G_1^{(k)}(\varepsilon_{\mathbb{U}_{x_k}}) = \frac{1}{3} + \varepsilon_{\mathbb{U}_{x_k}} \left(\varepsilon_{\mathbb{U}_{x_k}} + \sqrt{\frac{4}{3}} \right) . \tag{218}$$

Here, $\varepsilon_{\mathbb{U}_{x_k}} = \left\| \mathcal{A}_{\mathbb{U}_{x_k}} \left(\rho_0^{(k)} \right) \right\|_1$ is a data-dependent local expressivity measure of the local unitary $U_k(x_k)$ over all possible values of x_k encoded in qubit k, where $\mathcal{A}_{\mathbb{U}}(\cdot)$ is defined in Eq. (18) in the main text.

Proof. Similar to the proof of Theorem 1, we upper bound the variance of the kernel over the input data and then use Chebyshev's inequality to obtain the concentration bound. The difference here is that we specify the form of the

data-embedding as $U(\boldsymbol{x}) = \bigotimes_{k=1}^n U_k(x_k)$ and the initial state as $\rho_0 = \bigotimes_{k=1}^n \rho_0^{(k)}$. Now consider the following:

$$\begin{aligned}
\mathrm{Var}_{\boldsymbol{x},\boldsymbol{x'}}[\kappa^{FQ}(\boldsymbol{x},\boldsymbol{x'})] &\leqslant \mathbb{E}_{\boldsymbol{x},\boldsymbol{x'}}[(\kappa^{FQ}(\boldsymbol{x},\boldsymbol{x'}))^2] &(219)\\
&= \int dU(\boldsymbol{x}) \int dU(\boldsymbol{x'}) \operatorname{Tr}\left[U(\boldsymbol{x})\rho_0 U^\dagger(\boldsymbol{x}) U(\boldsymbol{x'})\rho_0 U^\dagger(\boldsymbol{x'})\right] \operatorname{Tr}\left[U(\boldsymbol{x})\rho_0 U^\dagger(\boldsymbol{x}) U(\boldsymbol{x'})\rho_0 U^\dagger(\boldsymbol{x'})\right] &(220)\\
&= \int dU(\boldsymbol{x}) \int dU(\boldsymbol{x'}) \prod_{k=1}^n \operatorname{Tr}\left[U_k(x_k)\rho_0^{(k)} U_k^\dagger(x_k) U_k(x_k')\rho_0^{(k)} U_k^\dagger(x_k')\right] \\
&\qquad \times \prod_{k=1}^n \operatorname{Tr}\left[U_k(x_k)\rho_0^{(k)} U_k^\dagger(x_k) U_k(x_k')\rho_0^{(k)} U_k^\dagger(x_k')\right] &(221)\\
&= \int dU(\boldsymbol{x}) \int dU(\boldsymbol{x'}) \prod_{k=1}^n \operatorname{Tr}\left[(U_k(x_k))^{\otimes 2}(\rho_0^{(k)})^{\otimes 2}(U_k^\dagger(x_k))^{\otimes 2}(U_k(x_k'))^{\otimes 2}(\rho_0^{(k)})^{\otimes 2}(U_k^\dagger(x_k'))^{\otimes 2}\right] &(222)\\
&= \prod_{k=1}^n \operatorname{Tr}\left[\int dU_k(x_k)(U_k(x_k))^{\otimes 2}(\rho_0^{(k)})^{\otimes 2}(U_k^\dagger(x_k))^{\otimes 2} \int dU_k(x_k')(U_k(x_k'))^{\otimes 2}(\rho_0^{(k)})^{\otimes 2}(U_k^\dagger(x_k'))^{\otimes 2}\right] \\
& &(223)\\
&= \prod_{k=1}^n \operatorname{Tr}\left[\left(\mathcal{V}_{\mathrm{Haar}}(\rho_0^{(i)}) - \mathcal{A}_{\mathbb{U}_{x_k}}(\rho_0^{(i)})\right)^2\right] &(224)\\
&= \prod_{k=1}^n \left(\operatorname{Tr}\left[\left(\mathcal{V}_{\mathrm{Haar}}(\rho_0^{(i)})\right)^2\right] + \operatorname{Tr}\left[\mathcal{A}_{\mathbb{U}_{x_k}}(\rho_0^{(i)})\left(\mathcal{A}_{\mathbb{U}_{x_k}}(\rho_0^{(i)}) - 2\mathcal{V}_{\mathrm{Haar}}(\rho_0^{(i)})\right)\right]\right) &(225)\\
&\leqslant \prod_{k=1}^n \left(\frac{1}{3} + \left|\operatorname{Tr}\left[\mathcal{A}_{\mathbb{U}_{x_k}}(\rho_0^{(i)})\left(\mathcal{A}_{\mathbb{U}_{x_k}}(\rho_0^{(i)}) - 2\mathcal{V}_{\mathrm{Haar}}(\rho_0^{(i)})\right)\right]\right|\right) &(226)\\
&\leqslant \prod_{k=1}^n \left(\frac{1}{3} + \left\|\mathcal{A}_{\mathbb{U}_{x_k}}(\rho_0^{(i)})\right\|_2 \left\|\mathcal{A}_{\mathbb{U}_{x_k}}(\rho_0^{(i)}) - 2\mathcal{V}_{\mathrm{Haar}}(\rho_0^{(i)})\right\|_2\right) &(227)\\
&\leqslant \prod_{k=1}^n \left[\frac{1}{3} + \varepsilon_{\mathbb{U}_{x_k}}\left(\varepsilon_{\mathbb{U}_{x_k}} + \sqrt{\frac{4}{3}}\right)\right] &(228)
\end{aligned}$$

where the second equality comes from substituting $U(\boldsymbol{x}) = \bigotimes_{k=1}^n U_k(x_k)$ and $\rho_0 = \bigotimes_{k=1}^n \rho_0^{(k)}$ followed by using the trace property $\operatorname{Tr}[X \otimes Y] = \operatorname{Tr}[X]\operatorname{Tr}[Y]$, the fourth equality is due to the assumption that all components of x and x' are independent, the fifth equality is due to the assumption that $\boldsymbol{x}$ and $\boldsymbol{x'}$ are drawn from the same distribution and the use of the definition of the local superoperator $\mathcal{A}_{\mathbb{U}_{x_k}}\left(\rho_0^{(i)}\right)$. In addition, we note that $\operatorname{Tr}\left[\left(\mathcal{V}_{\mathrm{Haar}}(\rho_0^{(i)})\right)^2\right] = \frac{1}{3}$. The inequalities (227) and (228) follow the same steps as (160) to (164) in the proof of Theorem 1. That is, we apply the triangle inequality followed by Hölder's inequality in (227) and we use the monotonicity of Schatten p-norm in (228). In the last step, we also substitute in $\varepsilon_{\mathbb{U}_{x_k}} = \left\|\mathcal{A}_{\mathbb{U}_{x_k}}(\rho_0^{(i)})\right\|_1$. With this upper bound of the variance, we invoke Chebyshev's inequality, leading to our desired result.

□

In the limit where all single-qubit unitaries are random (i.e. $\varepsilon_{U_k} = 0\ \forall k$), the upper bound in (217) takes the value $1/3^n$ and therefore the kernel exponentially concentrates probabilistically.

VII. SUPPLEMENTARY NOTE - PROOF OF THEOREM 3: NOISE-INDUCED CONCENTRATION

In this section, we prove Theorem 3 which formally establishes how noise leads to the concentration of quantum kernels. We first note that a quantum state of n qubits can be expressed in the Pauli basis as

$$\rho = \frac{1}{2^n}(\mathbb{1} + \sum_i a_i \sigma_i) \tag{229}$$

$$= \frac{1}{2^n}(\mathbb{1} + \boldsymbol{a}\cdot\boldsymbol{\sigma}) \, , \tag{230}$$

where a_i is a coefficient associated with a Pauli operator $\sigma_i \in \{\mathbb{1}, X, Y, Z\}^{\otimes n}/\{\mathbb{1}^{\otimes n}\}$. Correspondingly, $\boldsymbol{a}$ is a vector of such coefficients and $\boldsymbol{\sigma}$ is a vector of such Pauli operators. We now provide three lemmas describing the evolution of quantum states under unitary transformations and noise channels.

Supplemental Lemma 7 (Pauli coefficients under unitary transformations)**.** *Consider the Pauli decomposition of a state ρ that takes the form in Eq. (229). $\|\boldsymbol{a}\cdot\boldsymbol{\sigma}\|_p$ is invariant under the unitary transformation $\rho \to U\rho U^\dagger$.*

Proof. The invariance under the transformation is a direct consequence of the linearity of unitary transformations and the unitary invariance of Schatten norms. □

Supplemental Lemma 8 (Pauli coefficients under noise channels)**.** *Consider the Pauli coefficients of a state ρ that takes the form in Eq. (229) under the action of the local Pauli noise channel $\mathcal{N} = \mathcal{N}_1 \otimes ... \otimes \mathcal{N}_n$ where each $\mathcal{N}_j$ acts on qubit j according to Eq. (29) in the main text. Then, we have*

$$\|\boldsymbol{a'}\cdot\boldsymbol{\sigma}\|_2 \leqslant q\|\boldsymbol{a}\cdot\boldsymbol{\sigma}\|_2 \, , \tag{231}$$

where $\boldsymbol{a'}$ are the new Pauli coefficients after the action of noise.

Proof. We have

$$\|\boldsymbol{a'}\cdot\boldsymbol{\sigma}\|_2 = \|\mathcal{N}(\boldsymbol{a}\cdot\boldsymbol{\sigma})\|_2 \tag{232}$$

$$= \left\|\mathcal{N}\left(\sum_i a_i\sigma_i\right)\right\|_2 \tag{233}$$

$$= \left\|\sum_i a_i q_X^{x(i)} q_Y^{y(i)} q_Z^{z(i)} \sigma_i\right\|_2 \tag{234}$$

$$\leqslant \left\|\sum_i a_i q^{x(i)+y(i)+z(i)} \sigma_i\right\|_2 \tag{235}$$

$$\leqslant q\,\|\boldsymbol{a}\cdot\boldsymbol{\sigma}\|_2 \, , \tag{236}$$

where, in Eq. (234) we use the fact that $\mathcal{N}(\sigma_i) = q_X^{x(i)} q_Y^{y(i)} q_Z^{z(i)}\sigma_i$ with $x(i), y(i), z(i)$ being the number of respective single-qubit X, Y, Z Pauli operators that appear in the Pauli string σ_i, the first inequality comes from replacing the coefficients with the noise parameter as defined in Eq. (30), and in the final inequality we use the fact that there is at least one non-identity single-qubit Pauli term in σ_i i.e. $x(i)+y(i)+z(i) \geqslant 1$ (recall that $\sigma_i \in \{\mathbb{1}, X, Y, Z\}^{\otimes n}/\{\mathbb{1}^{\otimes n}\}$). □

Supplemental Lemma 9 (Supplemental Lemma 6 from Ref. [16], adapted)**.** *Consider a quantum state ρ under the action of the local Pauli noise channel $\mathcal{N} = \mathcal{N}_1 \otimes ... \otimes \mathcal{N}_n$ where each $\mathcal{N}_j$ acts on qubit j according to Eq. (29) in the main text. Then, we have*

$$S_2\left(\mathcal{N}(\rho)\middle\|\frac{\mathbb{1}}{2^n}\right) \leqslant q^b S_2\left(\rho\middle\|\frac{\mathbb{1}}{2^n}\right) \, , \tag{237}$$

where $S_2(\cdot\|\cdot)$ is the sandwiched 2-Rényi relative entropy and $b = 1/(2\ln(2)) \approx 0.72$.

Proof. This result is a direct consequence of Corollary 5.6 of Ref. [17]. Let us first restate the general result for convenience: For some density operator γ and probability $p > 0$ consider the channel $\mathcal{A}_{p,\gamma}(\cdot) = p(\cdot) + (1-p)\gamma$. Suppose that some other channel $\mathcal{B}$ satisfies

$$\left\|\Gamma^{-\frac{1}{2}}_{\mathcal{B}(\gamma)} \circ \mathcal{B} \circ \mathcal{A}^{-1}_{p,\gamma} \circ \Gamma^{\frac{1}{2}}_{\gamma}\right\|_{2\to 2} \leqslant 1 \tag{238}$$

where $\mathcal{A}^{-1}_{p,\gamma}$ denotes the inverse map of $\mathcal{A}_{p,\gamma}$ and Γ^{p}_{γ} denotes the map $\Gamma^{p}_{\gamma}(\cdot) = \gamma^{\frac{p}{2}}(\cdot)\gamma^{\frac{p}{2}}$. Then, for all states ρ,

$$S_2\big(\mathcal{B}^{\otimes n}(\rho)\|\mathcal{B}^{\otimes n}\left(\gamma^{\otimes n}\right)\big) \leqslant \alpha(p,\gamma) S_2\big(\rho\|\gamma^{\otimes n}\big) \tag{239}$$

where $\alpha(p,\gamma) = \exp\left(\left(1 - \|\gamma^{-1}\|^{-1}_{\infty}\right)\frac{\ln(p)}{\ln\left(\|\gamma^{-1}\|_{\infty}\right)}\right)$. Now, in our case, we consider $\mathcal{A}_{p,\gamma}$ and $\mathcal{B}$ to act on a single qubit. Then, if one chooses $\mathcal{A}_{p,\gamma}$ to be the single qubit depolarizing channel $\mathcal{D}_{p_d}$ with depolarizing probability p_d and maximally mixed fixed point $\gamma = \frac{\mathbb{1}}{2}$, then (239) implies that if some unital qubit channel $\mathcal{B}$ (which acts trivially on the identity) satisfies

$$\left\|\mathcal{B} \circ \mathcal{D}^{-1}_{p_d}\right\|_{2\to 2} \leqslant 1\,. \tag{240}$$

From the previous, we have for any n-qubit state ρ

$$S_2\left(\mathcal{B}^{\otimes n}(\rho)\middle\|\frac{\mathbb{1}^{\otimes n}}{2^n}\right) = \alpha\big((1-p_d), \mathbb{1}/2\big) S_2\left(\rho\middle\|\frac{\mathbb{1}^{\otimes n}}{2^n}\right) \tag{241}$$

$$\leqslant (1-p_d)^b S_2\left(\rho\middle\|\frac{\mathbb{1}^{\otimes n}}{2^n}\right), \tag{242}$$

where we denote $b = 1/(2\ln(2)) \approx 0.72$.

Now suppose that $\mathcal{B}$ is the single-qubit Pauli noise channel $\mathcal{N}_i$ as defined in (29) in the main text. We can explicitly write the condition (240) as

$$\sup_{X\neq 0} \frac{\|\mathcal{N}_i \circ \mathcal{D}^{-1}_{p_d}(X)\|_2}{\|X\|_2} \leqslant 1. \tag{243}$$

We note that the superoperator (Pauli transfer matrix) of the concatenated channel $\mathcal{N}_i \circ \mathcal{D}^{-1}_{p_d}$ is diagonal with diagonal entries $(1, \frac{q_x}{1-p_d}, \frac{q_y}{1-p_d}, \frac{q_z}{1-p_d})$. Consider an arbitrary complex matrix X decomposed in the Pauli basis as $X = a\mathbb{1} + \boldsymbol{b}\cdot\boldsymbol{\sigma}$, where a is a complex number and $\boldsymbol{b}$ is a vector of complex coefficients. Then one can verify

$$\|X\|_2 = \sqrt{2}\sqrt{|a|^2 + \textstyle\sum_i |b_i|^2}\,, \tag{244}$$

$$\|\mathcal{N}_i \circ \mathcal{D}^{-1}_{p}(X)\|_2 = \sqrt{2}\sqrt{|a|^2 + \textstyle\sum_i \left(\frac{q_i}{1-p_d}\right)^2 |b_i|^2}\,, \tag{245}$$

where the second expression is obtained by reading off the diagonal entries of the superoperator of $\mathcal{N}_i \circ \mathcal{D}^{-1}_{p_d}$. In order to satisfy condition (243), one can pick

$$1 - p_d = \max_{\sigma\in\{X,Y,Z\}} |q_\sigma|\,. \tag{246}$$

Thus, by denoting $q = \max_{\sigma\in\{X,Y,Z\}} |q_\sigma|$ and inspecting (242) we obtain the result as required. □

Now, we are ready to prove Theorem 3 in the main text, which is restated below for convenience.

Theorem 3 (Noise-induced concentration)**.** *Consider the L-layered data embedding circuit defined in Eq. (27) with input state ρ_0 and the layer-wise Pauli noise model defined in Eq. (28) with characteristic noise parameter $q < 1$. The concentration of quantum kernel values may be bounded as follows*

$$|\tilde{\kappa}(\boldsymbol{x}, \boldsymbol{x'}) - \mu| \leqslant F(q, L) \,. \tag{247}$$

1. *For the fidelity quantum kernel $\tilde{\kappa}(\boldsymbol{x}, \boldsymbol{x'}) = \tilde{\kappa}^{FQ}(\boldsymbol{x}, \boldsymbol{x'})$, we have $\mu = 1/2^n$, and*

$$F(q, L) = q^{2L+1} \left\| \rho_0 - \frac{\mathbb{1}}{2^n} \right\|_2 \,. \tag{248}$$

2. *For the projected quantum kernel $\tilde{\kappa}(\boldsymbol{x}, \boldsymbol{x'}) = \tilde{\kappa}^{PQ}(\boldsymbol{x}, \boldsymbol{x'})$, we have $\mu = 1$, and*

$$F(q, L) = (8 \ln 2) \gamma n q^{b(L+1)} S_2 \left(\rho_0 \middle\| \frac{\mathbb{1}}{2^n} \right) \,, \tag{249}$$

where $S_2(\cdot\|\cdot)$ denotes the sandwiched 2-Rényi relative entropy and $b = 1/(2\ln(2)) \approx 0.72$.

Additionally, the noisy data-encoded quantum state $\tilde{\rho}(\boldsymbol{x})$ concentrates towards the maximally mixed state as

$$\left\| \tilde{\rho}(\boldsymbol{x}) - \frac{\mathbb{1}}{2^n} \right\|_2 \leqslant q^{L+1} \left\| \rho_0 - \frac{\mathbb{1}}{2^n} \right\|_2 \,. \tag{250}$$

Proof. First we prove the concentration of noisy quantum states toward the maximally mixed state, following Ref. [16]. We express $\tilde{\rho}(\boldsymbol{x})$ explicitly in terms of its Pauli decomposition as $\tilde{\rho}(\boldsymbol{x}) = \frac{1}{2^n}(\mathbb{1} + \tilde{\boldsymbol{a}} \cdot \boldsymbol{\sigma})$ where $\tilde{\boldsymbol{a}}$ are the coefficients after the noisy embedding in Eq. (28) in the main text. Hence, we have

$$\left\| \tilde{\rho}(\boldsymbol{x}) - \frac{\mathbb{1}}{2^n} \right\|_2 = \left\| \frac{1}{2^n} \tilde{\boldsymbol{a}} \cdot \boldsymbol{\sigma} \right\|_2 \tag{251}$$

$$\leqslant q^{L+1} \left\| \frac{1}{2^n} \boldsymbol{a} \cdot \boldsymbol{\sigma} \right\|_2 \tag{252}$$

$$= q^{L+1} \left\| \rho_0 - \frac{\mathbb{1}}{2^n} \right\|_2 \,, \tag{253}$$

where the inequality comes from repeatedly applying Lemma 7 and Lemma 8 $L+1$ times. This completes the proof of the quantum state concentration. Now we prove the concentration of quantum kernels. Similar to the proof of Theorem 3, we separate the proof into two sub-sections for the fidelity and projected quantum kernels.

Fidelity quantum kernels: Consider a noisy fidelity quantum kernel which can be expressed as

$$\tilde{\kappa}^{FQ}(\boldsymbol{x}, \boldsymbol{x'}) = \mathrm{Tr}[\tilde{\rho}(\boldsymbol{x}) \tilde{\rho}(\boldsymbol{x'})] \tag{254}$$

$$= \mathrm{Tr}[\mathcal{W}_{\boldsymbol{x}}(\rho_0) \mathcal{W}_{\boldsymbol{x'}}(\rho_0)] \tag{255}$$

$$= \mathrm{Tr}\big[\rho_0 \mathcal{W}_{\boldsymbol{x},\boldsymbol{x'}}(\rho_0)\big] \,, \tag{256}$$

where we have denoted the channel $\mathcal{W}_{\boldsymbol{x},\boldsymbol{x'}} = \mathcal{W}_{\boldsymbol{x}}^\dagger \circ \mathcal{W}_{\boldsymbol{x'}}$ which is composed of

$$\mathcal{W}_{\boldsymbol{x},\boldsymbol{x'}} = \mathcal{N} \circ \mathcal{U}_1^\dagger(\boldsymbol{x}_1) \circ \mathcal{N} \cdots \mathcal{N} \circ \mathcal{U}_L^\dagger(\boldsymbol{x}_L) \circ \mathcal{N} \circ \mathcal{U}_L(\boldsymbol{x'}_L) \circ \mathcal{N} \cdots \mathcal{N} \circ \mathcal{U}_1(\boldsymbol{x'}_1) \,, \tag{257}$$

where we have used the fact that the Pauli noise channel in Eq. (29) in the main text is self adjoint. Now, we show the concentration of the fidelity kernel.

$$\left|\tilde{\kappa}^{FQ}(\boldsymbol{x},\boldsymbol{x'}) - \frac{1}{2^n}\right| = \left|\mathrm{Tr}\left[\rho_0 \mathcal{W}_{\boldsymbol{x},\boldsymbol{x'}}(\rho_0)\right] - \frac{1}{2^n}\mathrm{Tr}\left[\rho_0\right]\right| \tag{258}$$

$$= \left|\mathrm{Tr}\left[\rho_0\left(\mathcal{W}_{\boldsymbol{x},\boldsymbol{x'}}(\rho_0) - \frac{\mathbb{1}}{2^n}\right)\right]\right| \tag{259}$$

$$\leqslant \|\rho_0\|_2 \left\|\mathcal{W}_{\boldsymbol{x},\boldsymbol{x'}}(\rho_0) - \frac{\mathbb{1}}{2^n}\right\|_2 \tag{260}$$

$$\leqslant q^{2L+1}\left\|\rho_0 - \frac{\mathbb{1}}{2^n}\right\|_2 , \tag{261}$$

where in the first line we express the noisy quantum kernel as in Eq. (256) and also use $\mathrm{Tr}[\rho_0] = 1$, the first inequality is due to Hölder's inequality, and lastly the second inequality comes from using the fact that $\|\rho_0\|_2 \leqslant 1$ together with repeatedly applying Lemma 7 and Lemma 8 for the noisy quantum channel $\mathcal{W}_{\boldsymbol{x},\boldsymbol{x'}}$ in Eq. (257). We note that this is similar to the proof of quantum state concentration but the number of instances of noise $\mathcal{N}$ is now $2L+1$ as we want to implement $U^\dagger(\boldsymbol{x})U(\boldsymbol{x'})$ instead of $U(\boldsymbol{x})$.

Projected quantum kernels: Here we have

$$\left|1 - \tilde{\kappa}^{PQ}(\boldsymbol{x},\boldsymbol{x'})\right| = \left|1 - e^{-\gamma\sum_{k=1}^n \|\tilde{\rho}_k(\boldsymbol{x}) - \tilde{\rho}_k(\boldsymbol{x'})\|_2^2}\right| \tag{262}$$

$$\leqslant \gamma\sum_{k=1}^n \|\tilde{\rho}_k(\boldsymbol{x}) - \tilde{\rho}_k(\boldsymbol{x'})\|_2^2 \tag{263}$$

$$\leqslant \gamma\sum_{k=1}^n \left(\left\|\tilde{\rho}_k(\boldsymbol{x}) - \frac{\mathbb{1}_k}{2}\right\|_2 + \left\|\tilde{\rho}_k(\boldsymbol{x'}) - \frac{\mathbb{1}_k}{2}\right\|_2\right)^2 \tag{264}$$

$$\leqslant \gamma\sum_{k=1}^n \left(2\left\|\tilde{\rho}_k(\boldsymbol{x}) - \frac{\mathbb{1}_k}{2}\right\|_2^2 + 2\left\|\tilde{\rho}_k(\boldsymbol{x'}) - \frac{\mathbb{1}_k}{2}\right\|_2^2\right) , \tag{265}$$

where the first inequality is due to the standard inequality $1 - e^{-t} \leqslant t$, the second inequality is due to the triangle inequality, the third inequality is due to the fact that $(s+t)^2 \leqslant 2s^2 + 2t^2$. Note that the concentration of the reduced state $\tilde{\rho}_k(\boldsymbol{x})$ can be bounded as

$$\left\|\tilde{\rho}_k(\boldsymbol{x}) - \frac{\mathbb{1}_k}{2}\right\|_2^2 = \mathrm{Tr}_k\left[\mathrm{Tr}_{\bar{k}}\left(\tilde{\rho}(\boldsymbol{x}) - \mathbb{1}/2^n\right)\mathrm{Tr}_{\bar{k}}\left(\tilde{\rho}(\boldsymbol{x}) - \mathbb{1}/2^n\right)\right] \tag{266}$$

$$= \mathrm{Tr}\left[(\tilde{\rho}(\boldsymbol{x}) - \mathbb{1}/2^n)\otimes(\tilde{\rho}(\boldsymbol{x}) - \mathbb{1}/2^n)(\mathrm{SWAP}_{\mathrm{k_1,k_2}}\otimes\mathbb{1}_{\bar{k}_1,\bar{k}_2})\right] \tag{267}$$

$$\leqslant \|(\tilde{\rho}(\boldsymbol{x}) - \mathbb{1}/2^n)\otimes(\tilde{\rho}(\boldsymbol{x}) - \mathbb{1}/2^n)\|_1 \|\mathrm{SWAP}_{\mathrm{k_1,k_2}}\otimes\mathbb{1}_{\bar{k}_1,\bar{k}_2}\|_\infty \tag{268}$$

$$\leqslant \|\tilde{\rho}(\boldsymbol{x}) - \mathbb{1}/2^n\|_1^2 \tag{269}$$

$$\leqslant 2\ln 2\cdot S\left(\tilde{\rho}(\boldsymbol{x})\middle\|\frac{\mathbb{1}}{2^n}\right) \tag{270}$$

$$\leqslant 2\ln 2\cdot S_2\left(\tilde{\rho}(\boldsymbol{x})\middle\|\frac{\mathbb{1}}{2^n}\right) \tag{271}$$

$$\leqslant (2\ln 2)q^{b(L+1)}S_2\left(\rho_0\middle\|\frac{\mathbb{1}}{2^n}\right) \tag{272}$$

where we use the SWAP trick in the second line with $\mathrm{SWAP}_{\mathrm{k_1,k_2}}$ being the SWAP operator between two reduced subsystems, the first inequality comes from Hölder's inequality, in the second line we use the fact that $\mathrm{SWAP}_{\mathrm{k_1,k_2}}\otimes\mathbb{1}_{\bar{k}_1,\bar{k}_2}$

has eigenvalues in $\{1, -1\}$ and that $\|X \otimes Y\|_1 = \|X\|_1 \|Y\|_1$, the third inequality is due to Pinsker's inequality, the fourth inequality is due to the monotonicity of the sandwiched 2-Rényi relative entropy, and finally the last inequality is from repeatedly applying the data-processing inequality and Lemma 9 for each layer of unitaries and noise. Hence, we have the concentration bound of the projected quantum kernel as

$$\left|1 - \tilde{\kappa}^{PQ}(\boldsymbol{x}, \boldsymbol{x'})\right| \leqslant (8 \ln 2) \gamma n q^{b(L+1)} S_2\left(\rho_0 \middle\| \frac{\mathbb{1}}{2^n}\right) , \tag{273}$$

which completes the proof.

□

VIII. SUPPLEMENTARY NOTE - ERROR MITIGATION

Error mitigation (EM) strategies have been widely implemented to reduce the effect of noise in variational quantum algorithms (VQAs) and QML. Despite tremendous success to significantly suppress errors of expectation values, it has been recently shown that current common EM strategies cannot resolve the issue of noise-induced barren plateaus for QNNs [18–20]. In particular, even after applying EM protocols, the cost landscape could remain exponentially flat, or otherwise exponential resources are required to reach a sufficiently high resolution of the expectation values. As estimating quantum kernels in practice requires us to measure expectation values of some operators, the results derived in Ref. [18] can be directly applied to the kernel framework, which we explain in more detail below. Consequently, EM strategies also fails to remove the exponential decay in the noise-induced kernel concentration.

Given that we are interested in a noise-free expectation value $C = \mathrm{Tr}[\rho O]$ of some operator O and n-qubit quantum state of interest ρ, the main purpose of EM strategies is to approximate C under the effect of noise by implementing some protocol which gives us a noise-mitigated quantity C_m. Usually, an EM strategy includes one or more of the following protocols: running some modification of the initial circuit of interest, modifying the observable, utilizing multiple copies of the state of interest, performing classical post-processing. Most of well-known EM strategies can be grouped under a unified framework, which includes Zero-Noise Extrapolation [21–24], Clifford Data Regression [25], Virtual Distillation [26, 27] and Probabilistic Error Cancellation [22, 23]. Within this unified framework, we prepare expectation values of the form

$$E_{\sigma,A,M,k} = \mathrm{Tr}\left[A\left(\sigma^{\otimes M} \otimes |0\rangle\langle 0|^{\otimes k}\right)\right] , \tag{274}$$

where we allow modifications to the original circuit leading to the state σ instead of ρ, M copies of σ and k ancillary qubits are allowed, and we measure some operator A that is allowed to act on up to the entire composite system. Then, the noise-mitigated value C_m can be expressed as a linear combination of $E_{\sigma,A,M,k}$ over different σ, A, M, k, as

$$C_m = \sum_{\sigma,A,M,k \in \mathcal{T}_{\mathrm{EM}}} a_{\sigma,A,M,k} E_{\sigma,A,M,k} , \tag{275}$$

where $a_{\sigma,A,M,k}$ are chosen coefficients and $\mathcal{T}_{\mathrm{EM}}$ is a set containing all relevant indices for the considered EM strategy. As an example, consider Zero Noise Extrapolation (ZNE). In this strategy, the noise strength in the circuit is augmented leading to noisier expectation values, and then the error mitigated value is estimated via extrapolating back to the noiseless regime. Given two noisy expectation values $\tilde{C}(\epsilon_q), \tilde{C}(a\epsilon_q)$ with two different noise strengths ϵ_q and $a\epsilon_q$ for $a > 1$, we can express C_m using the first level of Richard extrapolation as

$$C_m = \frac{a\tilde{C}(\epsilon_q) - \tilde{C}(a\epsilon_q)}{a - 1} , \tag{276}$$

which can serve as a better approximation of C than $\tilde{C}(\epsilon_q)$. Note that this takes the form of the general expression in Eq. (275). For more details about error mitigation, we refer the reader to Ref. [28, 29]. We now quote one of the main results in Ref. [18] which is relevant to our work.

Supplemental Theorem 1 (Theorem 1 and Corollary 1 in Ref. [18]). *Consider an error mitigation strategy that as a step in its protocol, estimates $E_{\sigma,A,M,k}$ as defined in Eq. (274). Suppose that σ is prepared with a circuit consisting of L_σ layers of gates with local depolarizing noise with depolarizing probability p occurring before and after each layer. Under these conditions, $E_{\sigma,A,M,k}$ exponentially concentrates on a state-independent fixed point with increasing circuit depth as*

$$\left| E_{\sigma,A,M,k} - \mathrm{Tr}\left[A \left(\frac{\mathbb{1}^{\otimes M}}{2^{Mn}} \otimes |0\rangle\langle 0|^{\otimes k} \right) \right] \right| \leqslant \sqrt{\ln 4} \|A\|_\infty M n^{1/2} (1-p)^{L_\sigma + 1} \, . \tag{277}$$

In addition, consider a noise-mitigated expectation value C_m constructed via Eq. (275). Assume $\|A\|_\infty \in \mathcal{O}(\mathrm{poly}(n))$ and denote $a_{\max}, M_{\max}$ as the maximum values $a_{\sigma,A,M,k}$ and M in $\mathcal{T}_{\mathrm{EM}}$. Then C_m concentrates towards some fixed point F_0 as

$$|C_m - F_0| \in \mathcal{O}(2^{-bn} a_{\max} |\mathcal{T}_{\mathrm{EM}}| M_{\max}) \, , \tag{278}$$

for some constant $b > 0$ if the circuit depths satisfies $L_\sigma \in \Omega(n)$ for all $\sigma \in \mathcal{T}_{\mathrm{EM}}$.

In the context of quantum kernels, the fidelity quantum kernel $\kappa^{FQ}(\boldsymbol{x}, \boldsymbol{x'})$ can be estimated by executing a circuit $U^\dagger(\boldsymbol{x'})U(\boldsymbol{x})$ and then measuring the expectation value of the projection $O = |\psi_0\rangle\langle\psi_0|$. Alternatively, we can perform a SWAP test to measure the fidelity kernel via $\kappa^{FQ}(\boldsymbol{x}, \boldsymbol{x'}) = \mathrm{Tr}[(\rho(\boldsymbol{x}) \otimes \rho(\boldsymbol{x'}))\mathrm{SWAP}]$. Here, in the context of error mitigation we can regard $\rho(\boldsymbol{x}) \otimes \rho(\boldsymbol{x'})$ as the state of interest and the SWAP operator as the measurement observable.

On the other hand, for the projected quantum kernel, a kernel value $\kappa^{PQ}(\boldsymbol{x}, \boldsymbol{x'})$ can be obtained by first estimating n individual terms $\|\rho_k(\boldsymbol{x}) - \rho_k(\boldsymbol{x'})\|_2^2$ on the quantum computer. Since we consider the reduced states on a single-qubit subsystem, it is efficient to directly estimate $\rho_k(\boldsymbol{x})$ and $\rho_k(\boldsymbol{x'})$ by measuring the expectation values of Pauli operators X, Y and Z on qubit k. Thus, 6 expectation values are required for each pair of states, leading to $6n$ expectation values in total. Alternatively, $\|\rho_k(\boldsymbol{x}) - \rho_k(\boldsymbol{x'})\|_2^2$ can be expressed as

$$\|\rho_k(\boldsymbol{x}) - \rho_k(\boldsymbol{x'})\|_2^2 = \mathrm{Tr}\left[\rho_k^2(\boldsymbol{x})\right] + \mathrm{Tr}\left[\rho_k^2(\boldsymbol{x'})\right] - 2\,\mathrm{Tr}[\rho_k(\boldsymbol{x})\rho_k(\boldsymbol{x'})] \, . \tag{279}$$

We can then measure the purities and the overlap in (279) using the SWAP test, leading to 3 expectation values for each pair of states and $3n$ expectation values in total. After all individual terms are estimated, we can sum them and exponentiate them classically to obtain the kernel value.

In all cases, we can see that measuring quantum kernels in practice requires estimating the expectation values of some observable. Therefore, Supplemental Theorem 1 can be directly applied. Consequently, EM strategies which prepare the noise-mitigated expectation value according to Eq. (275) cannot mitigate the exponential concentration of kernel values due to the effect of noise. We note that, for small L we do not rule out that error mitigation can indeed offer improvements. However, as found in Ref. [18], even when considering fixed system size, error mitigation can often impair resolvability compared to applying no error mitigation at all.

IX. SUPPLEMENTARY NOTE - PROOF OF PROPOSITION 4: CONCENTRATION OF KERNEL TARGET ALIGNMENT

In this section, we provide a proof of Proposition 4 (in the main text), showing that the concentration of the kernel target alignment in Eq. (36) in the main text can be upper bounded by the concentration of parametrized quantum kernels. We first present some useful lemmas.

Supplemental Lemma 10 (Variance of sum of correlated random variables). *For a collection of N_s correlated random variables $\{R_i\}_{i=1}^{N_s}$, we have*

$$\mathrm{Var}\left[\sum_i R_i\right] \leqslant N_s \sum_i \mathrm{Var}[R_i] \, . \tag{280}$$

Proof. The variance of the sum of two correlated random variables is given by

$$\mathrm{Var}[R_1+R_2] = \mathrm{Var}[R_1]+\mathrm{Var}[R_2]+2\mathrm{Cov}[R_1,R_2]\;, \tag{281}$$
$$\leqslant \mathrm{Var}[R_1]+\mathrm{Var}[R_2]+2\sqrt{\mathrm{Var}[R_1]\mathrm{Var}[R_2]}\;, \tag{282}$$
$$\leqslant \mathrm{Var}[R_1]+\mathrm{Var}[R_2]+\sqrt{\mathrm{Var}[R_1]\mathrm{Var}[R_1]}+\sqrt{\mathrm{Var}[R_2]\mathrm{Var}[R_2]}\;, \tag{283}$$
$$= 2\mathrm{Var}[R_1]+2\mathrm{Var}[R_2]\;, \tag{284}$$

where in the first inequality we have used Cauchy-Schwarz, and the second inequality comes from the rearrangement inequality. Using induction along with the fact that $\mathrm{Cov}(R_1+R_2,R_3)=\mathrm{Cov}(R_1,R_3)+\mathrm{Cov}(R_2,R_3)$, the variance of the full sum can be bounded as presented. □

Supplemental Lemma 11 (Variance of product)**.** *Given two correlated random variables X and Y, we have*

$$\mathrm{Var}[XY]\leqslant 2\mathrm{Var}[X]|Y^2|_{max}+2(\mathbb{E}[X])^2\mathrm{Var}[Y]\,, \tag{285}$$

where $|Y^2|_{max}$ is the maximum possible value of Y^2 i.e. $|Z|_{max}=\max\{|Z|:\mathrm{Pr(Z)}>0\}$.

Proof. We have

$$\mathrm{Var}[X+Y]=\mathrm{Var}[X]+\mathrm{Var}[Y]+2\mathrm{Cov}[X,Y] \tag{286}$$
$$\leqslant \mathrm{Var}[X]+\mathrm{Var}[Y]+2\sqrt{\mathrm{Var}[X]\mathrm{Var}[Y]} \tag{287}$$
$$\leqslant \mathrm{Var}[X]+\mathrm{Var}[Y]+\sqrt{\mathrm{Var}[X]\mathrm{Var}[X]}+\sqrt{\mathrm{Var}[Y]\mathrm{Var}[Y]} \tag{288}$$
$$=2\mathrm{Var}[X]+2\mathrm{Var}[Y]\,, \tag{289}$$

where in the first inequality we have used Cauchy-Schwarz, and the second inequality comes from the rearrangement inequality. Now consider

$$\mathrm{Var}[XY]=\mathrm{Var}\big[(X-\mathbb{E}[X])Y+\mathbb{E}[X]Y\big] \tag{290}$$
$$\leqslant 2\mathrm{Var}\big[(X-\mathbb{E}[X])Y\big]+2\mathrm{Var}\big[\mathbb{E}[X]Y\big] \tag{291}$$
$$\leqslant 2\mathbb{E}\big[(X-\mathbb{E}[X])^2Y^2\big]+2(\mathbb{E}[X])^2\mathrm{Var}[Y] \tag{292}$$
$$\leqslant 2\mathbb{E}\big[(X-\mathbb{E}[X])^2\big]|Y^2|_{max}+2(\mathbb{E}[X])^2\mathrm{Var}[Y] \tag{293}$$
$$=2\mathrm{Var}[X]|Y^2|_{max}+2(\mathbb{E}[X])^2\mathrm{Var}[Y]\,, \tag{294}$$

where in the first inequality we have used Eq. (289), in the second inequality we have used the definition of the variance, and in the third inequality we have simply taken the maximum value for Y^2. □

Supplemental Lemma 12. *Given a positive bounded random variable X, whose minimum value $|X|_{\min}$ is strictly non-zero, and whose maximum value is $|X|_{\max}$. Then, the following inequality holds*

$$\mathrm{Var}\left[1/\sqrt{X}\right]\leqslant\left(\frac{1}{2|X|_{\min}^3}+\frac{9(|X|_{\max}-|X|_{\min})^2}{32|X|_{\min}^5}\right)\mathrm{Var}[X]\;. \tag{295}$$

Proof. Let us denote $f(X)=1/\sqrt{X}$. The truncated Taylor expansion of $f(X)$ around $X_0=\mathbb{E}[X]$ up to order p can be expressed as

$$f_p(X)=\sum_{m=0}^{p}\frac{f^{(m)}(X_0)}{m!}(X-X_0)^m \tag{296}$$
$$=\sum_{m=0}^{p}\left(\frac{(-1)^m(2m)!}{2^{2m}(m!)^2}\right)\cdot\frac{(X-X_0)^m}{X_0^{m+1/2}}\,, \tag{297}$$

where $f^{(m)}(X_0)$ is the m-order derivative evaluated at X_0. The second equality is the result of explicitly computing the derivatives. Truncating the series to the first order ($p = 1$) gives $f_1(X) = \frac{X-X_0}{2X_0^{3/2}}$. The difference $R_p(X)$ between $f(X)$ and $f_p(X)$ can be bounded using Taylor's remainder theorem (see, for example, Chapter 20 of Ref. [30])

$$R_p(X) \leqslant \frac{\max_{Z\in[X_0,X]} |f^{(p+1)}(Z)|}{(p+1)!} |X - X_0|^{p+1} . \tag{298}$$

For $p = 1$, we have

$$R_1(X) \leqslant \frac{3(X - X_0)^2}{8|X|_{\min}^{5/2}} . \tag{299}$$

Then, the variance of $f(X)$ can then be upper bounded as

$$\begin{aligned}
\mathrm{Var}[f(X)] &= \mathrm{Var}[f_1(X) + R_1(X)] &(300)\\
&\leqslant 2\mathrm{Var}[f_1(x)] + 2\mathrm{Var}[R_1(X)] &(301)\\
&= \frac{\mathrm{Var}[X]}{2X_0^3} + 2\mathrm{Var}[R_1(X)] &(302)\\
&\leqslant \frac{\mathrm{Var}[X]}{2X_0^3} + 2|R_1|_{\max}\mathbb{E}[R_1(X)] &(303)\\
&\leqslant \frac{\mathrm{Var}[X]}{2X_0^3} + 2\left(\frac{3|X - X_0|_{\max}^2}{8|X|_{\min}^{5/2}}\right) \cdot \mathbb{E}\left[\frac{3(X - X_0)^2}{8|X|_{\min}^{5/2}}\right] &(304)\\
&\leqslant \frac{\mathrm{Var}[X]}{2|X|_{\min}^3} + \frac{9(|X|_{\max} - |X|_{\min})^2}{32|X|_{\min}^5}\mathbb{E}\left[(X - X_0)^2\right] &(305)\\
&= \left(\frac{1}{2|X|_{\min}^3} + \frac{9(|X|_{\max} - |X|_{\min})^2}{32|X|_{\min}^5}\right)\mathrm{Var}[X] &(306)
\end{aligned}$$

where the first inequality is due to the variance of the sum in Lemma 10, the second equality is due explicitly evaluating $\mathrm{Var}[f_1(X)]$, the second inequality is from $\mathrm{Var}[R_1(X)] \leqslant \mathbb{E}[(R_1(X))^2] \leqslant |R_1(X)|_{\max}\mathbb{E}[R_1(X)]$, in the third inequality we have used Eq. (299), in the fourth inequality we have used $|X|_{\min} \leqslant X_0$ for the denominator of the term in the brackets and taken the minimum value of $|X|_{\min}^{5/2}$ in the expectation together with $|X - X_0|_{\max}^2 \leqslant (|X|_{\max} - |X|_{\min})^2$. In the last line, we recall that $X_0 = \mathbb{E}[X]$ and hence $\mathbb{E}[(X - X_0)^2] = \mathrm{Var}[X]$.

□

We are now ready to prove our proposition (in the main text) relating concentration of the kernel target alignment with the concentration of the kernel, which is recalled below for convenience.

Proposition 4 (Concentration of kernel target alignment)**.** *Consider an arbitrary parameterized kernel $\kappa_{\boldsymbol{\theta}}(\boldsymbol{x}, \boldsymbol{x'})$ and a training dataset $\{\boldsymbol{x}_i, y_i\}_{i=1}^{N_s}$ for binary classification with $y_i = \pm 1$. The probability that the kernel target alignment $\mathrm{TA}(\boldsymbol{\theta})$ (defined in Eq. (36) in the main text) deviates from its mean value is approximately bounded as*

$$\mathrm{Pr}_{\boldsymbol{\theta}}[|\mathrm{TA}(\boldsymbol{\theta}) - \mathbb{E}_{\boldsymbol{\theta}}[\mathrm{TA}(\boldsymbol{\theta})]| \geqslant \delta] \leqslant \frac{M\sum_{i,j} \mathrm{Var}_{\boldsymbol{\theta}}[\kappa_{\boldsymbol{\theta}}(\boldsymbol{x}_i, \boldsymbol{x}_j)]}{\delta^2} , \tag{307}$$

with $M = \frac{8+N_s^3\left(9(N_s-1)^2+16\right)}{4N_s}$.

Proof. We recall the kernel target alignment $\mathrm{TA}(\boldsymbol{\theta})$ in Eq. (36) in the main text

$$\mathrm{TA}(\boldsymbol{\theta}) = \frac{\sum_{i,j} y_i y_j \kappa_{\boldsymbol{\theta}}(x_i, x_j)}{\sqrt{\left(\sum_{i,j}(\kappa_{\boldsymbol{\theta}}(x_i, x_j))^2\right)\left(\sum_{i,j}(y_i y_j)^2\right)}} \tag{308}$$

$$= \frac{\sum_{i,j} y_i y_j \kappa_{\boldsymbol{\theta}}(x_i, x_j)}{\sqrt{D_A(\boldsymbol{\theta}) \sum_{i,j}(y_i y_j)^2}} \,, \tag{309}$$

where we define $D_A(\boldsymbol{\theta}) = \sum_{i,j}(\kappa_{\boldsymbol{\theta}}(x_i, x_j))^2$. We remark that, as the kernel is normalized and the sum is over all the training data, the minimum value of $D_A(\boldsymbol{\theta})$ (over all possible kernel-based models) happens when $\kappa_{\boldsymbol{\theta}}(x_i, x_j) = 0$ for all $i \neq j$, leading to

$$|D_A|_{min} = N_s \,. \tag{310}$$

Similarly, the maximum value of $D_A(\boldsymbol{\theta})$ (over all possible kernel-based models) is upper bounded with the scenario where $\kappa_{\boldsymbol{\theta}}(x_i, x_j) = 1$ for all i and j, leading to

$$|D_A|_{max} = N_s^2 \,. \tag{311}$$

We now consider the variance of the kernel target alignment (36) in the main text and, again, the concentration bound can be shown via Chebyshev's inequality.

$$\mathrm{Var}_{\boldsymbol{\theta}}[\mathrm{TA}(\boldsymbol{\theta})] = \mathrm{Var}_{\boldsymbol{\theta}}\left[\frac{\sum_{i,j} y_i y_j \kappa_{\boldsymbol{\theta}}(x_i, x_j)}{\sqrt{D_A(\boldsymbol{\theta}) \sum_{i',j'}(y_{i'} y_{j'})^2}}\right] \tag{312}$$

$$\leqslant N_s^2 \sum_{i,j} \mathrm{Var}_{\boldsymbol{\theta}}\left[\frac{y_i y_j \kappa_{\boldsymbol{\theta}}(\boldsymbol{x}_i, \boldsymbol{x}_j)}{\sqrt{D_A(\boldsymbol{\theta}) \sum_{i',j'}(y_{i'} y_{j'})^2}}\right] \tag{313}$$

$$= \sum_{i,j} \mathrm{Var}_{\boldsymbol{\theta}}\left[\frac{\kappa_{\boldsymbol{\theta}}(\boldsymbol{x}_i, \boldsymbol{x}_j)}{\sqrt{D_A(\boldsymbol{\theta})}}\right] \tag{314}$$

$$\leqslant \sum_{i,j}\left(2\mathrm{Var}_{\boldsymbol{\theta}}[\kappa_{\boldsymbol{\theta}}(\boldsymbol{x}_i, \boldsymbol{x}_j)] \cdot \left(\frac{1}{|D_A|_{\min}}\right) + 2(\mathbb{E}_{\boldsymbol{\theta}}[\kappa_{\boldsymbol{\theta}}(\boldsymbol{x}_i, \boldsymbol{x}_j)])^2 \mathrm{Var}_{\boldsymbol{\theta}}\left[\frac{1}{\sqrt{D_A(\boldsymbol{\theta})}}\right]\right) \tag{315}$$

$$\leqslant \sum_{i,j}\left(\frac{2\mathrm{Var}_{\boldsymbol{\theta}}[\kappa_{\boldsymbol{\theta}}(\boldsymbol{x}_i, \boldsymbol{x}_j)]}{N_s} + 2\mathrm{Var}_{\boldsymbol{\theta}}\left[\frac{1}{\sqrt{D_A(\boldsymbol{\theta})}}\right]\right) \tag{316}$$

where the first inequality is due to Lemma 10, the second equality is from $\mathrm{Var}[cX] = c^2\mathrm{Var}[X]$ for a constant c and evaluating $\frac{(y_i y_j)^2}{\sum_{i',j'}(y_{i'} y_{j'})^2} = \frac{1}{N_s^2}$ thanks to $(y_i y_j)^2 = 1 \; \forall i, j$, the second inequality comes from using Lemma 11 and the last inequality is due to the fact that $\mathbb{E}_{\boldsymbol{\theta}}[\kappa_{\boldsymbol{\theta}}(\boldsymbol{x}_i, \boldsymbol{x}_j)] \leqslant 1$.

We now focus on the variance of $1/\sqrt{D_A(\boldsymbol{\theta})}$. We have

$$\mathrm{Var}_{\boldsymbol{\theta}}\left[\frac{1}{\sqrt{D_A(\boldsymbol{\theta})}}\right] \leqslant \left(\frac{1}{2|D_A|^3_{\min}} + \frac{9(|D_A|_{\max} - |D_A|_{\min})^2}{32|D_A|^5_{\min}}\right)\mathrm{Var}[D_A(\boldsymbol{\theta})] \tag{317}$$

$$= \left(\frac{16 + 9(N_s-1)^2}{32N_s^3}\right)\mathrm{Var}_{\boldsymbol{\theta}}\left[\sum_{ij}\kappa^2_{\boldsymbol{\theta}}(\boldsymbol{x}_i, \boldsymbol{x}_j)\right] \tag{318}$$

$$\leqslant \left(\frac{16 + 9(N_s-1)^2}{32N_s^3}\right)N_s^2\sum_{i,j}\mathrm{Var}_{\boldsymbol{\theta}}\left[\kappa^2_{\boldsymbol{\theta}}(\boldsymbol{x}_i, \boldsymbol{x}_j)\right] \tag{319}$$

$$\leqslant \left(\frac{16 + 9(N_s-1)^2}{8N_s}\right)\sum_{i,j}\mathrm{Var}_{\boldsymbol{\theta}}[\kappa_{\boldsymbol{\theta}}(\boldsymbol{x}_i, \boldsymbol{x}_j)] \tag{320}$$

where the first inequality is from using Lemma 12, the first equality is from substituting $|D_A|_{\min}$ in Eq. (310) and $|D_A|_{\max}$ in Eq. (311), the second inequality is due to Lemma 10, and finally the third inequality is from using Lemma 11 followed by $\mathbb{E}_{\boldsymbol{\theta}}[\kappa_{\boldsymbol{\theta}}(\boldsymbol{x}_i, \boldsymbol{x}_j)] \leqslant 1$. Substituting Eq. (320) back into Eq. (316) leads to

$$\mathrm{Var}_{\boldsymbol{\theta}}[\mathrm{TA}(\boldsymbol{\theta})] \leqslant \left(\frac{8 + N_s^2\left(9(N_s-1)^2 + 16\right)}{4N_s}\right)\sum_{i,j}\mathrm{Var}_{\boldsymbol{\theta}}[\kappa_{\boldsymbol{\theta}}(\boldsymbol{x}_i, \boldsymbol{x}_j)]\,. \tag{321}$$

Using Chebyshev's inequality leads us to the desired concentration result. □

X. SUPPLEMENTARY NOTE - SOURCES THAT LEAD TO EXPONENTIALLY FLAT LANDSCAPE OF PARAMETERIZED QUANTUM KERNELS

Proposition 4 establishes that the training landscape of the kernel target alignment $\mathrm{TA}(\boldsymbol{\theta})$ can be analyzed at the level of the parameterized quantum kernels $\kappa_{\boldsymbol{\theta}}(\boldsymbol{x}, \boldsymbol{x}')$. Namely, if the training landscape of $\kappa_{\boldsymbol{\theta}}(\boldsymbol{x}, \boldsymbol{x}')$ with respect to the variational parameters $\boldsymbol{\theta}$ is exponentially flat in the number of qubits n, then the training landscape of $\mathrm{TA}(\boldsymbol{\theta})$ also suffers the same fate. In this section, we investigate features of the parameterized data embedding $U(\boldsymbol{x}, \boldsymbol{\theta})$ that lead to an exponentially flat training landscape of the parameterized quantum kernels. In particular, when designing the parameterized quantum kernels, features that induce barren plateaus in QNNs should be avoided. These include the *expressivity of the training block*, *entanglement*, *global measurements* and *noise*. We note that, although the proofs of the following results are similar to those in the previous sections, the implication of the results is different. While the kernel concentration in the previous sections happens due to the input data, here the training flat landscape is due to the variational part of the parameterized data embedding.

A. Expressivity

Similar to the ensemble of data-encoded unitaries over the possible input data, we can define an ensemble of parametrized unitaries $U(\boldsymbol{x}, \boldsymbol{\theta})$ for a given input data $\boldsymbol{x}$ over variational parameters $\boldsymbol{\theta}$ sampled from a domain Θ. That is, for $\boldsymbol{\theta} \in \Theta$, we have the ensemble $\mathbb{U}_{\boldsymbol{\theta}}(\boldsymbol{x})$ for a given $\boldsymbol{x}$

$$\mathbb{U}_{\boldsymbol{\theta}}(\boldsymbol{x}) = \{U(\boldsymbol{x}, \boldsymbol{\theta}) | \boldsymbol{\theta} \in \Theta\}\,. \tag{322}$$

Then, the expressivity can be measured using the superoperator (18) in the main text with $\mathbb{U} = \mathbb{U}_{\boldsymbol{\theta}}(\boldsymbol{x})$.

Supplemental Theorem 2. *Consider the parametrized fidelity quantum kernel and the parametrized projected quantum kernel defined in Eq. (4) in the main text associated with the parametrized data embedding $U(\boldsymbol{x}, \boldsymbol{\theta})$. For a given pair of*

input data $\boldsymbol{x}$ *and* $\boldsymbol{x'}$*, we have*

$$\Pr_{\boldsymbol{\theta}}[|\kappa_{\boldsymbol{\theta}}(\boldsymbol{x},\boldsymbol{x'})-\mu|\geqslant\delta]\leqslant\frac{\tilde{G}_n(\varepsilon_{\mathbb{U}_{\boldsymbol{\theta}}(\boldsymbol{x})},\varepsilon_{\mathbb{U}_{\boldsymbol{\theta}}(\boldsymbol{x'})})}{\delta^2};, \tag{323}$$

where $\mu=\mathbb{E}_{\boldsymbol{\theta}}[\kappa_{\boldsymbol{\theta}}(\boldsymbol{x},\boldsymbol{x'})]$ *and* $\varepsilon_{\mathbb{U}_{\boldsymbol{\theta}}(\boldsymbol{x})}=\|\mathcal{A}_{\mathbb{U}_{\boldsymbol{\theta}}(\boldsymbol{x})}(\rho_0)\|_1$ *is the expressivity measure over* $\mathbb{U}_{\boldsymbol{\theta}}(\boldsymbol{x})$ *as defined in Eq. (322) with* $\mathcal{A}_{\mathbb{U}_{\boldsymbol{\theta}}(\boldsymbol{x})}(\cdot)$ *defined in Eq. (18) in the main text.*

1. *For the fidelity quantum kernel* $\kappa_{\boldsymbol{\theta}}(\boldsymbol{x},\boldsymbol{x'})=\kappa^{FQ}_{\boldsymbol{\theta}}(\boldsymbol{x},\boldsymbol{x'})$*, we have*

$$\tilde{G}_n(\varepsilon_{\mathbb{U}_{\boldsymbol{\theta}}(\boldsymbol{x})},\varepsilon_{\mathbb{U}_{\boldsymbol{\theta}}(\boldsymbol{x'})})=\beta_{\text{Haar}}+\varepsilon_{\mathbb{U}_{\boldsymbol{\theta}}(\boldsymbol{x})}\varepsilon_{\mathbb{U}_{\boldsymbol{\theta}}(\boldsymbol{x'})}+\sqrt{\beta_{\text{Haar}}}\left(\varepsilon_{\mathbb{U}_{\boldsymbol{\theta}}(\boldsymbol{x})}+\varepsilon_{\mathbb{U}_{\boldsymbol{\theta}}(\boldsymbol{x'})}\right)\ , \tag{324}$$

   where $\beta_{\text{Haar}}=\frac{1}{2^{n-1}(2^n+1)}$.

2. *For the projected quantum kernel* $\kappa_{\boldsymbol{\theta}}(\boldsymbol{x},\boldsymbol{x'})=\kappa^{PQ}_{\boldsymbol{\theta}}(\boldsymbol{x},\boldsymbol{x'})$*, we have*

$$\tilde{G}_n(\varepsilon_{\mathbb{U}_{\boldsymbol{\theta}}(\boldsymbol{x})},\varepsilon_{\mathbb{U}_{\boldsymbol{\theta}}(\boldsymbol{x'})})=2\gamma n(2\tilde{\beta}_{\text{Haar}}+\varepsilon_{\mathbb{U}_{\boldsymbol{\theta}}(\boldsymbol{x})}+\varepsilon_{\mathbb{U}_{\boldsymbol{\theta}}(\boldsymbol{x'})})\ . \tag{325}$$

   where $\tilde{\beta}_{\text{Haar}}=\frac{3}{2^{n+1}+2}$.

Proof. The proof follows the same steps as the proof of the extension of Theorem 1 in Supplementary Note IV A with the integration over $\boldsymbol{x}$ and $\boldsymbol{x'}$ replaced with the integration over $\boldsymbol{\theta}$. □

B. Entanglement

We show that the entanglement generated via the parametrized data embedding can have a negative impact on the projected quantum kernels. Particularly, the following theorem generalizes Theorem 2 for the parametrized projected quantum kernel.

Supplemental Theorem 3. *Consider the parametrized projected quantum kernel* $\kappa^{PQ}_{\boldsymbol{\theta}}(\boldsymbol{x},\boldsymbol{x'})$*. Consider a pair of parametrized data-encoded states* $\rho(\boldsymbol{x},\boldsymbol{\theta})$ *and* $\rho(\boldsymbol{x'},\boldsymbol{\theta})$*, associated with* $\boldsymbol{x}$*,* $\boldsymbol{x'}$ *and* $\boldsymbol{\theta}$*. Then, we have*

$$\left|1-\kappa^{PQ}_{\boldsymbol{\theta}}(\boldsymbol{x},\boldsymbol{x'})\right|\leqslant(2\ln 2)\gamma\Gamma_s(\boldsymbol{x},\boldsymbol{x'},\boldsymbol{\theta})\ , \tag{326}$$

where

$$\Gamma_s(\boldsymbol{x},\boldsymbol{x'},\boldsymbol{\theta})=\sum_{k=1}^{n}\left[\sqrt{S\left(\rho_k(\boldsymbol{x},\boldsymbol{\theta})\middle\|\frac{\mathbb{1}_k}{2}\right)}+\sqrt{S\left(\rho_k(\boldsymbol{x'},\boldsymbol{\theta})\middle\|\frac{\mathbb{1}_k}{2}\right)}\right]^2\ , \tag{327}$$

where we denote $S(\cdot\|\cdot)$ *as the quantum relative entropy,* ρ_k *as a reduced state on qubit* k*, and* $\mathbb{1}_k$ *as the maximally mixed state on qubit* k*.*

Proof. The proof is the same as the proof of Theorem 2 in Supplementary Note V with $\rho(\boldsymbol{x})$ replaced with $\rho(\boldsymbol{x},\boldsymbol{\theta})$. □

C. Global measurements

We argue that the variational part of $U(\boldsymbol{x},\boldsymbol{\theta})$ should not contain global measurements. This is only relevant to the fidelity quantum kernel since its associated observable is global. On the other hand, global measurements have no impact on projected kernels due to their local construction.

To illustrate this, we consider the parametrized embedding of the form $U(\boldsymbol{x},\boldsymbol{\theta})=U_d(\boldsymbol{x})U_p(\boldsymbol{\theta})$ where $U_d(\boldsymbol{x})$ and $U_p(\boldsymbol{\theta})$ can be arbitrary. Supplemental Proposition 7 then shows that the variance of the parametrized kernel with respect to $\boldsymbol{\theta}$ is upper bounded by the variance of an expectation of some global observable.

Supplemental Proposition 7. *Consider the parametrized fidelity quantum kernels $\kappa^{FQ}_{\boldsymbol{\theta}}(\boldsymbol{x},\boldsymbol{x'})$ with the parametrized embedding $U(\boldsymbol{x},\boldsymbol{\theta}) = U_d(\boldsymbol{x})U_p(\boldsymbol{\theta})$ where $U_d(\boldsymbol{x})$ and $U_p(\boldsymbol{\theta})$ are the embedding and parametrized unitary blocks. In addition, consider the decomposition of $U_d^\dagger(\boldsymbol{x})U_d(\boldsymbol{x'})$ as $\mathcal{M}_R(\boldsymbol{x},\boldsymbol{x'}) + i\mathcal{M}_I(\boldsymbol{x},\boldsymbol{x'})$ with $\mathcal{M}_R(\boldsymbol{x},\boldsymbol{x'})$ and $\mathcal{M}_I(\boldsymbol{x},\boldsymbol{x'})$ being some Hermitian operators. Then, we have*

$$\mathrm{Var}_{\boldsymbol{\theta}}[\kappa^{FQ}_{\boldsymbol{\theta}}(\boldsymbol{x},\boldsymbol{x'})] \leqslant 4\mathrm{max}(\mathrm{Var}_{\boldsymbol{\theta}}[a_R], \mathrm{Var}_{\boldsymbol{\theta}}[a_I]) \ , \tag{328}$$

where $a_R = \mathrm{Tr}\big[\mathcal{M}_R(\boldsymbol{x},\boldsymbol{x'})U_p(\boldsymbol{\theta})\rho_0 U_p^\dagger(\boldsymbol{\theta})\big]$ and $a_I = \mathrm{Tr}\big[\mathcal{M}_I(\boldsymbol{x},\boldsymbol{x'})U_p(\boldsymbol{\theta})\rho_0 U_p^\dagger(\boldsymbol{\theta})\big]$ with an initial state ρ_0.

Proof. We are now ready to prove the proposition. Consider the decomposition of $\kappa^{FQ}_{\boldsymbol{\theta}}(\boldsymbol{x},\boldsymbol{x'})$ with an initial state $\rho_0 = |\psi_0\rangle\langle\psi_0|$

$$\kappa^{FQ}_{\boldsymbol{\theta}}(\boldsymbol{x},\boldsymbol{x'}) = |\langle\psi_0| U_p^\dagger(\boldsymbol{\theta})U_d^\dagger(\boldsymbol{x})U_d(\boldsymbol{x'})U_p(\boldsymbol{\theta})|\psi_0\rangle|^2 \ , \tag{329}$$

$$= |\langle\psi_0| U_p^\dagger(\boldsymbol{\theta})(\mathcal{M}_R(\boldsymbol{x},\boldsymbol{x'}) + i\mathcal{M}_I(\boldsymbol{x},\boldsymbol{x'}))U_p(\boldsymbol{\theta})|\psi_0\rangle|^2 \ , \tag{330}$$

$$= (\langle\psi_0| U_p^\dagger(\boldsymbol{\theta})\mathcal{M}_R(\boldsymbol{x},\boldsymbol{x'})U_p(\boldsymbol{\theta})|\psi_0\rangle)^2 + (\langle\psi_0| U_p^\dagger(\boldsymbol{\theta})\mathcal{M}_I(\boldsymbol{x},\boldsymbol{x'})U_p(\boldsymbol{\theta})|\psi_0\rangle)^2 \tag{331}$$

$$= a_R^2 + a_I^2 \ , \tag{332}$$

where we express $U_d^\dagger(\boldsymbol{x})U_d(\boldsymbol{x'})$ as $\mathcal{M}_R(\boldsymbol{x},\boldsymbol{x'})+i\mathcal{M}_I(\boldsymbol{x},\boldsymbol{x'})$ with $\mathcal{M}_R(\boldsymbol{x},\boldsymbol{x'})$ and $\mathcal{M}_I(\boldsymbol{x},\boldsymbol{x'})$ being some Hermitian matrices. We now upper bound the variance of $\kappa^{FQ}_{\boldsymbol{\theta}}(\boldsymbol{x},\boldsymbol{x'})$.

$$\mathrm{Var}_{\boldsymbol{\theta}}[\kappa^{FQ}_{\boldsymbol{\theta}}(\boldsymbol{x},\boldsymbol{x'})] = \mathrm{Var}_{\boldsymbol{\theta}}[a_R^2 + a_I^2] \tag{333}$$

$$\leqslant 2\mathrm{Var}_\theta[a_R^2] + 2\mathrm{Var}_\theta[a_I^2] \tag{334}$$

$$\leqslant 8|a_R|^2_{\mathrm{max}}\mathrm{Var}_{\boldsymbol{\theta}}[a_R] + 8|a_I|^2_{\mathrm{max}}\mathrm{Var}_{\boldsymbol{\theta}}[a_I] \tag{335}$$

$$\leqslant 8\mathrm{Var}_{\boldsymbol{\theta}}[a_R] + 8\mathrm{Var}_{\boldsymbol{\theta}}[a_I] \tag{336}$$

$$\leqslant 16\mathrm{max}(\mathrm{Var}_{\boldsymbol{\theta}}[a_R], \mathrm{Var}_{\boldsymbol{\theta}}[a_I]) \ , \tag{337}$$

where the first inequality is due to Lemma 10, the second inequality is due to Lemma 11 followed by $\mathbb{E}[X] \leqslant |X|_{\mathrm{max}}$, the third inequality comes from the fact that a_R and a_I are upper bounded by 1 (since $\|\mathcal{M}_R(\boldsymbol{x},\boldsymbol{x'})\|_\infty, \|\mathcal{M}_I(\boldsymbol{x},\boldsymbol{x'})\|_\infty \leqslant 1$), the last inequality is from choosing the maximum of the two terms. □

It follows from Supplemental Proposition 7 that if a_R and a_I exhibit barren plateaus (with respect to their implicit $\boldsymbol{\theta}$ dependence), then $\kappa^{FQ}_{\boldsymbol{\theta}}(\boldsymbol{x},\boldsymbol{x'})$ will also exhibit a barren plateau. Since a_I and a_R are linear expectation values of Hermitian operators $\mathcal{M}_R(\boldsymbol{x},\boldsymbol{x'})$ and $\mathcal{M}_I(\boldsymbol{x},\boldsymbol{x'})$, this allows us to apply barren plateaus results from QNNs to $\kappa^{FQ}_{\boldsymbol{\theta}}(\boldsymbol{x},\boldsymbol{x'})$. In particular, if $U_d^\dagger(\boldsymbol{x})U_d(\boldsymbol{x'})$ is global and $U_p(\boldsymbol{\theta})$ is a layer hardware efficient ansatz, the results in Ref. [31] for global costs imply $\kappa^{FQ}_{\boldsymbol{\theta}}(\boldsymbol{x},\boldsymbol{x'})$ exponentially concentrates around its mean.

D. Noise

Noise negatively affects the trainability of the parametrized quantum kernels, exponentially flattening the training landscape (with respect to $\boldsymbol{\theta}$) of $\tilde{\kappa}_{\boldsymbol{\theta}}(\boldsymbol{x},\boldsymbol{x'})$ and at the same time leading to the exponential concentration (with respect to $\boldsymbol{x},\boldsymbol{x'}$). The following theorem generalizes Theorem 3 to the noisy parametrized quantum kernels $\tilde{\kappa}_{\boldsymbol{\theta}}(\boldsymbol{x},\boldsymbol{x'})$

First, we specify the parametrized data embedding to be in the following form

$$U(\boldsymbol{x},\boldsymbol{\theta}) = \prod_{l=1}^{L} U_l(\boldsymbol{x}_l,\boldsymbol{\theta}_l) \tag{338}$$

We consider the same local Pauli noise model as described in where the noise acts before and after each layer Eq. (28) in the main text with the noise characteristic q.

Supplemental Theorem 4. *Consider the L-layered parametrized data embedding defined in Eq. (338) with input state ρ_0 and the layer-wise Pauli noise model defined in Eq. (28) in the main text with characteristic noise parameter $q \leqslant 1$. The concentration of quantum kernel values may be bounded as follows*

$$|\tilde{\kappa}_{\boldsymbol{\theta}}(\boldsymbol{x}, \boldsymbol{x'}) - \mu| \leqslant F(q, L) \; . \tag{339}$$

1. *For the fidelity quantum kernel $\tilde{\kappa}_{\boldsymbol{\theta}}(\boldsymbol{x}, \boldsymbol{x'}) = \tilde{\kappa}^{FQ}_{\boldsymbol{\theta}}(\boldsymbol{x}, \boldsymbol{x'})$, we have $\mu = 1/2^n$, and*

$$F(q, L) = q^{2L+1} \left\| \rho_0 - \frac{\mathbb{1}}{2^n} \right\|_2 \; . \tag{340}$$

2. *For the projected quantum kernel $\tilde{\kappa}_{\boldsymbol{\theta}}(\boldsymbol{x}, \boldsymbol{x'}) = \tilde{\kappa}^{PQ}_{\boldsymbol{\theta}}(\boldsymbol{x}, \boldsymbol{x'})$, we have $\mu = 1$, and*

$$F(q, L) = (8 \ln 2) \gamma n q^{b(L+1)} S_2 \left(\rho_0 \middle\| \frac{\mathbb{1}}{2^n} \right) \; , \tag{341}$$

where $S_2(\cdot\|\cdot)$ denotes the sandwiched 2-Rënyi relative entropy and $b = 1/(2\ln(2)) \approx 0.72$.

Additionally, the noisy data-encoded quantum state $\tilde{\rho}(\boldsymbol{x}, \boldsymbol{\theta})$ concentrates towards the maximally mixed state as

$$\left\| \tilde{\rho}(\boldsymbol{x}, \boldsymbol{\theta}) - \frac{\mathbb{1}}{2^n} \right\|_2 \leqslant q^{L+1} \left\| \rho_0 - \frac{\mathbb{1}}{2^n} \right\|_2 \; . \tag{342}$$

Proof. The proof is the same as the proof of Theorem 3 in Supplementary Note VII with $\tilde{\rho}(\boldsymbol{x})$ replaced with $\tilde{\rho}(\boldsymbol{x}, \boldsymbol{\theta})$. □

---

[1] H.-Y. Huang, M. Broughton, M. Mohseni, R. Babbush, S. Boixo, H. Neven, and J. R. McClean, Power of data in quantum machine learning, Nature Communications **12**, 1 (2021).
[2] L. Slattery, R. Shaydulin, S. Chakrabarti, M. Pistoia, S. Khairy, and S. M. Wild, Numerical evidence against advantage with quantum fidelity kernels on classical data, Phys. Rev. A **107**, 062417 (2023).
[3] J. Kübler, S. Buchholz, and B. Schölkopf, The inductive bias of quantum kernels, Advances in Neural Information Processing Systems **34**, 12661 (2021).
[4] R. Shaydulin and S. M. Wild, Importance of kernel bandwidth in quantum machine learning, Physical Review A **106**, 042407 (2022).
[5] A. Canatar, E. Peters, C. Pehlevan, S. M. Wild, and R. Shaydulin, Bandwidth enables generalization in quantum kernel models, arXiv preprint arXiv:2206.06686 (2022).
[6] Y. Suzuki, H. Kawaguchi, and N. Yamamoto, Quantum fisher kernel for mitigating the vanishing similarity issue, arXiv preprint arXiv:2210.16581 (2022).
[7] T. Hubregtsen, D. Wierichs, E. Gil-Fuster, P.-J. H. Derks, P. K. Faehrmann, and J. J. Meyer, Training quantum embedding kernels on near-term quantum computers, Physical Review A **106**, 042431 (2022).
[8] M. Incudini, F. Martini, and A. Di Pierro, Structure learning of quantum embeddings, arXiv preprint arXiv:2209.11144 (2022).
[9] Y. Liu, S. Arunachalam, and K. Temme, A rigorous and robust quantum speed-up in supervised machine learning, Nature Physics , 1 (2021).
[10] G. Gentinetta, A. Thomsen, D. Sutter, and S. Woerner, The complexity of quantum support vector machines, arXiv preprint arXiv:2203.00031 (2022).
[11] A. B. Tsybakov, *Introduction to Nonparametric Estimation* (Springer, 2009).
[12] H.-Y. Huang, R. Kueng, and J. Preskill, Predicting many properties of a quantum system from very few measurements, Nature Physics **16**, 1050 (2020).
[13] A. Elben, S. T. Flammia, H.-Y. Huang, R. Kueng, J. Preskill, B. Vermersch, and P. Zoller, The randomized measurement toolbox, Nature Review Physics 10.1038/s42254-022-00535-2 (2022).
[14] J. R. Glick, T. P. Gujarati, A. D. Corcoles, Y. Kim, A. Kandala, J. M. Gambetta, and K. Temme, Covariant quantum kernels for data with group structure, arXiv preprint arXiv:2105.03406 (2021).

[15] D. A. Roberts and B. Yoshida, Chaos and complexity by design, Journal of High Energy Physics **2017**, 121 (2017).

[16] S. Wang, E. Fontana, M. Cerezo, K. Sharma, A. Sone, L. Cincio, and P. J. Coles, Noise-induced barren plateaus in variational quantum algorithms, Nature Communications **12**, 1 (2021).

[17] C. Hirche, C. Rouzé, and D. S. França, On contraction coefficients, partial orders and approximation of capacities for quantum channels, arXiv preprint arXiv:2011.05949 (2020).

[18] S. Wang, P. Czarnik, A. Arrasmith, M. Cerezo, L. Cincio, and P. J. Coles, Can error mitigation improve trainability of noisy variational quantum algorithms?, Quantum **8**, 1287 (2024).

[19] R. Takagi, S. Endo, S. Minagawa, and M. Gu, Fundamental limits of quantum error mitigation, npj Quantum Information **8**, 114 (2022).

[20] Y. Quek, D. S. França, S. Khatri, J. J. Meyer, and J. Eisert, Exponentially tighter bounds on limitations of quantum error mitigation, arXiv preprint arXiv:2210.11505 (2022).

[21] Y. Li and S. C. Benjamin, Efficient variational quantum simulator incorporating active error minimization, Phys. Rev. X **7**, 021050 (2017).

[22] K. Temme, S. Bravyi, and J. M. Gambetta, Error mitigation for short-depth quantum circuits, Physical review letters **119**, 180509 (2017).

[23] S. Endo, S. C. Benjamin, and Y. Li, Practical quantum error mitigation for near-future applications, Physical Review X **8**, 031027 (2018).

[24] A. Kandala, K. Temme, A. D. Córcoles, A. Mezzacapo, J. M. Chow, and J. M. Gambetta, Error mitigation extends the computational reach of a noisy quantum processor, Nature **567**, 491–495 (2019).

[25] P. Czarnik, A. Arrasmith, P. J. Coles, and L. Cincio, Error mitigation with Clifford quantum-circuit data, Quantum **5**, 592 (2021).

[26] W. J. Huggins, S. McArdle, T. E. O'Brien, J. Lee, N. C. Rubin, S. Boixo, K. B. Whaley, R. Babbush, and J. R. McClean, Virtual distillation for quantum error mitigation, Physical Review X **11**, 041036 (2021).

[27] B. Koczor, Exponential error suppression for near-term quantum devices, Physical Review X **11**, 031057 (2021).

[28] M. Cerezo, A. Arrasmith, R. Babbush, S. C. Benjamin, S. Endo, K. Fujii, J. R. McClean, K. Mitarai, X. Yuan, L. Cincio, and P. J. Coles, Variational quantum algorithms, Nature Reviews Physics **3**, 625–644 (2021).

[29] S. Endo, Z. Cai, S. C. Benjamin, and X. Yuan, Hybrid quantum-classical algorithms and quantum error mitigation, Journal of the Physical Society of Japan **90**, 032001 (2021).

[30] M. Kline, *Calculus: An Intuitive and Physical Approach* (Dover publications, INC., 1998).

[31] M. Cerezo, A. Sone, T. Volkoff, L. Cincio, and P. J. Coles, Cost function dependent barren plateaus in shallow parametrized quantum circuits, Nature Communications **12**, 1 (2021).
